# Worldwide Regulations of Standard Values of Pesticides for Human Health Risk Control: A Review

**DOI:** 10.3390/ijerph14070826

**Published:** 2017-07-22

**Authors:** Zijian Li, Aaron Jennings

**Affiliations:** 1 Department of Civil Engineering, Case Western Reserve University, Cleveland, OH 44106, USA; aaj2@case.edu; 2 Parsons Corporation, Chicago, IL 60606, USA

**Keywords:** pesticide regulation, pesticide exposure, human health risk assessment, health risk uncertainty bounds, environmental regulatory jurisdiction

## Abstract

The impact of pesticide residues on human health is a worldwide problem, as human exposure to pesticides can occur through ingestion, inhalation, and dermal contact. Regulatory jurisdictions have promulgated the standard values for pesticides in residential soil, air, drinking water, and agricultural commodity for years. Until now, more than 19,400 pesticide soil regulatory guidance values (RGVs) and 5400 pesticide drinking water maximum concentration levels (MCLs) have been regulated by 54 and 102 nations, respectively. Over 90 nations have provided pesticide agricultural commodity maximum residue limits (MRLs) for at least one of the 12 most commonly consumed agricultural foods. A total of 22 pesticides have been regulated with more than 100 soil RGVs, and 25 pesticides have more than 100 drinking water MCLs. This research indicates that those RGVs and MCLs for an individual pesticide could vary over seven (DDT drinking water MCLs), eight (Lindane soil RGVs), or even nine (Dieldrin soil RGVs) orders of magnitude. Human health risk uncertainty bounds and the implied total exposure mass burden model were applied to analyze the most commonly regulated and used pesticides for human health risk control. For the top 27 commonly regulated pesticides in soil, there are at least 300 RGVs (8% of the total) that are above all of the computed upper bounds for human health risk uncertainty. For the top 29 most-commonly regulated pesticides in drinking water, at least 172 drinking water MCLs (5% of the total) exceed the computed upper bounds for human health risk uncertainty; while for the 14 most widely used pesticides, there are at least 310 computed implied dose limits (28.0% of the total) that are above the acceptable daily intake values. The results show that some worldwide standard values were not derived conservatively enough to avoid human health risk by the pesticides, and that some values were not computed comprehensively by considering all major human exposure pathways.

## 1. Introduction

Pesticides are broadly applied in numerous agricultural, commercial, residential, and industrial applications to control and kill pests. They help society fight disease and increase agricultural productivity; however, pesticides can be transported into the air, water, soil, and biomass after numerous applications and can cause risks to the ecosystem and to human health. The impact of pesticide residues on human health is a worldwide problem, as human exposure to pesticides can occur through the ingestion of pesticide-contaminated water, food, or residential surface soil, the inhalation of pesticide-contaminated air, soil dust, or industrial vapor, and dermal contact with pesticide-contaminated water (swimming, showering, or raining), air, agricultural commodities, or soil. Worldwide jurisdictions have been working on regulating pesticide standard values for residential surface soil, residential air, drinking water, surface water, groundwater, and food for years.

Pesticide soil regulatory guidance values (RGVs) are applied by worldwide soil jurisdictions to control pesticide pollution in residential surface soil. Pesticide soil RGVs specified the maximum amount of a pesticide which might be present in the soil without prompting regulatory responses, such as surface or groundwater contamination by the transport of pesticides from surface soil, ecological risk, and adverse human health effects by exposure to soil pesticides. The most concerned and conservative pesticide soil RGVs are provided for residential surface soil, where children can be exposed to soil pesticides by the ingestion of soil, the inhalation of soil dust, or dermal contact. Although many worldwide regulatory jurisdictions have provided the RGVs in soil to protect human health, there is a lack of agreement on the pesticides that need to be regulated, as well as the magnitude of the pesticide soil RGVs which should be applied to a certain pesticide. For some of the most frequently regulated pesticides, the RGVs vary to above six orders of magnitude (i.e. 1,000,000) [1]. This variability implies that worldwide soil regulatory jurisdictions have hugely different views on the criteria which cause significant human health risks by residential surface soil pesticides. Other studies have also investigated soil RGVs, but have had their evaluations restricted to less-extensive sets of jurisdictions, such as the U.S. and European nations [2,3,4,5,6,7,8].

Drinking water supplies might be contaminated by pesticides as pesticide can be transferred into surface water or ground water systems, which are usually considered as important drinking water sources. The pesticides found in drinking water may have a potential impact on human health, depending on the amount and the toxicity of the pesticides and the frequency/length of human exposure to the contaminated drinking water. Similar to the pesticide soil RGVs, pesticide drinking water maximum concentration levels (MCLs) are also established by worldwide regulatory jurisdictions to specify the maximum allowable concentration of pesticides in drinking water to protect human health. The results indicate that there is little agreement on both how the pesticides should be regulated and what the magnitude of the MCLs applied to a certain pesticide should be among worldwide drinking water regulatory jurisdictions. The analysis [1] demonstrated that the MCLs of the most commonly regulated pesticides often vary by five, six, or even seven orders of magnitude, which also indicated that some extremely large pesticide MCL values are unlikely to protect human health. The California Department of Public Health (2013) [9] compared the MCLs to the U.S. Environmental Protection Agency (EPA) standard. Among others [10,11], Bamidele (2015) studied the MCLs among the Canadian, European Union (EU), World Health Organization (WHO), U.S., and Nigerian national standards.

Since pesticides are directly applied on crops, fruits, and vegetables in most agricultural applications, infants, children, and adults can be exposed to pesticides by the ingestion of those pesticide-contaminated foods. Pesticide maximum residue limits (MRLs), which specify the maximum concentration of a pesticide that can exist in certain agricultural commodities, were regulated by many nations to promote good agricultural practice (GAP). Because food consumption varies by season, geology, culture, personal habit, economic status and crop availability, which all have impacts on human exposure to pesticides, it is crucial and challenging to develop pesticide MRLs for numerous agricultural commodities to protect human health. To evaluate the effectiveness of the protection from the pesticide MRLs for various agricultural foods, estimations of the most commonly consumed agricultural commodities and the ingestion rate are necessary. Therefore, an implied exposure dose (IED) was introduced in this research to convert the pesticide MRLs in the most consumed agricultural commodities into the pesticide exposure mass burden, and to compare with the toxicology data. The results indicated that many MRLs for the most widely used pesticides were set too high to protect human health.

Pesticides can exist in residential air by the evaporation of volatile and semi-volatile pesticides, such as organochlorine pesticides, from crops and residential surface soil. In addition, pesticides can be blown away from agricultural fields by the wind, and some fumigants (e.g., bromomethane) are released into the air in a gaseous form. Therefore, the regulation of pesticide standard values in the residential air is necessary to control human health risks through inhalation and dermal contact exposures, especially for volatile and semi-volatile pesticides. However, few worldwide jurisdictions have regulated pesticide air standard values, which means that people around the world are probably not protected by the pesticide air regulations, especially for some farmers and workers who frequently work in the agricultural field.

Human exposures to pesticide may also occurs through swimming in rivers, lakes, or pools where the water has been contaminated by pesticides, taking a shower when the water is being pumped from pesticide-contaminated ground water, getting wet from pesticide-contaminated rainwater, or handling pesticide-related products during work. Since the regulation of pesticides in these scenarios is important to control human health risks, and since these scenarios are the four most frequent human exposure pathways for pesticides, the pesticide standard values in major human exposure pathways which include residential soil, drinking water, agricultural commodities, and residential air were discussed in this research.

The standard values for pesticides for each human exposure pathway should be derived by laboratory toxicology data and human health risk models. Some uncertainty and marginal safety factors should also be applied to allow for additional exposure possibilities. As pesticide exposures in major exposure scenarios always happen simultaneously, it is necessary to derive and allocate pesticide standard values in major exposure pathways comprehensively. Therefore, the implied maximum dose limit (IMDL) was applied to compute the total maximum exposure mass burden for a certain pesticide from the pesticide standard values of the national jurisdictions in all major exposure pathways. The objectives of this research review are to evaluate current worldwide pesticide standard values in major exposures, examine whether those standard values can protect human health, and help environmental regulatory jurisdictions to rationalize their pesticide standard values by a scope of worldwide efforts.

## 2. Materials

The materials for this research review include two main parts; one is the pesticide which had been regulated with a certain residential soil RGV, drinking water MCL, agricultural commodity MRL, or residential air MCL, and the other is the worldwide regulatory jurisdiction which had promulgated a certain pesticide standard value in any of those major exposure pathways.

### 2.1. Pesticide

The World Health Organization (WHO) (2017) [12] defined a pesticide as a chemical compound that is used to kill pests, including insects, rodents, fungi and unwanted plants (weeds). The Food and Agriculture Organization (FAO) of the United Nations defined a pesticide as any substance or mixture of substances intended for preventing, destroying, or controlling any pest, including vectors of human or animal diseases, unwanted species of plants or animals causing harm during, or otherwise interfering with, the production, processing, storage, or marketing of food, agricultural commodities, wood and wood products, or animal feedstuffs, or which may be administered to animals for the control of insects, arachnids or other pests in or on their bodies.

Pesticides can be classified by target groups as acaricides, avicides, bactericides, herbicides, fungicides, insecticides, repellents, virucides, and so on. According to the chemical compositions of the active ingredients, pesticides can be categorized into four main groups: carbamates, organochlorines, organophosphorus, and pyrethrin and pyrethroids. WHO (2009) [13] classified pesticides by hazard as an extremely hazardous pesticide, a highly hazardous pesticide, a moderately hazardous pesticide, a slightly hazardous pesticide, and a pesticide which is unlikely to present an acute hazard. In addition, based on the mode of formulation, pesticides can be classified as emulsifiable concentrates, wettable powders, granules, baits, dust, and fumigants [14].

### 2.2. CAS No.

Because of the complex chemical structures and the chemical complexity of pesticides and their active ingredients, pesticides are often regulated by their trade names instead of the chemical nomenclature conventions. For example, the National Institute of Standards and Technology (NIST) (2011) [15] listed 120 names for the pesticide “Lindane”, which include 30 chemical nomenclature names and 90 other trade names. Even worldwide jurisdictions have regulated pesticides by their local trade names in foreign languages, which has made it difficult to identify pesticides by their “names”. The Chemical Abstracts Service Registry Number (CAS No.) by NIST and the common name by the International Union of Pure and Applied Chemistry (IUPCA) were applied to identify pesticides in this research review, as each pesticide was assigned a unique CAS No. and a common name. It would be helpful and convenient to the public if a jurisdiction were to use the CAS No. to identify regulated pesticides; however, the CAS No. is not available for most worldwide jurisdictions beyond Europe and North America.

### 2.3. Sources for Worldwide Pesticide Soil RGVs and Drinking Water MCLs

Pesticide soil RGVs and drinking water MCLs were directly taken from the regulatory jurisdictions and most were obtained from the official government websites, which are the primary sources. Sometimes the primary source was not available when the official documents were too old to have an online version when access was needed for the document, when the official website was under maintenance, and when the jurisdiction needed to be purchased. For these cases, secondary sources such as newspapers, annual reports, research articles, conferences, and government statements were applied to obtain the pesticide standard values. Since many international regulatory jurisdictions were written in foreign languages, the Google translate online tool was used to translate the foreign language documents into English. The reference websites for the worldwide and U.S. pesticide soil RGVs and drinking water MCLs and the pesticide standard values used in this study were provided in supplement materials (Tables S1–S4 and Database). When web addresses and online documents become out of date and inactive, keywords from the document title would help to address the new web location by using web search engines.

Table 1 lists the worldwide nations, regions, territories, and organizations which had provided the pesticide soil or drinking water regulatory jurisdictions, their sources, the numbers of pesticide standard values, and the languages applied. A total of 4590 pesticide soil RGVs were identified by 108 international jurisdictions from 54 United Nation (UN) members, three multi-national organizations, and two non-UN members outside of the U.S. At least 3534 pesticide drinking water MCLs were identified from 130 international jurisdictions from 102 UN members, four multi-national organizations, and two non-UN members outside of the U.S. There were more nations which provided pesticide standard values in drinking water than for residential soil, indicating that more nations focused on pesticide regulations for drinking water. Pesticides RGVs and MCLs were also identified by the U.S. jurisdictions from national organizations, states, cities, U.S. territories, and Autonomous Native American Tribes (see Table 2). There were 14,831 pesticides RGVs identified from 66 U.S. soil regulatory jurisdictions, including 46 of the 50 states, six national organizations, five regions (cities and counties), two U.S. territories, and seven Autonomous Native American Tribes. In addition, a total of 1940 pesticide MCLs were identified by 61 U.S. drinking water jurisdictions, from 48 of the 50 states, three national organizations, and two U.S. territories. Only four states, including North Dakota, South Carolina, South Dakota and Utah, did not provide pesticide soil RGVs, and two states—Georgia and Washington—did not provide pesticide drinking water MCLs.

### 2.4. Sources for Worldwide Pesticide Agricultural Commodity MRLs

Pesticide agricultural commodity MRLs of worldwide nations were collected by the global MRL database (2014) [16], which included at least 90 nations and multi-national organizations (see Table 3). Since hundreds of agricultural commodities and pesticides were regulated and collected in this database, only the currently most widely used pesticides and the most consumed agricultural commodities were used in this research to compute the IED. The most widely used pesticides and consumed foods were selected based on worldwide pesticide usage and the consumption of foods, which was investigated for the nations where agricultural plays a significant role, such as China [17,18], India [19], the Philippines [20], Germany [21], the United Kingdom [22], Canada [23,24], the U.S. [25,26,27], Mexico [28], Costa Rica [29], Brazil [30,31], New Zealand [32], Australia [33,34], and South Africa [35]. The 14 current most widely used pesticides selected in this research review were 2,4-D, Aldicarb, Atrazine, Chlorothalonil, Chlorpyriphos, Diazinon, Dicamba, Diuron, Glyphosate, Malathion, Mancozeb, MCPA, Metolachlor, and Trifluralin. The most consumed agricultural commodities selected in this study were classified into four groups: grain crops (corn, wheat, and rice), vegetable crops (tomato, onion, and potato), fruit crops (apple, bananas, grape, and orange), and drinks (coffee bean, and tea leaves). Although pesticides can be transported and accumulate into meat and dairy products such as beef and fish, agricultural commodities are often exposed to pesticides directly, due to application methods such as spraying. The amount of pesticide accumulated in livestock always depends on the living environment, feeding stuff, and the metabolism of the animals. Compared to the pesticide exposure from the meat consumption, the pesticide exposure risk from agricultural commodities is much higher due to its broader application. Thus, pesticide exposure from the consumption of meat and dairy products will not be discussed in this study; however, marginal safety factors should be accounted for to allow additional exposures.

### 2.5. Sources for Pesticide Residential Air MCLs

Few jurisdictions in the world had regulated pesticide residential air MCLs; only the U.S. [36] regulated and derived the pesticide air MCLs systematically. Cancer and non-cancer human health risk models were developed by the U.S Environmental Protection Agency (USEPA), and the MCLs for 43 volatile and semi-volatile pesticides were promulgated. It is necessary to regulate pesticide air MCLs as the human health risks are raised by inhalation, skin contact, and even eye contact for pesticide-contaminated air, especially for farmers and workers who work in forests and the agricultural field. The regulation of pesticides in the air can also protect the ecosystem, wildlife, and livestock. Because of the lack of information available for worldwide pesticide air MCLs, regulatory pesticide standard values for residential air are omitted in this study.

## 3. Methods

### 3.1. Statistical Analysis of Pesticide Standard Values 

Since most data sets for pesticide standard values resemble a log-transformed random distribution [37,38,39,40,41], the cumulative distribution function was applied to plot the pesticide standard values and to compare it with the cumulative distribution of a log-normal random variable with identical statistics, arithmetic, mean, and standard deviations. The cumulative distribution function was used to illustrate how worldwide pesticide standard values dispersed over the span of values and the ranking of the standard values for each jurisdiction. The empirical cumulative distributions generated from pesticide standard value sets were generated as follows,

(1) $$P\left(X_{r}\leq X_{i}\right)\approx \frac{n_{i}}{N};\;\forall i=1,\ldots ,N$$

where X_r_ is a random value for a pesticide RGV, MCL, IED, IMDL, or the number of pesticide standard values which a jurisdiction had provided, X_i_ is the known value for the same pesticide, and ni is the integer rank of X_i_ in the N known values.

To examine if the degree of the empirical distribution resembled the log-normal random distribution of a Pearson (r) correlation, the analysis was conducted as follows,

(2) $$r=\frac{N[\sum E(X_{i})\times F\left(X_{i}\right)]-[\sum E(X_{i})]\times [\sum F\left(X_{i}\right)]}{\sqrt{\left[N\sum E{\left(X_{i}\right)}^{2}-{\left(\sum E(X_{i}\right)}^{2}\right]\times \left[N\sum F{\left(X_{i}\right)}^{2}-{\left(\sum \text{​}F\left(X_{i}\right)\right)}^{2}\right]}}$$

where E(X_i_) is the probability computed from the empirical distribution, and F(X_i_) is the probability calculated for the log-normal cumulative distribution.

Since some jurisdictions shared the same standard value for a certain pesticide, non-random values always appeared in empirical distributions. A data value cluster was introduced in this study and defined as a data interval (X_i_ − X_i+Y_), containing Y values which did not occur randomly. The probability (P_c_) of a randomly occurring data value cluster was quantified by the binomial probability function as follows,

(3) $$P_{c}[Y\;X\varepsilon (X_{i},X_{i+Y})]=\left[\frac{N!}{Y!\times \left(N-Y\right)!}\right]\times {\left[F\left(X_{i+Y}\right)-F\left(X_{i}\right)\right]}^{Y}\times {\left\{1-\left[F\left(X_{i+Y}\right)-F\left(X_{i}\right)\right]\right\}}^{N-Y}$$

where F(X_i_) and F(X_i+Y_) were computed from the log-normal cumulative distribution. A probability of less than 0.001 indicated that the data value cluster did not occur randomly.

### 3.2. Human Health Risk Models

Many regulatory jurisdictions developed standard values for non-genotoxic pesticides, “thresholded toxicants” [42], or “systemic toxicants” [43] based on the acceptable daily intake (ADI), tolerable daily intake (TDI), or reference dose (RfD) [43]. The standard values are defined as the level of toxicant exposure on a daily or weekly basis without adverse health effects or an appreciable health risk over a lifetime. The ADI or TDI was usually converted from the no observed adverse effect level (NOAEL), or the lowest observed effect level (LOEL) by laboratory animal experiments by applying an uncertainty factor which allows for interspecies differences and human variability, expressed as follows,

(4) $$\mathrm{ADI}\;\left(\mathrm{TDI},\;\mathrm{or}\;\mathrm{RfD}\right)=\frac{\mathrm{NOAEL}\;\left(\mathrm{or}\;\mathrm{LOEL}\right)}{\mathrm{UF}}$$

where UF is the uncertainty factor and usually applies to a 100-fold increase [42].

Based on the toxicology data, the pesticide soil RGVs should be developed based on exposure scenarios. To evaluate if the pesticide soil RGVs are conservative enough to protect human health, human health cancer and non-cancer risk models (Equations (5)–(12)) developed by the USEPA [36] were applied in this research to compute cancer and non-cancer risk uncertainty bounds. The USEPA models considered all pesticide soil exposure scenarios, such as soil ingestion, soil dust inhalation, and soil dermal contact. There is an uncertainty when using the USEPA models to examine soil RGVs. This does not mean that the USEPA models are universal; every jurisdiction can develop and conduct an RGV risk assessment and evaluation based on their situations. The USEPA summarized the toxicity and chemical-specific information of pesticides, which included the chronic oral slope factor (CSF_o_) (kg-mg/day), the fraction of contaminant absorbed in gastrointestinal tract (GIABS) (unitless), the fraction of contaminant absorbed dermally from soil (ABS_d_) (unitless), the chronic inhalation unit risk (IUR) (m^3^/ug), the volatilization factor (VF_s_) (m^3^/kg), the chronic oral reference dose (RfD_o_) (mg/kg-day), and the chronic inhalation reference concentration (RfC) (mg/m^3^) [36]. The range of the exposure coefficients [44] applied by the U.S. states were defined in the following equations.

The pesticide soil RGV (mg/kg) equation derived by cancer risk equations was expressed as follows,

(5) $$\mathrm{RGV}=\frac{1}{\frac{1}{{\mathrm{RSL}}_{\mathrm{cancer}-\mathrm{ingestion}}}+\frac{1}{{\mathrm{RSL}}_{\mathrm{cancer}-\mathrm{inhalation}}}+\frac{1}{{\mathrm{RSL}}_{\mathrm{cancer}-\mathrm{dermal}}}}$$

where the RSL (mg/kg) is the reginal screen level derived by the cancer risk equations of soil ingestion (Equation (6)), soil dermal contact (Equation (7)), and soil dust inhalation (Equation (8)).

(6) $${\mathrm{RSL}}_{\mathrm{cancer}-\mathrm{ingestion}}=\frac{\mathrm{TR}\times \mathrm{AT}\times \mathrm{LT}}{{\mathrm{CSF}}_{0}\times {\mathrm{IFS}}_{\mathrm{adj}}\times {\mathrm{EF}}_{r}\times 10^{-6}}$$

TR—Target risk (1 × 10^−6^ unit less)
AT—Averaging time (365 days/year)
LT—Lifetime (70 years) (70, 75)
EF—Exposure frequency (350 days/year) (143, 365)
IFS_adj_—Resident soil ingestion rate (114 mg-year/kg-day) (87, 127)
  (7) $${\mathrm{RSL}}_{\mathrm{cancer}-\mathrm{dermal}}=\frac{\mathrm{TR}\times \mathrm{AT}\times \mathrm{LT}}{\left[\frac{{\mathrm{CSF}}_{0}}{\mathrm{GIABS}}\right]\times {\mathrm{EF}}_{r}\times {\mathrm{DFS}}_{\mathrm{adj}}\times {\mathrm{ABS}}_{d}\times 10^{-6}}$$
DFS_adj_—Resident soil dermal contact factor (360.8 mg-year/kg-day), (253, 1257)
  (8) $${\mathrm{RSL}}_{\mathrm{cancer}-\mathrm{inhalation}}=\frac{\mathrm{TR}\times \mathrm{AT}\times \mathrm{LT}}{\mathrm{IUR}\times {\mathrm{EF}}_{r}\times \mathrm{ED}\times \mathrm{ET}\times \left[\frac{1}{{\mathrm{VF}}_{s}}+\frac{1}{{\mathrm{PEF}}_{W}}\right]\times \left(\frac{1000}{24}\right)}$$
PEF_w_—Particulate emission factor (1.4 × 10^9^ m^3^/kg), (7.8 × 10^7^–6.6 × 10^9^)
ED—Exposure duration (30 years)
ET—Exposure time (24 h/day), (2, 24)
The pesticide soil RGV (mg/kg) non-cancer risk equations were expressed as follows,
  (9) $$\mathrm{RGV}=\frac{1}{\frac{1}{{\mathrm{RSL}}_{\mathrm{non}-\mathrm{cancer}-\mathrm{ingestion}}}+\frac{1}{{\mathrm{RSL}}_{\mathrm{non}-\mathrm{cancer}-\mathrm{dermal}}}+\frac{1}{{\mathrm{RSL}}_{\mathrm{non}-\mathrm{cancer}-\mathrm{inhalation}}}}$$
  (10) $${\mathrm{RSL}}_{\mathrm{non}-\mathrm{cancer}-\mathrm{ingestion}}=\frac{\mathrm{THQ}\times {\mathrm{AT}}_{r}\times {\mathrm{ED}}_{c}\times {\mathrm{BW}}_{c}}{{\mathrm{EF}}_{r}\times {\mathrm{ED}}_{c}\times \frac{1}{{\mathrm{RfD}}_{0}}\times {\mathrm{IRS}}_{c}\times 10^{-6}}$$
THQ—Target hazard quotient (1.0 unit less)
ED_c_—Exposure duration for child (6 year), (5, 7)
HW_c_—Human weight for child (15 kg), (15, 17)
IRS—Soil ingestion rate for child (200 mg/day), (100, 200)
  (11) $${\mathrm{RSL}}_{\mathrm{non}-\mathrm{cancer}-\mathrm{dermal}}=\frac{\mathrm{THQ}\times {\mathrm{AT}}_{r}\times {\mathrm{ED}}_{c}\times {\mathrm{BW}}_{c}}{{\mathrm{EF}}_{r}\times {\mathrm{ED}}_{c}\times \frac{1}{{\mathrm{RfD}}_{0}\times \mathrm{GIABS}}\times {\mathrm{SA}}_{c}\times {\mathrm{AF}}_{c}\times {\mathrm{ABS}}_{d}\times 10^{-6}}$$
SA_c_—Soil surface area for child (2800 cm^2^), (1750, 2960)
AF_c_—Soil adhesion factor for child (0.2 mg/cm^2^), (0.2, 1.0)
  (12) $${\mathrm{RSL}}_{\mathrm{non}-\mathrm{cancer}-\mathrm{inhalation}}=\frac{\mathrm{THQ}\times {\mathrm{AT}}_{r}\times {\mathrm{ED}}_{c}}{{\mathrm{EF}}_{r}\times {\mathrm{ED}}_{c}\times {\mathrm{ET}}_{\mathrm{rs}}\times \frac{1}{24}\times \frac{1}{\mathrm{RfC}}\times \left(\frac{1}{{\mathrm{VF}}_{s}}+\frac{1}{{\mathrm{PEF}}_{W}}\right)}$$

Pesticide drinking water MCLs and human health uncertainty risk bounds were based on the exposure scenario of ingestion. The MCL uncertainty risk bounds depend on the variation of the parameters in Equation (13),

(13) $$\mathrm{MCL}=\frac{\mathrm{ADI}\times \mathrm{HW}\times \mathrm{PF}}{V}$$

where HW is human body weight (kg), which is referred to as an average adult human weight in many jurisdictions. Some jurisdictions, such as the Australian Government [45], applied 70 kg and others, such as WHO [46], used 60 kg. PF is a proportion factor which quantifies the portion of the total pesticide exposure that is allocated to the drinking water ingestion pathway, which usually ranges from 0.1 to 1.0 [46]. V is the daily drinking water intake rate (L/day), and for most worldwide jurisdictions, 2.0 L/day was used, while for some nations with a cold climate, 1.5 L/day was applied [47].

### 3.3. Pesticide Agricultural Commodity Implied Exposure Dose (IED)

Pesticide IED was introduced to compute the implied pesticide mass burden from the most consumed agricultural commodities, and was compared to the ADI value of the same pesticide. The agricultural food intake rates were estimated in Equation (14) by the U.S. Department of Agriculture (USDA) [48] annual food consumption database. It should be noticed that agricultural commodity consumptions vary by season, geology, culture, personal habit, economic status and crop availability. The application of the USDA is one of the methods to estimate consumption rates. Table 3 lists the estimated agricultural food intake rate.

(14) $${\mathrm{FIR}}_{j}=\frac{M_{j}\times \frac{1\;\mathrm{year}}{365\;\mathrm{day}}}{P}$$

where FIR_j_ is the estimated intake rate of the agricultural commodity j, M_j_ is the total mass of agricultural commodity j consumed annually in the U.S. (kg/year), and P is the U.S. population (318.9 million for 2014).

The IED for the most widely used pesticides was expressed as follows,

(15) $$\mathrm{IEDi}=\overset{n}{\underset{j}{\sum }}\frac{{\mathrm{MRL}}_{\mathrm{ij}}\times {\mathrm{FIR}}_{j}\times \mathrm{EF}}{\mathrm{HW}}$$

where the IED_i_ is the implied exposure dose computed for the pesticides i (mg/kg-day), HW is the average adult human weight (kg), and EF is the exposure factor (unit less). The calculated results in this study were based on an average adult weight of 70 kg and an EF of 1.0 [49].

### 3.4. Implied Maximum Dose Limit 

Since human exposure to pesticides always occurs in different exposure pathways simultaneously, it is necessary to develop pesticide standard values comprehensively by considering all possible human exposure scenarios. The IMDL was applied to examine the pesticide standard values in major exposure pathways by computing the implied maximum pesticide mass burden from all major exposures; this was then compared with the ADI value of the same pesticide. The IMDL could be an indicator to assess whether the pesticide standard values were developed comprehensively and conservatively enough to protect human health in all major exposure scenarios. The IMDL was developed based on human exposure models in the following equations. Since there is little information about the worldwide pesticide air standards, the pesticide exposure from the residential air was omitted.

For drinking water:

(16) $${\mathrm{IDL}}_{\mathrm{dw}}=\left(\frac{\mathrm{EF}}{\mathrm{HW}}\right)\times \mathrm{MCL}\times V$$

For residential soil:

(17) $${\mathrm{IDL}}_{\mathrm{soll}}=\left(\frac{\mathrm{EF}}{\mathrm{HW}}\right)\times \left[\mathrm{RGV}\times \mathrm{CF}\times \mathrm{IR}+\mathrm{RGV}\times \mathrm{CF}\times {\mathrm{ABS}}_{d}\times \mathrm{GIABS}\right]$$

For agricultural commodities:

(18) $${\mathrm{IDL}}_{\mathrm{food}}=\left(\frac{\mathrm{EF}}{\mathrm{HW}}\right)\times \overset{n}{\underset{j=1}{\sum }}({\mathrm{MRL}}_{\mathrm{ij}}\times {\mathrm{FIR}}_{j})$$

(19) $$\mathrm{IMDL}=\left(\frac{\mathrm{EF}}{\mathrm{HW}}\right)\times \left[\mathrm{MCL}\times V+\mathrm{RGV}\times \mathrm{CF}\times \mathrm{IR}+\mathrm{RGV}\times \mathrm{CF}\times {\mathrm{ABS}}_{d}\times \mathrm{GIABS}+\overset{n}{\underset{j=1}{\sum }}({\mathrm{MRL}}_{\mathrm{ij}}\times {\mathrm{FIR}}_{j})\right]$$

where IDL is the implied dose limit (mg/kg-day) computed from drinking water, soil, and agricultural commodities. For the IDL_soil_, since the soil dust inhalation pathway contribution is extremely low compared to soil ingestion and soil dermal contact exposure, the soil inhalation exposure was omitted for the IDL_soil_ calculation. If more than one PSV for a certain pesticide was regulated by a nation in one major exposure, different IMDLs were calculated by combining different IDLs with others.

## 4. Results and Discussion

### 4.1. Numbers of RGVs and MCLs in Worldwide Jurisdictions 

**Figure 1:**
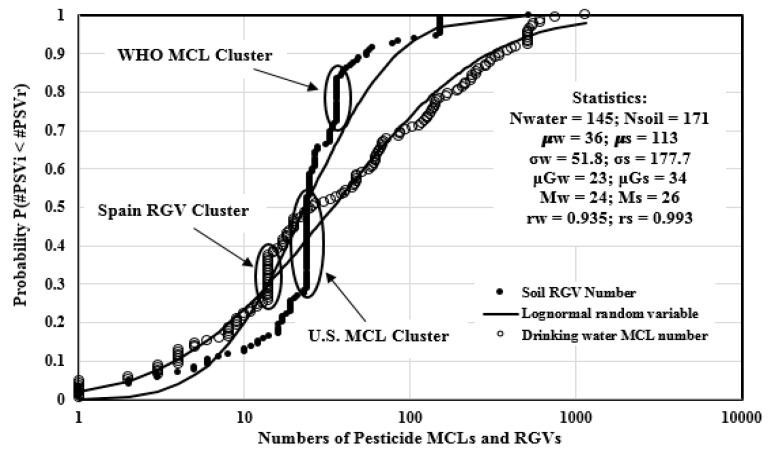
Empirical distributions of the number of worldwide regulatory guidance values (RGVs) and maximum concentration levels (MCLs) compared to the theoretical distribution of a lognormal random variables.

Figure 1 illustrated the distributions of the numbers of pesticide soil RGVs and drinking water MCLs regulated by worldwide jurisdictions. There are 145 worldwide soil jurisdictions and 171 drinking water jurisdictions which had provided the pesticide standard values. For worldwide soil jurisdictions, the numbers of RGVs span 3.06 orders of magnitude (1, 1140), and are well dispersed with the Pearson coefficient of 0.993. The state of Texas had provided the maximum number of soil RGVs, which is 1140. Jurisdictions from Quebec (Canada), three Italian regions, Moscow (Russia), Turkey, Anglian Water Services (United Kingdom), and the Confederated Tribes of the Colville Reservation (U.S.) only regulated one soil pesticide RGV. There is one soil RGV number data cluster at 14. The data cluster is made up of 21 jurisdictions from the European Union, Andorra, China (Beijing), Poland, the state of Connecticut, and 16 Span jurisdictions. The arithmetic mean of the soil RGV numbers is 113, and only 50 (29.2% of the total) jurisdictions had RGV numbers above this value, because the arithmetic mean is heavily skewed by some large values such as 1140 (Texas) at the high end of the distribution. The median and geometric mean of the RGV numbers are 26 and 34, respectively, which are better measures of the central tendency of the distribution. For drinking water jurisdictions, the numbers of the MCLs span 2.72 orders of magnitude (1520), and are well dispersed with the Pearson coefficient of 0.935. Since some nations, such as European nations, applied drinking water MCLs as individual and total standards, the number of the MCLs regulated in these nations is not clear (see Table 1 for details) and the information regarding MCL numbers from these jurisdictions was not shown in Figure 1. The U.S. Army Public Health Command had provided the maximum number of drinking water MCLs, which is 520. Jurisdictions from Morocco, South Africa, Tanzania, Thailand, and Tunisia only regulated one pesticide MCL. There are two drinking water MCL numbers data clusters. The cluster at 24 is made up of 35 values from the province of Ontario (Canada) and 34 U.S. related jurisdictions. The cluster at 36 is made up of 17 values from the WHO, Albania, Antigua and Barbuda, Bahamas, Belize, Bhutan, State of San Paolo (Brazil), Fiji, Japan, Kiribati, Kuwait, Nauru, Sudan, Tonga, Tuvalu, Vanuatu, and Vietnam. The arithmetic mean of the MCL numbers is 36, and this value is larger than the median and geometric mean, which are 24 and 23, respectively, because the arithmetic mean is skewed by some large values such as 520 (U.S. Army Public Health Command). In general, worldwide jurisdictions had regulated more pesticide soil RGVs than drinking water MCLs, because usually pesticides are directly applied to agricultural land, forest, and home gardens, which make the pesticides accumulate in the surface soil first. However, there are more nations regulating pesticides standard values in drinking water (102 nations) than in soil (54 nations), which indicates that more nations focus on the regulation of pesticides in drinking water.

**Figure 2:**
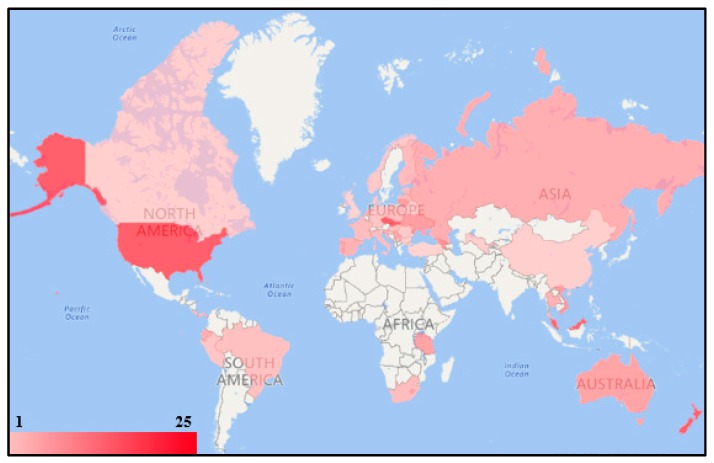
Geographic distribution of the nations regulating the 25 selected pesticides in residential surface soil.

**Figure 3:**
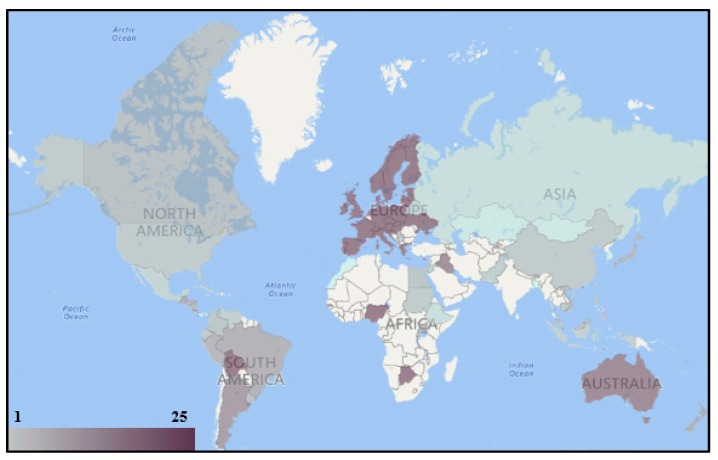
Geographic distribution of the nations regulating the 25 selected pesticides in drinking water.

Since hundreds of different pesticides have been registered for use and nations may apply different pesticides based on their situations, 25 pesticides were generally selected to examine if the worldwide nations had provided enough pesticide regulation information based on historical and current usage. These 25 selected pesticides include 14 current widely used pesticides: 2,4-D, Aldicarb, Atrazine, Chlorothalonil, Chlorpyriphos, Diazinon, Dicamba, Diuron, Glyphosate, Malathion, Mancozeb, MCPA, Metolachlor, and Trifluralin, and 11 historically largely used pesticides (the Stockholm Convention POP): Aldrin, Chlordane, DDT, Dieldrin, Endrin, Heptachlor, Toxaphene, Lindane, Endosulfan, Pentachlorophenol, and Bromomethane. Figure 2 illustrates the geographic distribution of nations on regulating the 25 selected pesticides in residential surface soil. A total of 49 nations have regulated the soil RGV for at least one of these 25 pesticides. Only national jurisdictions were applied, and, if a nation had more than one national jurisdiction, the better performing one was selected. For example, the U.S. EPA regulated the RGVs for all of these 25 pesticides, and the U.S. ASTDR provided the RGVs for only eight of the selected pesticides. Therefore, the U.S. EPA was selected here as the U.S. national representative jurisdiction. In Figure 2, a nation with a darker red color means that this nation had regulated more selected pesticides for soil. The Czech Republic, New Zealand, Slovakia, and the U.S. regulated soil RGVs for all the selected pesticides. Malaysia provided the RGV for 24 selected pesticides and the Bahamas regulated for 20 pesticides. The arithmetic mean for the number of selected pesticides which had been regulated with the soil RGV is 9. Some nations in Africa, Asia, and South America did not provide any soil pesticide standard values for the selected pesticides. Some multinational organizations, including both the EU and WHO, regulated the soil RGVs for eight of the selected pesticides. Figure 3 illustrates the geographic distribution of nations regulating the 25 selected pesticides in drinking water. There are 97 nations which provided the pesticide drinking water MCL for at least one of the 25 selected pesticides. Most of the European nations had regulated the MCLs for all selected pesticides, because these nations applied EU standards, which provided individual and total standards for any pesticide. Australia provided the MCLs for 22 selected pesticides and Iraq regulated for 21. Both the U.S. and China regulated the MCLs for 9 of the 25 selected pesticides, while Bangladesh, South Korea, and Morocco had only provided the MCLs for one of the selected pesticides. Some nations in Africa and Asia did not provide any drinking water pesticide standard values for the selected pesticides. The arithmetic mean for the number of selected pesticides which had been regulated with the drinking water MCL is 16. In terms of the multinational organizations, the EU provided all MCLs for these 25 pesticides and the WHO regulated for 13.

### 4.2. The Most Commonly Regulated Pesticides in Soil and Drinking Water

The most commonly regulated pesticides in this study were defined as pesticides with over 100 soil RGVs or drinking water MCLs. Table 4 summarizes the commonly regulated pesticides by the common name under which they were most often regulated in jurisdictions, CAS No, occurrence frequency (in U.S. jurisdictions and worldwide jurisdictions), the lowest and highest values, and the log orders of variation (LOV, LOV = log {highest value/lowest value}) over which the RGVs and MCLs were scattered. There are 39 most commonly regulated pesticides with either soil RGVs or drinking water MCLs regulated above 100. DDT is the most frequently regulated pesticide in soil with 319 RGVs, made up of 140 RGVs from the U.S. related jurisdictions and 179 RGVs from the jurisdictions outside of the U.S., while 2,4-D is the most frequently regulated pesticides in drinking water with 180 MCLs, including 59 U.S. MCLs and 121 worldwide MCLs. Pesticides including Endosulfan, α-HCH, β-HCH, Bromomethane, and o-Cresol were regulated by over 100 RGVs in soil but less than 13 MCLs in drinking water. There were 125 drinking water MCLs regulated for DBCP, while 31 DBCP soil RGVs were promulgated with only one RGV from the jurisdictions outside of the U.S. There were 22 and 25 pesticides which had been regulated with over 100 soil RGVs and drinking water MCLs, respectively. For these 39 most commonly regulated pesticides, the state of Idaho had specified the lowest soil RGVs for at least 7 pesticides, and both Oregon and Serbia provided the lowest RGVs for at least four of the most commonly regulated pesticides. Texas specified the highest soil RGVs for at least 14 of the commonly regulated pesticides, Guam (U.S.) provided the highest RGVs for five pesticides, and the U.S. Military jurisdiction regulated for four pesticides. Regardinf drinking water, the EU and the jurisdictions which applied the EU standards specified the lowest drinking water MCLs for at least 21 of the most commonly regulated pesticides, and Wyoming provided the lowest MCLs for at least 9 pesticides. The EU provided the pesticide drinking water MCLs quite conservatively, because the jurisdiction specified 0.0001 mg/L for an individual pesticide. On the other hand, the U.S. Military jurisdiction promulgated the highest MCLs for at least 12 pesticides; this is probably because the jurisdiction derived the MCL based on adult body weight and short time exposure conditions. Vietnam regulated the highest MCLs for 9 of the commonly regulated pesticides, and Mexico provided the highest MCLs for 5.

**Figure 4:**
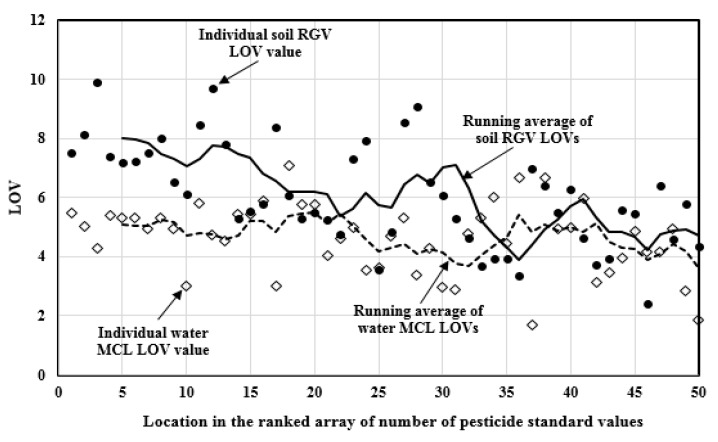
Log orders of variation (LOV) values and the running average for the commonly regulated pesticides in soil and drinking water.

The LOV values for the 50 most commonly regulated pesticides in soil range from 2.41 (Terbufos) to 9.89 (Dieldrin). The arithmetic mean of these LOV values is 6.08. The average LOV value for the 22 most commonly regulated pesticides in soil is 6.97, and the average for the rest of the pesticides is 5.38. The LOV values for the 50 most commonly regulated pesticides in drinking water range from 1.70 (Epichlorohydrin) to 7.11 (DDT). The arithmetic mean of the drinking water pesticides LOV values is 4.63. For the 25 most commonly regulated pesticides in drinking water, the average LOV value is 4.93, and the average LOV value for the rest is 4.33. Figure 4 illustrates the individual and running average LOV values for the 50 most commonly regulated pesticides in soil and drinking water. In general, the individual and running average LOV values are smaller when the number of RGVs and MCLs decreases, and the LOV values of pesticides drinking water MCLs are less than soil RGVs.

### 4.3. Analysis of Soil RGVs for the Commonly Regulated Pesticides

**Figure 5:**
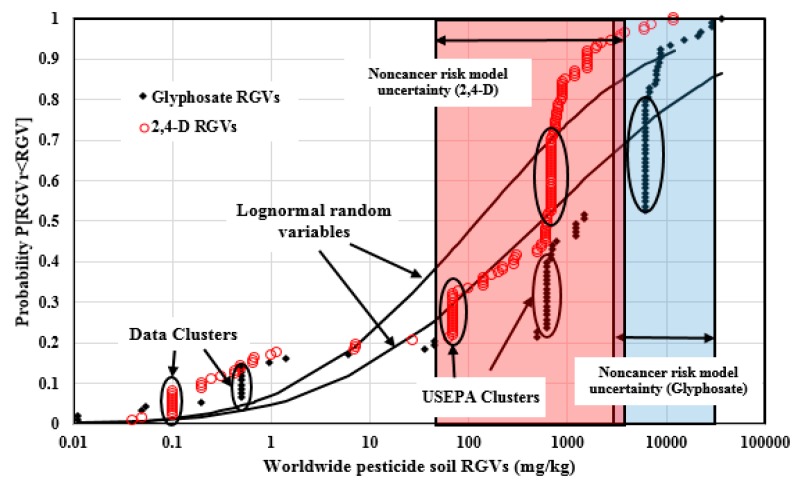
2,4-D and Glyphosate soil RGVs plotted in the empirical cumulative distribution forms and compared to the log-normal random variable cumulative distributions with identical statistics.

2,4-D and Glyphosate are two most widely used pesticides today and are two commonly regulated pesticides in residential surface soil. Figure 5 illustrates the 147 2,4-D and 93 Glyphosate soil RGVs which were plotted in the empirical cumulative distribution forms and compared to the logn-ormal random variable cumulative distributions with the identical statistics. There were 103 2,4-D and 73 Glyphosate RGVs from the U.S. jurisdictions; the rest were from other nations around the world. The 2,4-D RGVs span 5.78 orders of magnitude (0.04, 12000.0) mg/kg, and are well dispersed with the Pearson coefficient of 0.907. The Glyphosate RGVs span 6.51 orders of magnitude (0.011, 36,000) mg/kg, and are also well dispersed with the Pearson coefficient of 0.904. Those correlations were influenced by several RGV data clusters. The 2,4-D soil RGV data set has three non-random data clusters at 0.1 mg/kg (10 values), 69 mg/kg (16 values), and 690 mg/kg (23 values). For Glyphosate, there are also three non-random data clusters at 0.5 mg/kg (8 values), 610 mg/kg (16 values), and 6090ߝ6110 mg/kg (24 values).

The cancer risk uncertainty bounds for 2,4-D and Glyphosate were not derived, because the U.S. EPA did not consider the cancer risk for 2,4-D and Glyphosate RGV calculations. The non-cancer risk uncertainty bounds derived for 2,4-D soil RGVs were (45.0, 3690.0) mg/kg. Six values (4.1% of the total RGVs) are above the non-cancer uncertainty upper bound, 111 values (75.5%) fall within the uncertainty bounds, and 30 values (20.4%) are below the lower non-cancer risk uncertainty bound. For Glyphosate, the computed non-cancer risk uncertainty bounds were (3000.0, 32,000.0) mg/kg. Only one RGV is above the uncertainty upper bound, 44 values (47.3% of the total RGVs) fall within the bounds, and 48 values (51.6%) are below the lower bound.

The arithmetic mean of the 2,4-D RGVs is 867.0 mg/kg, which is exceeded by only 30 RGVs. This value is heavily affected by some large RGVs, such as 12,000.0 mg/kg (Oregon) at the high end of the data distribution. The geometric mean and median are 122.0 mg/kg and 630.0 mg/kg, respectively, which are better measures of the central tendency of the distribution. For Glyphosate, the arithmetic mean of the RGVs is 4715.7 mg/kg, with 45 RGVs larger than this value; this is because it is heavily affected by large RGVs, such as 36,000.0 mg/kg (Texas). The geometric mean and median are 545.8 mg/kg and 1500.0 mg/kg, respectively, which are better measures of the central tendency of the distribution.

Table 5 provides the statistical summary information of the RGVs for the 27 commonly regulated pesticides. The weighted average of the Pearson coefficients by the RGV number is 0.958, and nearly all the pesticides RGVs have Pearson coefficients above 0.900, except Picloram (0.878), which indicates that the soil RGVs for these commonly regulated pesticides are well dispersed. Some pesticides, such as Chlordane, Lindane, and Atrazine, have coefficients very close to 1.00. For these pesticides, there are at least 1632 RGVs (42% of the total) in the non-random data clusters, and the data clusters are usually made up of the jurisdictions from U.S., EU, Spain, Canada, and Australia, which means that the national jurisdictions in these nations had provided leadership in soil pesticide regulations. There are at least 300 soil RGVs (8% of the total) above all of the computed human health uncertainty upper bounds, which indicates that, for these 27 commonly regulated pesticides in soil, at least 300 RGVs cannot protect human health.

### 4.4. Analysis of Drinking Water MCLs for the Commonly Regulated Pesticides

**Figure 6:**
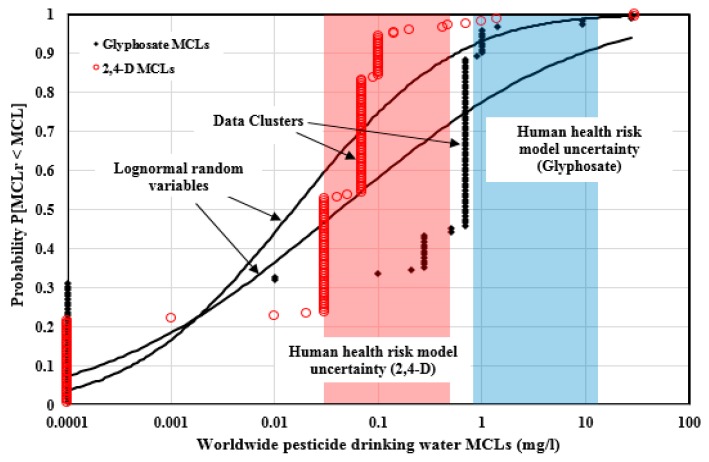
2,4-D and Glyphosate drinking water MCLs plotted in the empirical cumulative distribution forms and compared to the log-normal random variable cumulative distributions with identical statistics.

Figure 6 illustrates the 180 2,4-D and 122 Glyphosate drinking water MCLs which were plotted in the empirical cumulative distribution forms and compared to the log-normal random variable cumulative distributions with identical statistics. There were 59 2,4-D and 56 Glyphosate MCLs from the U.S. jurisdictions; the rest were from other nations around the world. The 2,4-D MCLs span 5.48 orders of magnitude (0.0001, 30.0) mg/L, and are well dispersed over this span with the Pearson coefficient of 0.861. The Glyphosate MCLs span 5.45 orders of magnitude (0.0001, 28.0) mg/L, and are also well dispersed with the Pearson coefficient of 0.864. Those correlations were influenced by several MCL data clusters. The 2,4-D drinking water data set has four non-random data clusters at 0.1 mg/L (19 values), 0.07 mg/L (53 values), 0.03 mg/L (53 values), and 0.0001 mg/L (39 values). For Glyphosate, there are two non-random data clusters at 0.7 mg/L (53 values) and 0.0001 mg/L (38 values).

The human health risk uncertainty bounds derived for 2,4-D drinking water MCLs were (0.03, 0.47) mg/L. Six values (3.3% of the total MCLs) are above the uncertainty upper bound, 132 values (73.3%) fall within the uncertainty bounds, and 42 values (23.3%) are below the lower risk uncertainty bound. For Glyphosate, the computed human health risk uncertainty bounds were (0.9, 14.0) mg/L. Only two MCLs are above the uncertainty upper bound, 12 values (9.83% of the total MCLs) fall within the bounds, and 108 values (88.5%) are below the lower bound.

The arithmetic mean of the 2,4-D MCLs is 0.40 mg/L, which is exceeded by only eight MCLs. This value is heavily affected by some large MCLs, such as 30 mg/L (Mexico and Vietnam) at the high end of the data distribution. The geometric mean and median are 0.015 mg/L and 0.03 mg/L, respectively, which are better measures of the central tendency of the distribution. For Glyphosate, the arithmetic mean of the MCLs is 1.04 mg/L, with only five MCLs larger than this value; this is because it is heavily affected by large MCLs such as 20 mg/L (U.S. Military). The geometric mean and median are 0.042 mg/L and 0.7 mg/L respectively, which are better measures of the central tendency of the distribution.

Table 6 provides the statistical summary information of the MCLs for the 29 commonly regulated pesticides. The weighted average of the Pearson coefficients by the MCL number is 0.904, and nearly all the pesticides MCLs have Pearson coefficients above 0.850, except Aldrin (0.817), Chlorpyrifos (0.811), and Dieldrin (0.803), which indicates that the drinking water MCLs for these 29 commonly regulated pesticides are well dispersed. Some pesticides, such as Heptachlor and Pentachlorophenol, have coefficients above 0.950. For these 29 pesticides, there are at least 2980 MCLs (80.7% of the total) in the non-random data clusters, and the data clusters are usually made up of the jurisdictions from U.S., EU, and WHO, which means that these national and multination jurisdictions had provided leadership in drinking water pesticide regulations. There are at least 172 drinking water MCLs (5% of the total) above the computed human health uncertainty upper bounds, which indicates that, for these 29 commonly regulated pesticides in drinking water, at least 172 MCLs cannot protect human health.

### 4.5. Analysis of the IEDs for the Widely Used Pesticides in Agricultural Commodities

**Figure 7:**
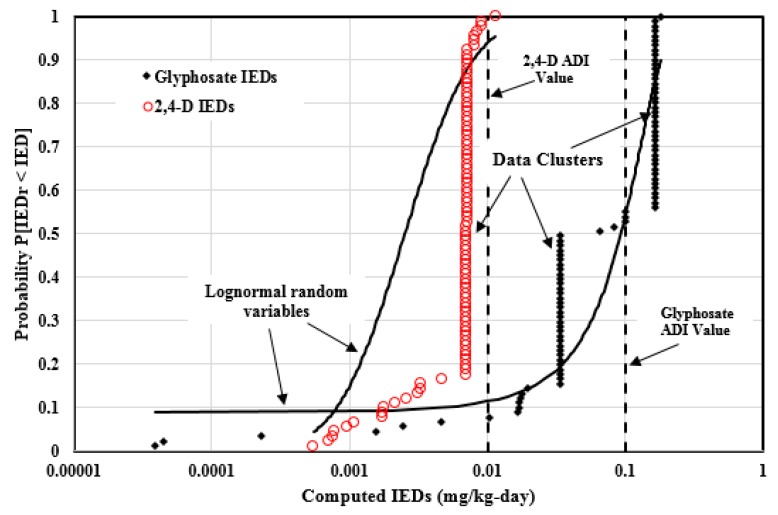
2,4-D and Glyphosate agricultural commodities implied exposure doses (IEDs) plotted in the empirical cumulative distribution forms and compared to the log-normal random variable cumulative distributions with identical statistics.

Figure 7 illustrates the 91 2,4-D and 91 Glyphosate agricultural commodities’ IEDs which were plotted in the empirical cumulative distribution forms and compared to the log-normal random variable cumulative distributions with identical statistics. The 2,4-D IEDs span 1.33 orders of magnitude (0.00054, 0.011) mg/kg-day with the Pearson coefficient of 0.761. For Glyphosate, the IEDs span 3.67 orders of magnitude (0.0000386, 0.18) mg/kg-day with the Pearson coefficient of 0.907. Those correlations were heavily influenced by several large IED data clusters. The 2,4-D food IED set has two large non-random data clusters at 0.0072 mg/kg-day (32 values) and 0.0069 mg/kg-day (31 values). For Glyphosate, there are also two large non-random data clusters at 0.17 mg/kg-day (31 values) and 0.033 mg/kg-day (33 values).

The ADI value of 2,4-D is 0.01 mg/kg-day. Only one IED computed from Russia is above the ADI value. Although most IEDs computed from the most commonly consumed agricultural commodities are within the safety levels (i.e. lower than the 2,4-D ADI value), this does not account for other foods (meat or dairy product) and other exposure pathways. For example, the 2,4-D IED computed from the U.S. and Mexico is 0.009 mg/kg-day, which is nearly 90% of the 2,4-D ADI value, which indicates that there is little safety margin left for other foods or exposure pathways. For Glyphosate, 41 IEDs computed from the most commonly consumed agricultural commodities exceed the ADI value of 0.1 mg/kg-day, and this does not account for other foods or exposure pathways either. Most nations only regulated the MRLs for four of the 12 most commonly consumed agricultural commodities (see Table 3).

Table 7 provides the statistical summary information of the IEDs for the 14 widely used pesticides. The weighted average of the Pearson coefficients by the IED number is 0.825. Some pesticides, such as Diazinon, Malathion, and Mancozeb, have Pearson coefficients around 0.950, while some other pesticides, such as Dicamba and MCPA, have coefficients less than 0.700, because the correlations are heavily affected by large IED data clusters. For these 14 pesticides, there are at least 804 IEDs (72.7% of the total) in the non-random data clusters, and the data clusters are usually led by EU and WHO jurisdictions. There are at least 310 computed IEDs (28.0% of the total) above the ADI values, which indicates that, for these 14 widely used pesticides, at least 310 IEDs computed from the agricultural commodities MRLs cannot protect human health.

### 4.6. Analysis of the IMDLs for the Widely Used Pesticides

**Figure 8:**
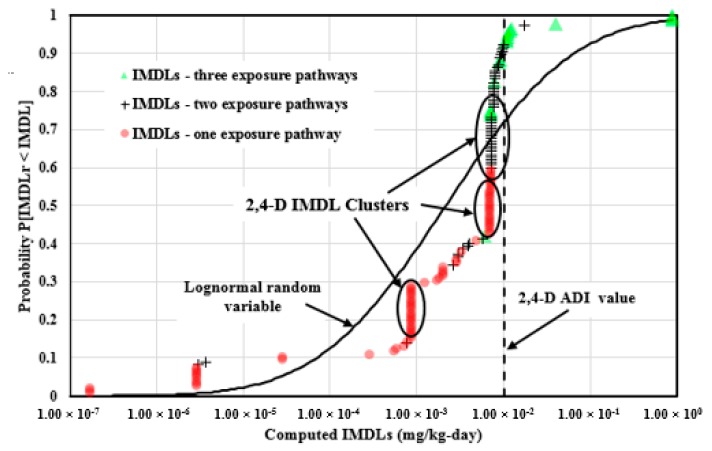
2,4-D implied maximum dose limits (IMDLs) computed from soil, drinking water, and the commonly consumed agricultural commodities plotted in the empirical cumulative distribution form and compared to the log-normal random variable cumulative distribution with identical statistics.

Figure 8 illustrates the 145 2,4-D IMDLs computed from three major exposure pathways which were plotted in the empirical cumulative distribution form and compared to the log-normal random variable cumulative distribution with identical statistics. A total of 17 IMDLs were computed from three exposure pathways, 53 values were calculated from two of the major exposure pathways, and 75 values were computed from one of the major exposure pathways. The 2,4-D IMDLs span 6.70 orders of magnitude (1.73 × 10^−7^, 8.66 × 10^−1^) mg/kg-day, and are well dispersed over this span with the Pearson coefficient of 0.881. The correlation was heavily influenced by three IMDL data clusters at 0.00718–0.00781 mg/kg-day (33 values), 0.00695 mg/kg-day (17 values), and 0.000857 mg/kg-day (21 values).

The maximum IMDL value is 0.866 mg/kg-day, computed from the Vietnam national drinking water MCL, agricultural commodity MRLs, and soil RGV (2008). The minimum IMDL value is 1.73 × 10^−7^ mg/kg-day, computed from the Armenia, Georgia, and Moldova soil jurisdictions. A total of 13 IMDLs (9.0% of the total number) are above the 2,4-D ADI value, 10 of the IMDLs were computed from three major exposure pathways, and three of them were computed from two of the major exposure pathways. Although 91% of the 2,4-D IMDLs computed from the national jurisdictions are below the ADI value, 95.5% of the IMDLs were computed from either two of the major exposure pathways or one exposure pathway, which indicates that many nations did not provide enough information to protect human health against 2,4-D exposure.

**Figure 9:**
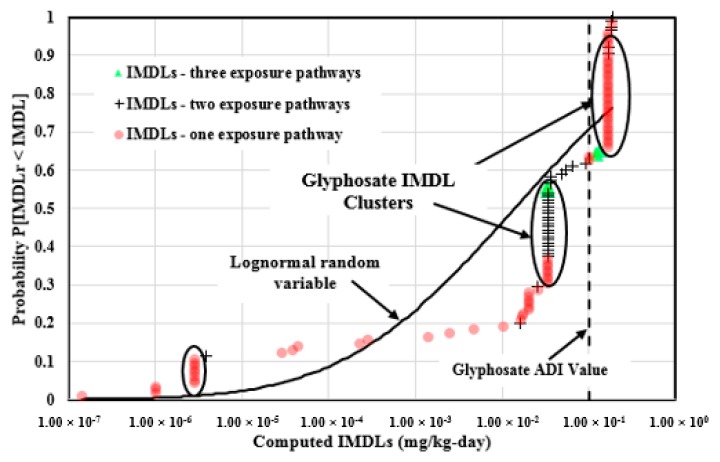
Glyphosate IMDLs computed from soil, drinking water, and the commonly consumed agricultural commodities plotted in the empirical cumulative distribution form and compared to the log-normal random variable cumulative distribution with identical statistics.

Figure 9 illustrates the 115 Glyphosate IMDLs computed from three major exposure pathways which were plotted in the empirical cumulative distribution form and compared to the log-normal random variable cumulative distribution with identical statistics. Four IMDLs were computed from three exposure pathways, 36 values were calculated from two of the major exposure pathways, and 75 values were computed from one of the major exposure pathways. The Glyphosate IMDLs span 6.11 orders of magnitude (1.43 × 10^−7^, 1.86× 10^−1^), and are well dispersed over this span with the Pearson coefficient of 0.854. The correlation was heavily influenced by three IMDL data clusters at 0.166 mg/kg-day (35 values), 0.0335 mg/kg-day (30 values), and 2.86 × 10^−6^ mg/kg-day (8 values).

The maximum IMDL value is 0.186 mg/kg-day, computed from the Guatemala national drinking water MCL and food MRL. The minimum IMDL value is 1.43 × 10^−7^ mg/kg-day, computed from the Iraq drinking water MCL only. A total of 42 (27.1% of the total) IMDLs are above the Glyphosate ADI value. Although 72.9% of the Glyphosate IMDLs computed from the national jurisdictions are below the ADI value, most of the IMDLs were computed from either two of the major exposure pathways or one exposure pathway, which indicates that many nations did not provide enough information to protect human health against Glyphosate exposure.

Table 8 provides the statistical summary information of the IMDLs for the 14 widely used pesticides. The weighted average of the Pearson coefficients by the IMDL number is 0.868. Aldicarb has a Pearson coefficient of 0.977, while Malathion has the low coefficient of 0.688, because the correlation is heavily affected by large IMDL data clusters. For these 14 pesticides, there are at least 931 IEDs (60.3 % of the total) in the non-random data clusters, and the data clusters are usually led by EU and WHO jurisdictions. There are at least 373 computed IMDLs (24.1% of the total) above the ADI values, which indicates that, for these 14 widely used pesticides, at least 373 IMDLs computed from the national jurisdictions cannot protect human health.

## 5. Conclusions

Over 145 worldwide soil jurisdictions from 54 nations (27.7% of the total nations) and 171 drinking water jurisdictions from 102 nations (52.3%) had provided the pesticide standard values. Over 90 nations (46.2%) had regulated the pesticide agricultural commodities MRLs which were collected in the global MRL database (2014) [16]. Only the U.S. had systematically promulgated the pesticide air MCLs. This research shows that many nations are lacking pesticide standard values for the major exposure pathways, especially nations in Africa, Asia, and South America. For nations which provided the pesticide standard values, human health risk uncertainty bounds and the implied total exposure mass burden method were introduced to examine whether current standard values in the major exposure pathways can protect human health. The results indicate that some pesticide standard values were derived too high to protect human health, even in a single exposure pathway, such as soil. This is because some jurisdictions derived the pesticide standard values without considering other exposure probabilities, did not derive them comprehensively and conservatively enough for human health risk control. In addition, the standard values for most commonly regulated pesticides often vary over seven, eight, or nine orders of magnitude, indicating that there is little agreement on the regulation of pesticide standard values among worldwide jurisdictions. This study will help worldwide pesticide regulatory jurisdictions to rationalize their standard values and provide some references for nations which do not yet have pesticide standard values.

## Figures and Tables

**Table 1:** Worldwide (outside of U.S.) pesticide regulatory guidance values (RGVs) and maximum concentration levels (MCLs) jurisdiction sources for nations, regions, territories, and multi-national organizations.

No.	Worldwide Jurisdictions	No. of Soil RGVs	No. of Water MCLs	Sources of Pesticide Soil RGVs ^1^	Language (Soil RGVs)	Sources of Pesticide Water MCLs ^1^	Language (Water MCLs)
*Multinational organizations*							
1	East Africa Community	17	19	East Africa Community (2011)	English	East African Community (2012)	English
2	European Union	14	UNK ^2^	European Union (2010)	English	European Union (1998)	English
3	Gulf Standardization Organization	--- ^3^	33	---	---	Gulf Standardization Organization (2012)	Arabic & English
4	World Health Organization	11	36	World Health Organization (2002)	English	World Health Organization (2011)	English
*United Nations member states*							
1	Republic of Albania	---	36	---	---	Albania Institute for European Environmental Policy (2007)	Albanian
2	Principality of Andorra	14	25	Andorra Official Gazette (2010)	Catalan	Andorra Official Gazette (1999)	Catalan
3	Antigua and Barbuda	---	36	---	---	Environmental Solutions Antigua Limited (2008)	English
4	Argentine Republic	---	49	---	---	Argentine Official Gazette (1993)	Spanish
5	Republic of Armenia	286	---	Armenia Minister of Health (2011)	Armenian	---	
6	Commonwealth of Australia	48	152	Australia National Environmental Protection Council (1999)	English	National Health and Medical Research Council (2013)	English
	(Australia) Australian Capital Territory	48	152	Australia National Environmental Protection Council (1999)	English	Australian Capital Territory Ministry of Health (2007)	English
	(Australia) Tasmania	62	152	Tasmania Environmental Protection Authority (2012)	English	Tasmania Department of Health and Human Services (1997)	English
				Australia National Environmental Protection Council (1999)	English		
	(Australia) New South Wales	18	152	New South Wales Department of Environment and Conservation (2006)	English	Australia Department of Health (2014)	English
	(Australia) Northern Australia	48	152	Australia National Environmental Protection Council (1999)	English	Australia Department of Health (2014)	English
	(Australia) Queensland	48	145	Australia National Environmental Protection Council (1999)	English	Government of Queensland (2014)	English
	(Australia) State of Victoria	67	---	Victoria Environmental Protection Authority (2002)	English	---	---
				Australia National Environmental Protection Council (1999)	English		
	(Australia) South Australia	66	152	Australia National Environmental Protection Council (1999)	English	Government of South Australia (2011)	English
				South Australia Environment Protection Authority (2006)	English		
	(Australia) Western Australia	18	152	Western Australia Department of Environment and Conservation (2010)	English	Australia Department of Health (2014)	English
7	Republic of Austria	---	UNK	---	---	Austria Department of Health (2013)	German
8	Commonwealth of the Bahamas	123	36	Bahamas Ministry of Works and Transport (2008)	English	The Bahamas Water and Sewerage Corporation (1999)	English
9	People’s Republic of Bangladesh		2			Amio Water Treatment Limited (2010)	English
10	Republic of Belarus	139	16	Belarus Ministry of Health (2004)	Belarusian	Belarus Ministry of Health (2013)	Russian
11	Belize	---	36	---	---	Belize Agricultural Health Authority (2003)	English
12	Kingdom of Bhutan	---	36	---	---	Codex Alimentarius (2001)	English
13	Plurinational State of Bolivia	---	UNK	---	---	Bolivia Ministry of Public Works and Services Vice of Basic Services (2004)	Spanish
14	Republic of Botswana	---	UNK	---	---	Water Utilities Corporation (2000)	English
15	Federative Republic of Brazil	8	26	Brazil Ministry of the Environment (2009)	Portuguese	Brazil Ministry of Health (2004)	Portuguese
	(Brazil) State of San Paolo	8	36	Environmental Company of Sao Paolo (2005)	Portuguese	Government of State of San Paolo (2008)	Portuguese
16	Republic of Bulgaria	64	UNK	Bulgaria Ministry of Environment and Water (2008)	Bulgarian	Bulgaria Ministry of Health (2001)	Bulgarian
17	Kingdom of Cambodia	---	19	---	---	Cambodia Ministry of Industry Mines and Energy (2004)	English
18	Canada	4	25	Canadian Council of Ministers of the Environment (CCME) (2011)	English	Health Canada (2012)	English
	(Canada) Alberta	86	25	Alberta Environment (2010)	English	Alberta Health Services (2013)	English
	(Canada) British Columbia	206	25	---	---	British Columbia Ministry of Health (undated)	English
				British Columbia Regulations (2013)	English		
				British Columbia Regulations (2013)	English		
	(Canada) Province of Manitoba	4	---	Manitoba Conservation (2011), CCME (2011)	English	---	---
	(Canada) Newfoundland and Labrador	3	25	Environment and Conservation, Government of Newfoundland and Labrador (2005)	English	Government of Newfoundland and Labrador (2013)	English
	(Canada) Northwest Territories	4	25	Northwest Territories Department of Environment and Natural Resources (2003)	English	Canada Northwest Territories Municipal and Community Affairs (undated)	English
	(Canada) Nova Scotia	169	57	Nova Scotia Environment (2013)	English	Government of Nova Scotia (2012), Nova Scotia Environment and Labor (undated)	English
	(Canada) Nunavut	6	25	Department of Environment, Government of Nunavut (2009)	English	National Collaborating Centre for Environmental Health (2014)	English
	(Canada) Ontario	72	24	Ontario Ministry of the Environment (2011)	English	Canadian Institute for Environmental Law and Policy (2003)	English
	(Canada) Prince Edward Island	3	25	Prince Edward Island Environment, Energy and Forestry (2010)	English	Prince Edward Island Department of Environment, Labor and Justice (2012)	English
	(Canada) Quebec	1	34	Quebec Ministry of Sustainable Development, Environment and Parks (1998)	French	Government of Quebec (2014)	English
	(Canada) Saskatchewan	---	12	---	---	Saskatchewan Environment (2006)	English
	(Canada) Yukon	5	25	Yukon Regulations (2002)	English	Government of Yukon (2007)	English & French
19	Republic of Chile	---	8	---	---	Chile Ministry of Public Works (2005)	Spanish
20	People’s Republic of China	20	17	Peoples Republic of China (1995)	Chinese	China Department of Health (2007)	Chinese
				People’s Republic of China Ministry of Environmental Protection (2006)	Chinese		
	China (Beijing)	14	---	Beijing Municipal Environmental Protection Bureau (2011)	Chinese	---	---
21	Republic of Colombia	---	16	---	---	Colombian Institute for Technical Standards and Certification (1994)	Spanish
22	Republic of Costa Rica	8	33	Ministry of Health (2011)	Spanish	Costa Rica Minister of Finance (2005)	Spanish
23	Republic of Croatia	15	UNK	Agricultural University of Zagreb (2008)	Croatian	Croatia Ministry of Health and Social Welfare (2007)	Croatian
24	Republic of Cuba	---	16	---	---	Cuba Government (1997)	Spanish
25	Republic of Cyprus	---	UNK	---	---	Cyprus Ministry of Agriculture, Natural Resources and Environment (1999)	English
26	Czech Republic	11	UNK	Czech Republic Ministry of the Environment (1994)	English	European Commission (1998)	Czech
				European Commission (2007)	English		
27	Kingdom of Denmark	9	UNK	Danish Environmental Protection Agency (2010)	Danish	Nature Agency of Denmark (2014)	Danish
28	Dominican Republic	---	UNK	---	---	Dominican Ministry of Public Health and Social Assistance (2005)	Spanish
29	Republic of Ecuador	27	19	Ecuador Ministry of the Environment (2004)	Spanish	Ecuadorian Institute of Standards (2011)	Spanish
30	Arab Republic of Egypt	---	33	---	---	World Health Organization Regional Office for the Eastern Mediterranean (2006)	English
31	Republic of Estonia	12	UNK	Estonia Ministry of the Environment (2004)	Estonian	Estonia Minister of Social Affairs (2013)	Estonian
32	Federal Democratic Republic of Ethiopia	---	10	---	---	World Health Organization (2010)	English
33	Republic of Fiji	---	36	---	---	Secretariat of the Pacific Community (2005)	English
34	Republic of Finland	12	UNK	Finland Ministry of the Environment (2007)	Finish	Finland Minister of Social Affairs and Health (2001)	Finish
35	French Republic	18	UNK	European Commission (2007)	English	France Ministry of Ecology, Sustainable Development And Energy (1998)	French
36	Republic of the Gambia	---	UNK	---	---	Gambia Environmental Quality Standards Board (1999)	English
37	Georgia	231	UNK	Georgia Minister of Environment and Minister of Natural Resources (2006)	Georgian	Georgia Ministry of Justice (2007)	Georgian
				Minister of Health, labor and social affairs (2001)	Georgian		
38	Federal Republic of Germany	8	UNK	German Federal Ministry of the Environment, Nature Conservation and Nuclear Safety (1999)	German	Germany Federal Ministry of Justice and Consumer Protection (2001)	German
40	Republic of Guatemala	---	55	---	---	Guatemala Government (1999)	Spanish
39	Hellenic Republic	---	UNK	---	---	Greece Central Public Health Laboratory (1998)	Greek
41	Republic of Honduras	---	33	---	---	Honduras Department of Health (1995)	Spanish
42	Republic of Hungary	68	UNK	Hungary Ministry of the Environment (2000)	Hungarian	Hungary National Public Health and Medical Officer Service (2001)	Hungarian
43	Republic of Indonesia	---	17	---	---	Indonesia Government (1990)	Indonesian
44	Republic of Iraq	---	3	---	---	Iraq Central Agency for Meteorology and Quality Control (2001)	Arabic & English
45	Ireland	---	UNK	---	---	Ireland EPA (2007)	English
46	State of Israel	---	7	---	---	Israel Ministry of Health (2000)	Hebrew
47	Republic of Italy	13	59	Italy National Institute of Health (2006)	Italian	Navy Medicine (2012)	English
			UNK			Italy Ministry of Health (2001)	Italian
	(Italy) Lombardi Region	9	---	Tazzioli (1999)	Italian	---	---
	(Italy) Piedmont Region	1	---	Tazzioli (1999)	Italian	---	---
	(Italy) Emili Romana Region	1	---	Tazzioli (1999)	Italian	---	---
	(Italy) Liguria Region	1	---	Tazzioli (1999)	Italian	---	---
48	Japan	---	36	---	---	Japan Ministry of Health, Labor and Welfare (2001)	English & Japanese
49	Hashemite Kingdom of Jordan	---	11	---	---	The Jordanian Institute of Standards and Metrology (2001)	English
50	Republic of Kazakhstan	---	3	---	---	Kazakhstan Government (2001)	Russian
51	Republic of Kiribati	---	36	---	---	Secretariat of the Pacific Community (2005)	English
52	Republic of Korea	---	5	---	---	Korea Ministry of Environment (2011)	English
53	State of Kuwait	---	36	---	---	World Health Organization Regional Office for the Eastern Mediterranean (2006)	English
54	Republic of Latvia	17	UNK	Latvia Cabinet of Ministers (2005)	Latvian	Latvia Ministry of Health (2004)	Latvian
55	Lebanese Republic	---	4	---	---	World Health Organization Regional Office for the Eastern Mediterranean (2006)	English
56	Principality of Liechtenstein	---	UNK	---	---	Liechtenstein Drinking Water Inspectorate (1999)	English
57	Republic of Lithuania	24	UNK	Lithuania Ministry of the Environment (2008)	Lithuanian	Lithuania Ministry of Health (2003)	Lithuanian
58	Grand Duchy of Luxembourg	---	UNK	---	---	Luxembourg Collection of Legislation (2002)	French
59	Malaysia	194	23	Malaysia Environment Protection Department (2009)	English	Malaysia Ministry of Health (2010)	English
60	Republic of Malta	---	UNK	---	---	Malta Government (2009)	Maltese
61	Republic of Mauritius	---	10	---	---	Mauritius Government Gazette (1996)	English
62	United Mexican States	---	18	---	---	Government of Mexico (1994)	Spanish
63	Republic of Moldova	166	---	Moldova Ministry of Ecology and Natural Resources (2004)	Romanian	---	---
64	Mongolia	---	5	---	---	Government of Mongolia (2005)	Mongolian
65	Kingdom of Morocco	---	1	---	---	World Health Organization Regional Office for the Eastern Mediterranean (2006)	English
66	Republic of Nauru	---	36	---	---	Secretariat of the Pacific Community (2005)	English
67	Kingdom of the Netherlands	61	UNK	Netherlands National Institute for Public Health and the Environment (2006)	English	Government of Netherlands (2014)	Dutch
				Netherlands Ministry of Economic Affairs, Agriculture and Innovation (2006)	English		
				Netherlands National Institute for Public Health and the Environment (2009)	English		
68	New Zealand	344	55	New Zealand Ministry of the Environment (2012)	English	New Zealand Ministry of Health (2008)	English
				New Zealand Ministry of the Environment	English		
				New Zealand Ministry of the Environment (1997)	English		
				New Zealand Ministry of the Environment (2006)	English		
				New Zealand Ministry of the Environment (2011)	English		
				New Zealand Ministry of the Environment	English		
	(New Zealand) Auckland City Council	9	---	Cavanagh (2006)	English	---	---
	(New Zealand) Auckland Regional Council	5	---	Cavanagh (2006)	English	---	---
	(New Zealand) Bay of Plenty	4	---	Cavanagh (2006)	English	---	---
	(New Zealand) Hastings District Council	3	---	Cavanagh (2006)	English	---	---
	(New Zealand) Tasmasn District Council	10	---	Cavanagh (2006)	English	---	---
	(New Zealand) Waikato Region	8	---	Cavanagh (2006)	English	---	---
69	Republic of Nicaragua	---	35	---	---	Nicaragua Ministry of Health (1994)	Spanish
70	Federal Republic of Nigeria	---	UNK	---	---	Standards Organization of Nigeria (2007)	English
71	Kingdom of Norway	3	UNK	Norwegian Pollution Control Authority (1999)	English	Norway Ministry of Health and Care Services (2001)	Norwegian
72	Islamic Republic of Pakistan	---	19	---	---	Pakistan Standards and Quality Control Authority (Undated)	English
73	Republic of Palau	---	6	---	---	Environmental Quality Protection Board (Undated)	English
74	Republic of Panama	20	---	Panama Ministry of Economy and Finance (2009)	Spanish	---	---
75	Republic of Peru	4	45	Peru Ministry of Environment (2013)	Spanish	Peru Ministry of Health (2011)	Spanish
76	Republic of the Philippines	---	17	---	---	Philippines Department of Health (2007)	English
77	Republic of Poland	14	UNK	Poland Minister of the Environment (2002)	Polish	Poland Ministry of Health (2007)	Polish
78	Portuguese Republic	15	UNK	Ontario Ministry of Environment and Energy (1997)	Portuguese and English	Portugal Ministry of Environment, Planning and Regional Development (2007)	Portuguese
79	State of Qatar	4	33	Qatar Ministry of Environment (2007)	Arabic	The Gulf Cooperation Council (GCC) Standardization (2012)	Arabic & English
80	Russian Federation	146	106	Russian State Construction Code (1997)	Russian	Russian Ministry of Health (1998, 1999, 2002, 2007)	Russian
				Russian Ministry Of Environment and Natural Resources (1993)	Russian		
	(Russia) City of Moscow	1	---	Moscow Government (2004)	Russian	---	---
	(Russia) Republic of Tatarstan	137	---	Republic of Tatarstan Ministry of Environment and Natural Resources (2002)	Russian	---	---
81	Republic of Rwanda	---	19	---	---	Rwanda Standards Board (2013)	English
82	Saint Lucia	---	40	---	---	Caricom Regional Organization for Standards and Quality (undated)	English
83	Republic of Serbia	56	28	Serbia Ministry of Environment and Spatial Planning (1994)	English	Serbia Official Gazette (1999)	English
84	Republic of Singapore	46	39	Singapore National Environmental Agency (2010)	English	Government of Singapore (2008)	English
85	Slovak Republic	5	UNK	Slovakia Ministry of Agriculture (2004)	Slovak	Council Regulation Government of the Slovak Republic (2010)	Slovak
86	Republic of Slovenia	45	UNK	Slovenia Ministry of Environment and Spatial Planning (1996)	Slovenian	Slovenia Ministry of Health (2004)	Slovenian
87	Republic of South Africa	10	1	South Africa Department of Environmental Affairs (2010)	English	South Africa Department of Water and Sanitation (2005)	English
88	Kingdom of Spain	14	UNK	Spain Ministry of the Presidency (2005)	Spanish	Government of Spain (2003)	Spanish
	(Spain) Autonomous Community of Andalusia	19	---	Andalusia Ministry of Environment (2006)	Spanish	---	---
	(Spain) Autonomous Community of Aragon	14	---	Government of Aragon (2005)	Spanish	---	---
	(Spain) Principality of Asturias	14	---	The Government of the Principality of Asturias (2005)	Asturianu	---	---
	(Spain) Autonomous Community Balearic Islands	14	---	Ministry of Agriculture, Environment and Territory of Balearic Islands (2013)	Catalan	---	---
	(Spain) Basque Country	17	---	Basque Government, Department of Environment, Planning, Agriculture and Fisherie (2005)	Basque	---	---
	(Spain) Autonomous Community of Canary Islands	14	---	Government of Canary Islands (2007)	Spanish	---	---
	(Spain) Autonomous Community of Cantabria	14	---	Government of Cantabria (2006)	Spanish	---	---
	(Spain) Autonomous Community of Castile and Leon	14	---	Government of Castile and Leon	Spanish	---	---
	(Spain) Autonomous Community of Castile La Mancha	14	---	Jiménez Ballesta et al. (2010)	Spanish	---	---
	(Spain) Autonomous Community of Catalonia	23	---	Waste Agency of Catalonia (2005)	Catalan	---	---
				Andalusia Ministry of Environment (2006)	Spanish		
	(Spain) Autonomous City of Ceuta	14	---	Official Portal of Ceuta (2013)	Spanish	---	---
	(Spain) Autonomous Community of Extremadura	14	---	Ministry of Agriculture and Rural Development (2010)	Spanish	---	---
	(Spain) Autonomous Community of Galicia	23	---	Ministry of Environment and Sustainable Development of Galicia (2009)	Galician	---	---
				Andalusia Ministry of Environment (2006)	Spanish		
	(Spain) Autonomous Community of La Rioja	14	---	Government of La Rioja (2007)	Spanish	---	---
	(Spain) Autonomous Community of Madrid	14	---	Spain Ministry of the Presidency (2005)	Spanish	---	---
	(Spain) Autonomous City of Melilla	14	---	Ministry of Environment of the Autonomous City of Melilla (undated)	Spanish	---	---
	(Spain) Region of Murcia	14	---	Government of Region of Murcia (2011)	Spanish	---	---
	(Spain) Autonomous Community of Navarra	14	---	Department of Rural Development, Environment and Local Government (undated)	Basque	---	---
	(Spain) Autonomous Community of Valencia	14	---	Generalist at Valencian Regional Ministry of Infrastructure, Planning and the Environment (2007)	Catalan	---	---
89	Republic of the Sudan	---	36	---	---	World Health Organization Regional Office for the Eastern Mediterranean (2006)	English
90	Kingdom of Sweden	---	UNK	---	---	Sweden Nutrition and Food Agency (2001)	Swedish
91	Swiss Confederation	---	UNK	---	---	Switzerland Department of Consumer and Veterinary (2014)	French
92	Syrian Arab Republic	---	12	---	---	World Health Organization Regional Office for the Eastern Mediterranean (2006)	English
93	United Republic of Tanzania	17	1	Tanzanian Bureau of Standards (2007)	English	Tanzania Bureau of Standards (2009)	English
94	Kingdom of Thailand	9	1	Thailand Ministry of Natural Resources and Environment (2004)	English	Thailand Ministry of Health (2001)	Thai
95	Kingdom of Tonga	---	36	---	---	Secretariat of the Pacific Community (2005)	English
96	Republic of Turkey	1	---	Turkey Ministry of Environment and Forestry (2001)	Turkish	---	---
97	Republic of Tunisia	---	1	---	---	Global Water and Wastewater Quality Regulations (2012)	English
98	Tuvalu	---	36	---	---	Secretariat of the Pacific Community (2005)	English
99	Republic of Uganda	---	34	---	---	Uganda Ministry of Tourism, Trade and Industry (2008)	English
100	Ukraine	286	UNK	Ministry of Health of Ukraine (2001)	Ukrainian	Ukraine Water Health (Undated)	Russian
101	United Kingdom of Great Britain and Northern Ireland	---	UNK	---	---	United Kingdom Drinking Water Inspectorate (2000)	English
	(United Kingdom) Northern Ireland	---	UNK	---	---	Statutory Rules of Northern Ireland (2007)	English
	(United Kingdom) Anglian Water Services	1	---	Anglian Water Services Ltd. (2010)	English	---	---
	(United Kingdom) White Young Green Environmental Ltd	2	---	White Young Green Environmental Ltd. (2008)	English	---	---
	(United Kingdom) Environmental Industries Commission	36	---	Environmental Industries Commission (2010)		---	---
102	Eastern Republic of Uruguay	---	41	---	---	Uruguay Administration of Sanitary Works (2006)	Spanish
103	Republic of Uzbekistan	104	2	Head of State health officer of the Republic of Uzbekistan (2005)	Russian	Uzbekistan Ministry of Health (2006)	Russian
104	Republic of Vanuatu	---	36	---	---	Secretariat of the Pacific Community (2005)	English
		---	UNK	---	---	Secretariat of the Pacific Community (2005)	English
105	Bolivarian Republic of Venezuela	---	16	---	---	Venezuela Ministry of Health And Welfare (1998)	Spanish
106	Socialist Republic of Vietnam	60	36	Republic of Vietnam (2008)	Vietnamese	Viet Nam Ministry of Health (2002)	Vietnamese
				Republic of Vietnam (1995)	Vietnamese		
*Jurisdictions other than United Nations member states*							
1	Palestine	---	20	---	---	Palestinian Water Authority (1997)	Arabic
2	Union of Soviet Socialist Republics (USSR)	197	6	The State Standard of the USSR (1983)	Russian	Medical Officer of the USSR (1981), State Sanitary of the USSR (1987)	Russian
				State Medical Officer of the USSR (1982)	Russian		
				the USSR Ministry (1991)	Russian		
				Ministry of Health of the USSR (1980)	Russian		

^1^ The reference websites of the worldwide pesticide soil RGVs and drinking water MCLs were provided in supplement materials. ^2^ UNK—The European Union and some nations promulgated pesticide standard values for distinct classes of pesticides, but as the members of these classes are not specified individually, the total number of standard values is unknown. ^3^ Notation --- indicates that the nations, regions, or organizations did not provide any pesticide standard values.

**Table 2:** U.S. pesticide RGVs and MCLs jurisdiction sources for state, regions, U.S. territories, and national organizations.

No.	U.S. Jurisdictions	No. of Soil RGVs	No. of Water MCLs	Sources of U.S. Pesticide Soil RGVs ^1^	Sources of U.S. Pesticide Water MCLs ^1^
*U.S. national organization jurisdictions*					
1	U.S. Environmental Protection Agency	516	24	U.S. Environmental Protection Agency (2013)	U.S. Environmental Protection Agency (2009)
2	National Oceanic and Atmospheric Administration	39	--- ^2^	National Oceanic and Atmospheric Administration Office of Response and Restoration (2008)	---
3	National Aeronautics and Space Administration	20	---	Boeing Company, National Aeronautics and Space Administration and Department of Energy (2010)	---
4	Department of Energy	20	---	Boeing Company, National Aeronautics and Space Administration and Department of Energy (2010)	---
5	Food and Drug Administration	---	24	---	Food and Drug Administration (2013)
6	U.S. Army Public Health Command	---	520	U.S. Army Center for Health Promotion and Preventive Medicine (2013)	U.S. Army Public Health Command (2013)
	U.S. Army	259	---	---	---
7	Agency of Toxic Substance and Disease Registry	26	---	Agency of Toxic Substance and Disease Registry (2008, 2009a, f, 2010c, 2013)	---
*U.S. state and regional jurisdictions*					
1	State of Alabama	59	24	Alabama Department of Environmental Management (2008)	Alabama Department of Environmental Management (undated *)
2	State of Alaska	87	27	Alaska Department of Environmental Conservation (2012)	Alaska Department of Environmental Conservation (2008)
3	State of Arizona	523	24	Arizona Administrative Code (2009)	Arizona Department of Environmental Quality (2008)
				Arizona Department of Environmental Quality (2002)	
4	State of Arkansas	519	24	Arkansas Department of Environmental Quality (2008)	Arkansas Department of Environmental Quality (2013)
				U.S. Environmental Protection Agency (2013)	
5	State of California	16	35	California Environmental Protection Agency Office of Environmental Health Hazard Assessment (2010)	California Office of Environment Health Hazard Assessment (2010)
			2		California Department of Public Health (2010)
			15		California Department of Health Services (2010)
			27		California Department of Health Services (2014)
	(California) City of Oakland	4	---	City of Oakland Public Works Agency (2000)	
	(California) San Francisco Bay Regional Water Quality Control Board	40	---	San Francisco Bay Regional Water Quality Control Board (2013)	
6	State of Colorado	551	28	Colorado Department of Public Health and Environment (2011)	Colorado Department of Public Health and Environment (2014)
7	State of Connecticut	14	24	Department of Energy and Environmental Protection (2013)	Connecticut Department of energy and environmental protection (2013)
			7		Connecticut Department of energy and environmental protection (2014)
8	State of Delaware	413	24	Delaware Department of Natural Resources and Environmental Control (1999, 2013)	Delaware Department of Natural Resources and Environmental Control (undated)
9	State of Florida	143	85	Florida Department of Environmental Protection (2005)	Florida Department of Health (2014)
	(Florida) Miami-Dade County	142	---	Code of Miami-Dade County (2008)	---
10	State of Georgia	151	---	Georgia Department of Natural Resources (1993)	---
11	State of Hawaii	30	24	Hawaii Department of Health (2011)	Hawaii Department of Health (2009)
12	State of Idaho	47	24	Idaho Department of Environmental Quality (2004)	Idaho Department of Environmental Quality (2014)
13	State of Illinois	68	13	Illinois Administrative Code (2010)	Illinois Environmental Protection Agency (undated)
				Illinois Environmental Protection Agency (2011)	
14	State of Indiana	215	24	Indiana Department of Environmental Management (2013)	Indiana Department of Environmental Management (undated)
			24		Indiana Department of Environmental Management (1996)
15	State of Iowa	94	24	Iowa Department of Natural Resources (2013)	Iowa Department of Nature Resources (2012)
16	State of Kansas	62	27	Kansas Department of Health and Environment (2010)	Kansas Department of Health and Environment (2004)
17	Commonwealth of Kentucky	516	24	Kentucky Energy and Environmental Cabinet (2011)	Kentucky Department of environmental protection (2010)
				U.S. Environmental Protection Agency (2013)	
18	State of Louisiana	22	24	Louisiana Department of Environmental Quality (2003)	Louisiana Department of Health and Hospital (undated)
19	State of Maine	545	24	Maine Department of Environmental Protection (2011, 2013)	Government of Maine (undated)
				U.S. Environmental Protection Agency (2013)	
20	State of Maryland	33	24	Maryland Department of the Environment (2008)	Maryland Department of Environment (undated)
21	Commonwealth of Massachusetts	119	24	Massachusetts Department of Environmental Protection (2014)	Massachusetts Office of Energy and Environmental Affairs Energy and Environmental Affairs (2012)
22	State of Michigan	62	24	Michigan Department of Environmental Quality (2012)	Michigan Department of Environmental Quality (2014)
23	State of Minnesota	132	27	Minnesota Pollution Control Agency (2009)	Minnesota Department of Health (2011)
24	State of Mississippi	113	24	Mississippi Department of Environmental Quality (2002)	Mississippi Department of Health (2013)
25	State of Missouri	309	24	Missouri Department of Natural Resources (2010)	Missouri Department of Natural Resources (1996)
26	State of Montana	516	24	Montana Department of Environmental Quality (2012)	Montana Department of Environmental Quality (2004)
				U.S. Environmental Protection Agency (2013)	
27	State of Nebraska	215	24	Nebraska Department of Environmental Quality (2012)	Nebraska Department of Health and Human Services (2012)
28	State of Nevada	386	24	Nevada Division of Environmental Protection (2009, 2013)	Nevada Division of Environmental Protection (2013)
29	State of New Hampshire	87	27	New Hampshire Code of Administrative Rules (2008)	New Hampshire Department of Environmental Services (2013)
30	State of New Jersey	51	24	New Jersey Department of Environmental Protection (1999, 2012)	New Jersey Department of Environmental Protection (2011)
			3		New Jersey Department of Environmental Protection (2009)
31	State of New Mexico	511	24	New Mexico Environment Department (2012)	New Mexico Environment Department (2003)
				U.S. Environmental Protection Agency (2013)	
32	State of New York	69	21	New York State Department of Environmental Conservation (2006, 2010)	New York Department of Health (2011)
	(New York) New York City	63	---	New York State Department of Environmental Conservation (2006)	---
	(New York) Suffolk County	3	---	Suffolk County Department of Health Services (2011)	---
33	State of North Carolina	304	24	North Carolina Department of Environment and Natural Resources (2005, 2012, 2013)	North Carolina Division of Water Resources (2011)
34	State of North Dakota		24		North Dakota Department of Health (2005)
35	State of Ohio	437	24	Ohio Administrative Code (2009)	Ohio Environmental Protection Agency (UNDATED)
				Ohio Environmental Protection Agency (2005, UNDATED)	
				U.S. Environmental Protection Agency (2013)	
36	State of Oklahoma	516	24	Oklahoma Department of Environmental Quality (2013)	Oklahoma Department of Environmental Quality (2012)
				U.S. Environmental Protection Agency (2013)	
37	State of Oregon	608	24	Oregon Department of Environmental Quality (2010, 2012)	Oregon Department of Environmental Quality (2000)
			20		Oregon Public Health (2012)
				U.S. Environmental Protection Agency (2013)	
38	Commonwealth of Pennsylvania	134	24	Pennsylvania Department of Environmental Protection (2014)	Pennsylvania Department of Environmental Protection (2006)
39	Rhode Island	7	24	Rhode Island Department of Environmental Management (2011)	Rhode Island Department of Health (2011)
40	State of South Carolina	---	24	---	South Carolina Department of Health and Environment (2009)
41	State of South Dakota	---	27	---	South Dakota Department of Environment and Natural Resources (undated)
42	State of Tennessee	516	24	Tennessee Department of Environment and Conservation (2001)	Tennessee Department of Environment and Conservation (undated)
43	State of Texas	1140	24	Texas Commission on Environmental Quality (2003, 2006a, b, 2012)	Texas Commission on Environmental Quality (2013)
44	State of Utah	---	24	---	Utah Department of Environmental Quality (2014)
45	State of Vermont	754	24	Vermont Department of Environmental Conservation (2012)	Vermont Agency of Natural Resources (2010)
			2		Vermont Department of Health (2002)
				U.S. Environmental Protection Agency (2013)	
46	Commonwealth of Virginia	347	24	Virginia Department of Environmental Control (UNDATED)	Virginia Department of Health (2014)
47	State of Washington	252	---	Washington Department of Ecology (2007, 2014)	---
48	State of West Virginia	326	24	West Virginia Department of Environmental Protection (2009a, b)	Business and Legal Resources (2014)
49	State of Wisconsin	237	24	Wisconsin Department of Natural Resources (2013)	Wisconsin Department of Natural Resources (2013)
50	State of Wyoming	25	38	Wyoming Department of Environmental Quality (2013)	Wyoming Department of Environmental Quality (2013)
*U.S. territories*					
1	Commonwealth of the Northern Mariana Islands	128	23	Commonwealth of the Northern Mariana Islands Division of Environmental Quality (2012)	CNMI Division of Environmental Quality (2005)
2	Unincorporated Territory of Guam	128	6	Guam Environmental Protection Agency (2012)	Guam Environmental Protection Agency (1997)
*Autonomous native American jurisdictions*					
1	The Confederated Tribes of the Colville Reservation	1	---	Colville Confederated Tribes (2008)	---
2	Confederated Tribes of the Coos-Lower Umpqua-Siuslaw Indians	608	---	Confederated Tribes of the Coos-Lower Umpqua-Siuslaw Indians (2010)	---
				Oregon Department of Environmental Quality (2010, 2012a, b, 2014)	
				U.S. Environmental Protection Agency (2013)	
3	Hoppa Valley Tribe	239	---	Hoppa Valley Tribe (2008)	---
				U.S. Environmental Protection Agency Region IX (2004)	
4	Metlakatla Indian Community	2	---	Metlakatla Indian Community (2011)	---
5	Nez Perce Tribe	41	---	Nez Perce Tribe (2009)	---
6	Jamestown S'Klallam Tribe	252	---	Jamestown S'Klallam Tribe (2010)	---
				Washington Department of Ecology (2001, 2014)	
7	Shoshone-Bannock Tribes	20	---	Shoshone-Bannock Tribes (2010)	---

^1^ The reference websites of the U.S. pesticide soil RGVs and drinking water MCLs were provided in supplement materials. ^2^ Notation --- indicates that the nations, regions, or organizations did not provide any pesticide standard values. * Undated—The date on which jurisdictions were generated, revised, drafted, or published is unavailable.

**Table 3:** Estimated intake rates for the most consumed agricultural commodities

Crop Type	Agricultural Commodity	Intake Rate Estimated (kg/day)
Fruit crops	Apple	0.019
	Banana	0.032
	Grape	0.009
	Orange	0.028
Vegetable crops	Potato	0.042
	Tomato	0.021
	Onion	0.023
Grain crops	Rice	0.156
	Wheat	0.223
	Maize	0.042
Drink crops	Tea	0.001
	Coffee	0.012

**Table 4:** Summary of the most commonly regulated pesticides in residential surface soil and drinking water.

No.	Pesticide Common Name	CAS No.	No. of RGVs (U.S., world)	RGV Lowest Value (mg/kg)	RGV Highest Value (mg/kg)	RGV LOV	No. of MCLs (U.S., world)	MCL Lowest Value (mg/L)	MCL Highest Value (mg/L)	MCL LOV
1	DDT	50-29-3	319 (140, 179)	0.00033; Oregon + ^1^	11,300.0; Netherlands	7.53	115 (13, 102)	0.00000022; CNMI ^2^	2.8; U.S. Military	7.11
2	Lindane	58-89-9	247 (133, 114)	0.000005; Poland	707.0; New Zealand	8.15	166 (58, 108)	0.000019; CNMI	2; Mexico +	5.02
3	Dieldrin	60-57-1	247 (121, 126)	0.0000081; Oregon +	63,000.0; Illinois	9.89	109 (6, 103)	0.000000052; CNMI	0.03; Mauritius +	5.76
4	DDE	72-55-9	244 (118, 126)	0.00033; Oregon +	7830.0; Netherlands	7.38	76 (4, 72)	0.00000022; Wyoming +	1.0; Mexico	6.66
5	DDD	72-54-8	243 (122, 121)	0.00033; Oregon +	5160.0; U.S. Military	7.19	74 (3, 71)	0.00000022; Wyoming	1.0; Mexico	6.66
6	Aldrin	309-00-2	242 (119, 123)	0.00006; Serbia +	1000.0; Guam +	7.22	110 (6, 104)	0.000000049; Wyoming+	0.03; Hungary +	5.79
7	Chlordane	57-74-9 or 12789-03-6	224 (143, 81)	0.00003; Serbia +	1000.0; Guam +	7.52	163 (61, 102)	0.0000008; Wyoming +	0.2; Mexico +	5.40
8	Endrin	72-20-8	217 (129, 88)	0.00004; Singapore +	4240.0; U.S. Military	8.03	136 (60, 76)	0.000005; Gambia	0.28; U.S. Military	4.75
9	Heptachlor	76-44-8	212 (136, 76)	0.0003; Serbia	1000.0; Guam +	6.52	137 (59, 78)	0.00000079; Wyoming +	0.05; Russia	5.80
10	Pentachloropnenol	87-86-5	191 (125, 66)	0.005; Norway	6500.0; Ohio	6.11	153 (57, 96)	0.0001; EU +	9.0; Vietnam	4.95
11	Endosulfan	115-29-7	177 (103, 74)	0.00001; Singapore +	3000.0; Massachusetts	8.48	13 (1, 12)	0.02; Australia +	0.07; U.S. Military	0.54
12	Heptachlor Epoxide	1024-57-3	166 (121, 45)	0.0000002; Serbia +	1000.0; Guam +	9.70	117 (57, 60)	0.00000039; Wyoming+	0.1; Croatia	6.41
13	α-HCH	319-84-6	162 (90, 72)	0.00011; North Carolina	7100.0; Texas	7.81	7 (4, 3)	0.0000026; Wyoming+	0.02; (Australia) Queensland	3.88
14	Methoxychlor	72-43-5	158 (118, 40)	0.046; Alberta	9170.0; Missouri	5.30	159 (58, 101)	0.0001; EU +	20.0; Mauritius +	5.30
15	β-HCH	319-85-7	154 (74, 80)	0.00037; North Carolina	127.0; U.S. Military	5.54	10 (8, 2)	0.0000091; Wyoming+	0.7; U.S. Military	4.89
16	2,4-D	94-75-7	147 (103, 44)	0.04; Moldova	12,000.0; Oregon +	5.78	180 (59, 121)	0.0001; EU +	30.0; Mexico +	5.48
17	Atrazine	1912-24-9	144 (76, 68)	0.00005; Poland	12,000.0; Texas	8.38	163 (58, 105)	0.0001; EU +	2.0; Vietnam	4.30
18	Toxaphene	8001-35-2	142 (102, 40)	0.00042; SFBWQ	500.0; Guam +	6.08	99 (54, 45)	0.0000028; Wyoming +	0.014; U.S. Military	4.70
19	Bromomethane	74-83-9	107 (106, 1)	0.0501; Idaho +	10,000.0; Massachusetts	5.30	8 (0, 8)	0.001; Australia +	0.002; Argentina	0.30
20	o-Cresol	95-48-7	105 (78, 27)	0.33; New York +	106,000.0; U.S. Military	5.51	1 (1, 0)	7.0; U.S. Military	7.0; U.S. Military	0.00
21	Simazine	122-34-9	103 (72, 31)	0.01; Armenia +	1800.0; Texas	5.26	157 (56, 101)	0.0001; EU +	20.0; Vietnam	5.30
22	Chlorpyrifos	2921-88-2	102 (66, 36)	0.20; Russia +	11,000.0; Michigan	4.74	90 (6, 84)	0.0001; EU +	0.09, Argentina +	2.95
23	Aldicarb	116-06-3	78 (68, 10)	0.041; Idaho +	360.0; Texas	3.94	103 (16, 87)	0.0001; EU +	10.0; Vietnam	5.00
24	Oxamyl	23135-22-0	71 (67, 4)	0.386; Idaho +	8900.0; Texas	4.36	102 (52, 50)	0.0001; EU +	0.35; U.S. Military	3.54
25	Dinoseb	88-85-7	85 (75, 10)	0.163; Idaho +	360.0; Texas	3.34	100 (55, 45)	0.0001; EU +	0.42; U.S. Military	3.62
26	Methoxychlor	72-43-5	158 (118, 40)	0.046; Alberta	9170.0; Missouri	5.30	159 (58, 101)	0.0001; EU +	20.0; Mauritius +	5.30
27	Simazine	122-34-9	103 (72, 31)	0.01; Armenia +	1800.0; Texas	5.26	157 (56, 101)	0.0001; EU +	20.0; Vietnam	5.30
28	Alachlor	15972-60-8	67 (62, 5)	0.008; North Carolina	3600.0; Texas	5.65	141 (61, 80)	0.0001; EU +	20.0; Vietnam	5.30
29	2,4,5-TP	93-72-1	96 (94, 2)	0.8; Minnesota +	3000.0; Ohio	3.57	138 (53, 85)	0.0001; EU +	9.0; Vietnam	4.95
30	Carbofuran	1563-66-2	97 (63, 34)	0.00002; Singapore +	1800.0; Texas	7.95	138 (41, 97)	0.0001; EU +	0.1; Serbia	3.00
31	DBCP	96-12-8	31 (30, 1)	0.003; Georgia	16.0; Washington	3.73	125 (51, 74)	0.00003; Vermont	1.0; Vietnam	4.52
32	Glyphosate	1071-83-6	93 (73, 20)	0.011; Guam +	36,000.0; Texas	6.51	122 (56, 66)	0.0001; EU +	28.0; U.S. Military	5.45
33	Picloram	1918-02-1	91 (64, 27)	0.022; Alberta	25,000.0; Texas	6.06	119 (54, 65)	0.0001; EU +	28.0; U.S. Military	5.45
34	Diquat	85-00-7	71 (53, 18)	0.109; Nez Perce Tribe	480.0; Pennsylvania	3.64	115 (26, 89)	0.0001; EU +	0.1; Argentina	3.00
35	Endothall	145-73-3	70 (66, 4)	0.335; Idaho +	7100.0; Texas	4.33	104 (54, 50)	0.0001; EU +	1.1; U.S. Military	4.04
36	Dalapon	75-99-0	90 (75, 15)	0.10; Vietnam	19,000.0; Michigan	5.28	104 (54, 50)	0.0001; EU +	4.2; U.S. Military	4.62
37	Aldicarb	116-06-3	78 (68, 10)	0.041; Idaho +	360.0; Texas	3.94	103 (16, 87)	0.0001; EU +	10.0; Vietnam	5.00
39	Dinoseb	88-85-7	85 (75, 10)	0.163; Idaho +	360.0; Texas	3.34	100 (55, 45)	0.0001; EU +	0.42; U.S. Military	3.62

^1^ “+” indicates that the jurisdiction is one of the multiple jurisdictions specifying the value. ^2^ CNMI is the abbreviation of Commonwealth of the Northern Mariana Islands.

**Table 5:** Statistical summary of the RGVs for the commonly regulated pesticides.

No.	Pesticide	CAS No.	RGV Number	Arithmetic Mean (mg/kg)	Median (mg/kg)	Geometric Mean (mg/kg)	Pearson Coefficient	No. of RGVs in Data Clusters	%	Cancer risk Uncertainty Bounds (mg/kg)	Non-cancer risk Uncertainty Bounds (mg/kg)	No. of RGVs above All Risk Upper Bounds	%
1	2,4-D	94-75-7	147	12000	630	122	0.907	49	33%	--- ^1^	(45.3, 690)	6	4%
2	Aldrin	309-00-2	241	11.3	0.1	0.24	0.977	89	37%	(0.016, 0.1)	(0.91, 9.61)	27	11%
3	Atrazine	1912-24-9	144	12000	2.2	3.61	0.983	52	36%	(1.2, 7.4)	(1100, 11200)	1	1%
4	Carbaryl	63-25-2	94	4079	905	177.1	0.918	49	52%	---	(3000, 32,100)	1	1%
5	Carbofuran	1563-66-2	97	200.4	130	15	0.935	54	56%	---	(150, 1610)	1	1%
6	Chlordane	57-74-9 or 12789-03-6	224	41.3	2.8	3	0.990	69	31%	(1.13, 5.63)	(23.0, 190.0)	11	5%
7	Chlorpyrifos	2921-88-2	102	235.8	61	37.7	0.960	50	49%	---	(30, 321)	12	12%
8	DDT	50-29-3	319	93.2	2	3.38	0.977	97	30%	(1.25, 5.95)	(26.0, 200.0)	31	10%
9	Diazinon	333-41-5	86	38.8	35.5	10.1	0.945	56	65%	---	(21, 220)	2	2%
10	Dicamba	1918-00-9	80	1340.6	1800	164.3	0.903	50	63%	---	(910, 9600)	1	1%
11	Dieldrin	60-57-1	247	266.7	0.15	0.28	0.986	60	24%	(0.016, 0.11)	(1.5, 16.0)	19	8%
12	Diuron	330-54-1	88	138.2	120	38.5	0.948	41	47%	---	(60, 640)	3	3%
13	Endosulfan	115-29-7	177	219.7	37	17.9	0.965	56	32%	---	(181.5, 1928)	2	1%
14	Endrin	72-20-8	217	42.3	4.6	2.63	0.969	79	36%	---	(9.1, 96.0)	12	6%
15	Glyphosate	1071-83-6	93	4715.7	1500	545.8	0.904	48	52%	---	(3000, 32,000)	1	1%
16	α-HCH	319-84-6	162	84.7	0.1	0.24	0.952	100	62%	(0.04, 0.27)	(241.9, 2571)	2	1%
17	β-HCH	319-85-7	154	4.72	0.3	0.28	0.983	80	52%	(0.15, 0.95)	---	49	32%
18	γ-HCH (Lindane)	58-89-9	247	16.1	0.52	0.51	0.989	117	47%	(0.56, 1.7)	(14.1, 114.2)	9	4%
19	t-HCH	608-73-1	84	11.8	0.3	0.438	0.934	36	43%	(0.15, 0.95)	---	22	26%
20	Heptachlor	76-44-8	212	15.9	0.2	0.45	0.978	71	33%	(0.0615, 0.379)	(5.0, 160.0)	4	2%
21	Malathion	121-75-5	85	893	1200	195.8	0.922	52	61%	---	(600, 6400)	1	1%
22	MCPA	94-74-6	98	153.9	31	14.1	0.965	65	66%	---	(15, 160)	20	20%
23	Metolachlor	51218-45-2	83	6105.7	2000	635.8	0.908	48	58%	---	(4500, 48,000)	1	1%
24	Picloram	1918-02-1	91	3581	4300	649.2	0.878	33	36%	---	(2100, 22,000)	1	1%
25	Simazine	122-34-9	103	108	4.1	3.9	0.971	31	30%	(2.3, 14.0)	(150, 1600)	1	1%
26	Toxaphene	8001-35-2	142	23	0.6	1.71	0.952	53	37%	(0.252, 1.55)	---	58	41%
27	Trifluralin	1582-09-8	95	287.5	63	47.1	0.900	47	49%	(26, 220)	(230, 2400)	2	2%

^1^—indicates the U.S. EPA did not consider cancer or non-cancer risk when computing the pesticide RGVs.

**Table 6:** Statistical summary of the MCLs for the commonly regulated pesticides.

No.	Pesticide	CAS No.	MCL No.	Arithmetic Mean (mg/L)	Median (mg/L)	Geometric Mean (mg/L)	Pearson Coefficient	No. of MCLs in Data Clusters	%	Health Risk Uncertainty Bounds (mg/L)	No. of MCLs above Risk Upper Bound	%
1	2,4,5-TP	93-72-1	138	0.088	0.009	0.0057	0.906	70	51%	(0.024, 0.37)	1	1%
2	2,4-D	94-75-7	180	0.4	0.03	0.015	0.861	164	91%	(0.03, 0.47)	6	3%
3	Alachlor	15972-60-8	141	0.22	0.002	0.0066	0.952	123	87%	(0.03, 0.47)	2	1%
4	Aldicarb	116-06-3	103	0.1	0.004	0.0016	0.91	82	80%	(0.003, 0.047)	2	2%
5	Aldrin	309-00-2	110	0.003	0.00003	0.0000712	0.817	83	75%	(0.003, 0.047)	1	1%
6	Atrazine	1912-24-9	163	0.032	0.003	0.0027	0.948	130	80%	(0.015, 0.23)	1	1%
7	Carbofuran	1563-66-2	137	0.025	0.01	0.005	0.938	98	72%	(0.009, 0.14)	0	0%
8	Chlordane	57-74-9 or 12789-03-6	163	0.0067	0.0002	0.0005	0.939	137	84%	(0.0015, 0.023)	8	5%
9	Chlorpyrifos	2921-88-2	90	0.025	0.01	0.003	0.811	81	90%	(0.09, 1.4)	0	0%
10	Dalapon	75-99-0	104	0.26	0.2	0.014	0.872	89	86%	(0.09, 1.4)	5	5%
11	DBCP	96-12-8	125	0.013	0.00035	0.0002	0.909	112	90%	(0.0006, 0.0093)	8	6%
12	DDT	50-29-3	115	0.072	0.001	0.0011	0.945	82	71%	(0.006, 0.093)	9	8%
13	Dieldrin	60-57-1	141	0.003	0.00003	0.0000739	0.803	83	59%	(0.0003, 0.0047)	9	6%
14	Dinoseb	88-85-7	100	0.017	0.007	0.0017	0.878	91	91%	(0.003, 0.047)	6	6%
15	Diquat	85-00-7	115	0.019	0.02	0.0037	0.886	103	90%	(0.006, 0.093)	1	1%
16	Endothall	C8H10O5	104	0.089	0.1	0.0089	0.855	98	94%	(0.09, 1.4)	0	0%
17	Endrin	72-20-8	136	0.0023	0.0006	0.0006	0.945	116	85%	(0.0006, 0.0093)	4	3%
18	Glyphosate	1071-83-6	122	1.04	0.7	0.042	0.864	91	75%	(0.9, 14)	2	2%
19	Heptachlor	76-44-8	137	0.0038	0.0003	0.0002	0.952	85	62%	(0.0015, 0.023)	11	8%
20	Heptachlor Epoxide	1024-57-3	120	0.003	0.0003	0.00013	0.921	93	78%	(0.000039, 0.00061)	14	12%
21	Lindane	58-89-9	167	0.064	0.0002	0.00076	0.935	145	87%	(0.009, 0.14)	9	5%
22	MCPA	94-74-6	94	0.041	0.02	0.015	0.948	80	85%	(0.03, 0.47)	1	1%
23	Methoxychlor	72-43-5	159	0.56	0.02	0.011	0.891	113	71%	(0.3, 4.67)	4	3%
24	Oxamyl	23135-22-0	104	0.11	0.2	0.0089	0.89	89	86%	(0.006, 0.093)	55	53%
25	Pentachloropnenol	87-86-5	153	0.081	0.001	0.0012	0.957	123	80%	(0.015, 0.23)	4	3%
26	Picloram	1918-02-1	119	0.98	0.3	0.031	0.871	103	87%	(0.21, 3.27)	5	4%
27	Simazine	122-34-9	157	0.15	0.004	0.002	0.936	148	94%	(0.015, 0.23)	2	1%
28	Toxaphene	8001-35-2	99	0.003	0.003	0.00072	0.881	90	91%	(0.006, 0.093)	1	1%
29	Trifluralin	1582-09-8	98	0.23	0.02	0.0039	0.907	78	80%	(0.06, 0.93)	1	1%

**Table 7:** Statistical summary of IEDs for the widely used pesticides.

No.	Pesticide	CAS No.	IED No.	Arithmetic mean (mg/kg-day)	Median (mg/kg-day)	Geometric Mean (mg/kg-day)	Pearson Coefficient	No. of IEDs in Data Clusters	%	ADI Value (mg/kg-day)	No. of IEDs above the ADI Value	%
1	2,4-D	94-75-7	91	0.0064	0.0069	0.0056	0.761	63	70%	0.01	1	1%
2	Aldicarb	116-06-3	88	0.00012	0.0000975	0.000764	0.763	67	76%	0.001	0	0%
3	Atrazine	1912-24-9	55	0.00031	0.00038	0.00023	0.830	35	64%	0.005	0	0%
4	Chlorothalonil	1897-45-6	88	0.0045	0.002	0.0027	0.897	59	67%	0.015	0	0%
5	Chlorpyrifos	2921-88-2	88	0.0037	0.0034	0.0031	0.929	58	66%	0.001	85	97%
6	Diazinon	333-41-5	88	0.0003	0.00026	0.00016	0.949	71	81%	0.0007	12	14%
7	Dicamba	1918-00-9	89	0.0059	0.0064	0.0048	0.645	76	85%	0.03	0	0%
8	Diuron	330-54-1	54	0.00049	0.0000505	0.00012	0.741	34	63%	0.002	3	6%
9	Glyphosate	1071-83-6	91	0.093	0.052	0.065	0.907	64	70%	0.1	41	45%
10	Malathion	121-75-5	94	0.045	0.043	0.048	0.951	57	61%	0.02	92	98%
11	Mancozeb	8018-01-7	90	0.0074	0.00026	0.00016	0.949	64	71%	0.03	0	0%
12	MCPA	94-74-6	86	0.00068	0.00064	0.00058	0.616	72	84%	0.0005	76	88%
13	Metolachlor	51218-45-2	51	0.0000887	0.0000757	0.0000798	0.703	42	82%	0.15	0	0%
14	Trifluralin	1582-09-8	53	0.00021	0.00021	0.00018	0.807	42	79%	0.0075	0	0%

**Table 8:** Statistical summary of the IMDLs for the widely used pesticides.

No.	Pesticide	CAS No.	IMDL No.	Arithmetic Mean (mg/kg-day)	Median (mg/kg-day)	Geometric Mean (mg/kg-day)	Pearson Coefficient	No. of IMDLs in Data Clusters	%	No. of IMDLs Computed from Three Exposures	%	ADI (mg/kg-day)	No. of IMDLs above the ADI Value	%
1	2,4-D	94-75-7	145	0.0231	0.00694	0.00214	0.881	71	49%	17	12%	0.01	13	9%
2	Aldicarb	116-06-3	121	0.00259	0.0000975	0.0000971	0.977	74	61%	5	4%	0.001	3	2%
3	Atrazine	1912-24-9	125	0.00167	0.000382	0.000148	0.917	59	47%	22	18%	0.005	2	2%
4	Chlorothalonil	1897-45-6	105	0.004	0.0018	0.0013	0.925	74	70%	2	2%	0.015	0	0%
5	Chlorpyrifos	2921-88-2	129	0.0031	0.0026	0.0015	0.861	78	60%	7	5%	0.001	100	78%
6	Diazinon	333-41-5	108	0.00043	0.00026	0.00011	0.947	69	64%	2	2%	0.0007	20	19%
7	Dicamba	1918-00-9	105	0.00538	0.00639	0.00198	0.713	67	64%	2	2%	0.03	0	0%
8	Diuron	330-54-1	75	0.00112	0.0000533	0.000105	0.946	40	53%	2	3%	0.002	11	15%
9	Glyphosate	1071-83-6	115	0.0765	0.0335	0.0139	0.854	73	63%	4	3%	0.1	42	37%
10	Malathion	121-75-5	111	0.04	0.0482	0.0157	0.688	63	57%	2	2%	0.02	94	85%
11	Mancozeb	8018-01-7	105	0.0067	0.00701	0.0024	0.719	71	68%	2	2%	0.03	0	0%
12	MCPA	94-74-6	126	0.00161	0.00064	0.000289	0.917	82	65%	12	10%	0.0005	87	69%
13	Metolachlor	51218-45-2	77	0.00143	0.0000786	0.0000905	0.921	47	61%	3	4%	0.15	0	0%
14	Trifluralin	1582-09-8	98	0.00628	0.000213	0.00017	0.897	63	64%	2	2%	0.0075	1	1%

## Supplementary Materials

The following are available online at www.mdpi.com/1660-4601/14/7/826/s1, Table S1: Worldwide pesticide soil regulatory jurisdictions references and dates accessed, Table S2: Worldwide pesticide drinking water regulatory jurisdictions references and dates accessed, Table S3: U.S. pesticide soil regulatory jurisdictions references and dates accessed, Table S4: U.S. pesticide drinking water regulatory jurisdictions references and dates accessed, Database: worldwide pesticide standard values.

## References

[1] Li Z. (2016). Analysis of Worldwide Pesticide Regulatory Models and Standards for Controlling Human Health Risk. Ph.D. Thesis.

[2] Association of Environmental Health and Science AEHS Foundation 2003 Survey of States’ Soil and Groundwater Cleanup Standards. http://www.aehsfoundation.org/State--Surveys.aspx.

[3] Bartsch C., Dorfman B. Brownfields, VCPs and Housing: State-of-the-art Information and Data. http://nemw.org/NAHBresults.pdf.

[4] Davis A., Sherwin D., Ditmars R., Hoenke K.A. (2001). An analysis of soil arsenic records of decision. Environ. Sci. Technol..

[5] Proctor D.M., Shay E.C., Scott P.K. (1997). Health-based soil action levels for trivalent and hexavalent chromium: A comparison with state and federal standards. J. Soil Contam..

[6] Schäfer K.W. International Experience and Expertise in Registration Investigation, Assessment, and Clean-Up of Contaminated Military Sites, R&D Project 103 40 102/01, Berlin, Germany: Dames & Moore GmbH & Co. KG. http://www.umweltbundesamt.de/boden-und-altlasten/altlast/web1/berichte/mooreeng/dmeng01.htm.

[7] Interstate Technology Regulatory Council Examination of Risk-Based Screening Values and Approaches of Selected States. http://www.itrcweb.org/Documents/RISK-1.pdf.

[8] Paustenbach D.J., Fehling K., Scott P., Harris M., Kerger B.D. (2006). Identifying soil cleanup criteria for dioxins in urban residential soils: How have 20 years of research and risk assessment experience affected the analysis?. J. Toxicol. Environ. Health.

[9] California Department of Public Health MCL Review in Response to PHGs. http://www.cdph.ca.gov/certlic/drinkingwater/Pages/MCLReview2013.aspx.

[10] Bamidele C. Comparison and Contrast of WHO Drinking Water Standard with Regulatory Standards Such As EU, USEPA, NESREA and Canada. http://www.academia.edu/12231266/COMPARISON_AND_CONTRAST_OF_WHO_DRINKING_WATER_STANDARD_WITH_REGULATORY_STANDARDS_SUCH_AS_EU_USEPA_NESREA_AND_CANADA.

[11] Lenntech EU’s Drinking Water Standards. http://www.lenntech.com/applications/drinking/standards/eu-s-drinking-water-standards.htm.

[12] World Health Organization Health Topics: Pesticides. http://www.who.int/topics/pesticides/en/.

[13] World Health Organization The WHO Recommended Classification of Pesticides by Hazard and Guidelines to Classification 2009. http://www.who.int/ipcs/publications/pesticides_hazard_2009.pdf.

[14] Stoytcheva M. Pesticides in the Modern World—Trends in Pesticides Analysis. https://www.intechopen.com/books/pesticides-in-the-modern-world-trends-in-pesticides-analysis.

[15] National Institute of Standards and Technology (NIST) Lindane. http://webbook.nist.gov/cgi/cbook.cgi?ID=58-89-9&Units=SI.

[16] Global MRL Database Agricultural Commodities Pesticide MRLs. http://www.mrldatabase.com/.

[17] Netherlands Department of Agriculture, Nature and Food Quality An Overview of China’s Fruit and Vegetables Industry. http://china.nlambassade.org/binaries/content/assets/postenweb/c/china/zaken-doen-inchina/import/kansen_en_sectoren/tuinbouw/rapporten_over_tuinbouw/an-overview-ofchinas-fruit-and-vegetables-industry.pdf.

[18] China Ministry of Agriculture Chinese Agricultural Statistics Report (In Chinese). http://www.doc88.com/p-94355314872.html.

[19] India for Safe Food Agency Pesticide Use in India. http://indiaforsafefood.in/farminginindia.

[20] Philippine Statistics Authority Agricultural Production by Type of Crop 2010 to 2012. http://www.nscb.gov.ph/secstat/d_agri.asp.

[21] Federal Office of Consumer Protection and Food Safety Sales of Pesticides in the Federal Republic of Germany (In German). https://www.bund.net/fileadmin/bundnet/pdfs/chemie/pestizide/150213_bund_chemie_einkaufscheck_pestizidverkauf.

[22] Food and Environment Research Agency Pesticide Usage Survey Report 254 Amenity Pesticides in the United Kingdom. https://secure.fera.defra.gov.uk/.../surveys/2012surveys.cfm.

[23] Health Canada Pest Control Products Sales Report for 2009. http://www.hcsc.gc.ca/cps-spc/pubs/pest/_corp-plan/index-eng.php.

[24] Statistics Canada World and Canadian Production of Major Grains and Oilseeds. http://www.statcan.gc.ca/pub/96-325-x/2014001/article/11913-eng.htm.

[25] U.S. Department of Agriculture, National Agricultural Statistics Service Top Vegetable Crops. http://www.agcensus.usda.gov/Publications/2007/Online_Highlights/Fact_Sheets/Production/vpm.pdf.

[26] U.S. Department of Agriculture, National Agricultural Statistics Service The 2007 Census of Agriculture. Fruit, Berries and Tree Nuts. http://www.agcensus.usda.gov/Publications/2007/Online_Highlights/Fact_Sheets/Production/fbn.pdf.

[27] U.S. Department of Agriculture, National Agricultural Statistics Service Crop Production. http://www.epa.gov/agriculture/ag101/cropmajor.html.

[28] Mexico Ministry of Agriculture Mexico’s Top Agricultural Products. http://www.tnvmanagement.com/mexecon-blog/2010/3/8/mexico's-top-agriculturalproducts.aspx.

[29] United Nations Environment Programme Import of Pesticides in Costa Rica: Period 2007–2009. http://cep.unep.org/repcar/informacion-depaises/costarica/Impoortaciones_07-09_REPCar.pdf.

[30] Brazilian Agricultural Research Corporation - Embrapa Vegetable Production in Brazil (In Portuguese). http://www.cnph.embrapa.br/paginas/hortalicas_em_numeros/producao_hortalicas.pdf.

[31] Brazil Department of Rural Economy Fruit Culture — Scenario Analysis of Agricultural (In Portuguese). http://www.agricultura.pr.gov.br/arquivos/File/deral/Prognosticos/fruticultura_2012_13.pdf.

[32] Manktelow D., Stevens P., Walker J., Gurnsey S., Park N., Zabkiewicz J., Teulon D., Rahman A. Trends in Pesticide Use in New Zealand: 2004. http://www.dioxinnz.com/Spray-NZ-Hist/PDF/nz-pesticide-trends.pdf.

[33] Australian Department of Agriculture, Fisheries and Forestry Statistical Tables. http://data.daff.gov.au/brs/data/warehouse/agcomd9abcc004/agcomd9abcc004201206/AgCommodities2012.Vol2.No2_Stats_Ver1.0.0.pdf.

[34] Australian Government Australian Vegetable Growing Farms. http://data.daff.gov.au/data/warehouse/9aab/9aabf/2012/avfesd9abri20121127/AustVegGrwFrmEcoSurvey_1.0.0.pdf.

[35] James M.D., Justinus M.S., Victor W. (2014). Prioritizing agricultural pesticides used in South Africa based on their environmental mobility and potential human health effects. Environ. Int..

[36] U.S. Environmental Protection Agency Regional Screening Levels (RSLs)—Equations (May 2016). https://www.epa.gov/risk/regional-screening-levels-rsls-equations-may-2016.

[37] Jennings A., Li Z. (2014). Scope of the worldwide effort to regulate pesticide contamination in surface soils. J. Environ. Manag..

[38] Jennings A., Li Z. (2015). Residential surface soil guidance applied worldwide to the pesticides added to the Stockholm Convention in 2009 and 2011. J. Environ. Manag..

[39] Jennings A., Li Z. (2015). Residential surface soil guidance values applied worldwide to the original 2001 Stockholm Convention POP pesticides. J. Environ. Manag..

[40] Jennings A., Li Z. (2017). Worldwide Regulatory Guidance Values Applied to Direct Contact Surface Soil Pesticide Contamination: Part II—Noncarcinogenic Pesticides. Air Soil Water Res..

[41] Jennings A., Li Z. (2017). Worldwide regulatory guidance values applied to direct contact surface soil pesticide contamination: Part I—Carcinogenic Pesticides. Air Soil Water Res..

[42] Dorne J.L.C.M. (2010). Metabolism, variability and risk assessment. Toxicology.

[43] U.S. Environmental Protection Agency Reference Dose (RfD): Description and Use in Health Risk Assessments. Background Document 1A March 15, 1993. https://www.epa.gov/iris/reference-dose-rfd-description-and-use-health-risk-assessments.

[44] Jennings A.A. (2010). Analysis of regulatory guidance values for residential surface soil arsenic exposure. J. Environ. Eng..

[45] Australian Drinking Water Guidelines (2011)—Updated February 2016. https://www.nhmrc.gov.au/guidelines-publications/eh52.

[46] World Health Organization Guidelines for Drinking-water Quality Fourth Edition. http://www.who.int/water_sanitation_health/publications/2011/dwq_chapters/en/.

[47] Health Canada Guidelines for Canadian Drinking Water Quality—Summary Table. http://www.hc-sc.gc.ca/ewh-semt/pubs/water-eau/2012-sum_guide-res_recom/index-eng.php.

[48] U.S. Department of Agriculture Data and Statistics. http://www.usda.gov/wps/portal/usda/usdahome?navid=DATA_STATISTICS.

[49] Agency for Toxic Substances and Disease Registry Public Health Assessment Guidance Manual (2005 Update). http://www.atsdr.cdc.gov/hac/PHAManual/appg.html.

