# Supplementary material for: Worldwide Regulations of Standard Values of Pesticides for Human Health Risk Control: A Review
Source: Int J Environ Res Public Health. 2017 Jul 22;14(7):826. doi: 10.3390/ijerph14070826 (PMC5551264; doi:10.3390/ijerph14070826)
Supplement: Supplementary file 1 [file ijerph-14-00826-s001.zip › ijerph-211735-supplementary-2.pdf]

Supplementary materials: Table S1 – S4.

Table S1. Worldwide pesticide soil regulatory jurisdictions references and dates accessed.

Jurisdictions	Sources	Languages	Websites References
Multinational organizations Multinational organizations			
**East Africa Community** (Kenya, Uganda, Tanzania, Rwanda, Burundi)	East Africa Community (2011)	English	East Africa Community, 2011. Thematic Area 2 Environmental Regulations and Standards. http://www.eac.int/infrastructure/ (accessed 09.09.13).
**European Union**	European Union (2010)	English	European Union, 2010. Regulations Commission Regulation (EU) No 756/2010. http://eur-lex.europa.eu/LexUriServ/LexUriServ.do?uri=OJ:L:2010:223:0020:0028:EN:PDF (accessed 09.09.13).
**World Health Organization** (2002)	World Health Organization (2002)	English	World Health Organization, 2002. Developing Human Health-related Chemical Guidelines for Reclaimed Water and Sewage Sludge Applications in Agriculture 2002. http://www.who.int/en/http://www.opaci.org.py/biblioteca/Servicio_Sanitario/Guia_Calidad_Agua/CD_GuiasOMS/Biblioteca/AguasResiduales/gwwuchemicals.pdf (accessed 08.09.13).
United Nations member states United Nations member states			
Principality of **Andorra**	Andorra Official Gazette (2010)	Catalan	Andorra Official Gazette, 2010. Decree approving the Regulation of contaminated soil (in Catalan). http://www.bopa.ad/bopa.nsf/c56341fced070c89c12566c700571ddd/54e68267f52e69b7c12576cf00372375?OpenDocument (accessed 16.07.13).
Republic of **Armenia**	Armenia Minister Of Health (2011)	Armenian	Armenia Minister of Health, 2011. Land and the Rules and Hygiene Requirements N2.1.7.003-10 (in Armenian). www.awhhe.am/downloads/eu_project_presentations/POPs_in_soil.doc (accessed 16.07.13).
Commonwealth of **Australia**	Australia National Environmental Protection Council (1999)	English	Australia National Environmental Protection Council, 1999. National Environment Protection (Assessment of Site Contamination) Measure 1999. http://www.comlaw.gov.au/Details/F2013C00288/Html/Volume_2 (accessed 30.07.13).
(Australia) **Australian Capital Territory**	Australia National Environmental Protection Council (1999)	English	Australia National Environmental Protection Council, 1999. National Environment Protection (Assessment of Site Contamination) Measure 1999. http://www.comlaw.gov.au/Details/F2013C00288/Html/Volume_2 (accessed 30.07.13).
(Australia) State of **Tasmania**	Tasmania Environmental Protection Authority (2012)	English	Tasmania Environmental Protection Authority, 2012. Classification and Management of Contaminated Soil for Disposal. http://epa.tas.gov.au/documents/ib105_classification_and_management_of_contaminated_soil_2012.pdf (accessed 29.07.13).
	Australia National Environmental Protection Council (1999)	English	Australia National Environmental Protection Council, 1999. National Environment Protection (Assessment of Site Contamination) Measure 1999. http://www.comlaw.gov.au/Details/F2013C00288/Html/Volume_2 (accessed 30.07.13).
(Australia) State of **New South Wales**	New South Wales Department of Environment and Conservation (2006)	English	New South Wales Department of Environment and Conservation, 2006. Guidelines for the NSW Site Auditor Scheme (2nd edition). http://www.epa.nsw.gov.au/resources/clm/auditorglines06121.pdf (accessed 29.07.13).
(Australia) State of **Northern Australia**	Australia National Environmental Protection Council (1999)	English	Australia National Environmental Protection Council, 1999. National Environment Protection (Assessment of Site Contamination) Measure 1999. http://www.comlaw.gov.au/Details/F2013C00288/Html/Volume_2 (accessed 30.07.13).
(Australia) State of **Queensland**	Australia National Environmental Protection Council (1999)	English	Australia National Environmental Protection Council, 1999. National Environment Protection (Assessment of Site Contamination) Measure 1999. http://www.comlaw.gov.au/Details/F2013C00288/Html/Volume_2 (accessed 30.07.13).

(Australia) State of **Victoria**	Victoria Environmental Protection Authority (2002)	English	Victoria Environmental Protection Authority, 2002. Victoria Government Gazette. http://www.gazette.vic.gov.au/ (accessed 12.08.13).
	Australia National Environmental Protection Council (1999)	English	Australia National Environmental Protection Council, 1999. National Environment Protection (Assessment of Site Contamination) Measure 1999. http://www.comlaw.gov.au/Details/F2013C00288/Html/Volume_2 (accessed 30.07.13).
(Australia) State of **South Australia**	Australia National Environmental Protection Council (1999)	English	Australia National Environmental Protection Council, 1999. National Environment Protection (Assessment of Site Contamination) Measure 1999. http://www.comlaw.gov.au/Details/F2013C00288/Html/Volume_2 (accessed 30.07.13).
	South Australia Environment Protection Authority (2006)	English	South Australia Environment Protection Authority, 2006. EPA Guidelines for Environmental management of on-site remediation. http://www.epa.sa.gov.au/xstd_files/Site%20contamination/Guideline/guide_remediation.pdf (accessed 23.07.13).
(Australia) State of **Western Australia**	Western Australia Department of Environment And Conservation (2010)	English	Western Australia Department of Environment and Conservation, 2010. Assessment Levels for Soil, Sediment and Water. http://www.dec.wa.gov.au/search.html?searchword=assessment%20levels%20for%20soil,%20sediment%20and&searchphrase=all (accessed 29.07.13).
Commonwealth of the **Bahamas**	Bahamas Ministry of Works And Transport (2008)	English	Bahamas Ministry of Works and Transport, 2008. SUPPLEMENTARYFINANCINGFORNEWPROVIDENCE TRANSPORTPROGRAM-BAHAMAS (BH-L1024). http://idbdocs.iadb.org/wsdocs/getdocument.aspx?docnum=1415036 (accessed 14.08.13).
Republic of **Belarus**	Belarus Ministry of Health (2004)	Belarusian	Belarus Ministry of Health, 2004. Hygienic standards 2.1.7.12-1-2004 "The list of maximum permissible concentration (MPC) and the approximate permissible concentrations (APC) of chemical substances in soil" (in Belarusian). http://minzdrav.gov.by/ru/static/acts/tehnicheskie/teksty/pochva (accessed 05.08.13).
Federative Republic of **Brazil**	Brazil Ministry of The Environment (2009)	Portuguese	Brazil Ministry of the Environment, 2009. National Council on the Environment Resolution No. 420, 28 December 2009 (in Portuguese). http://www.mma.gov.br/port/conama/legiabre.cfm?codlegi=620 (accessed 02.08.13).
(Brazil) State of **San Paolo**	Environmental Company of Sao Paolo (2005)	Portuguese	Environmental Company of Sao Paolo, 2005. Guiding Values for Soils and Groundwater in the State of San Paulo (in Portuguese). http://www.cetesb.sp.gov.br/Solo/relatorios/tabela_valores_2005.pdf (accessed 02.08.13).
Republic of **Bulgaria**	Bulgaria Ministry of Environment And Water (2008)	Bulgarian	Bulgaria Ministry of Environment and Water, 2008. Ordinance № 3 from 1 August 2008, limit values of harmful substances in the soil (in Bulgarian). http://www.moew.government.bg/?show=top&cid=388 (accessed 12.08.13).
**Canada**	Canadian Council of Ministers of The Environment (CCME) (2011)	English	Canadian Council of Ministers of the Environment (CCME), 2011. Soil Quality Guidelines for the Protection of Environmental and Human Health. http://st-ts.ccme.ca/ (accessed 14.08.13).
(Canada) Province of **Alberta**	Alberta Environment (2010)	English	Alberta Environment, 2010. Alberta Tier 1 Soil and Groundwater Remediation Guidelines (2010). http://www.environment.alberta.ca/01058.html (accessed 15.08.13).
(Canada) Province of **British Columbia**	British Columbia Regulations (2013)	English	British Columbia Regulations, 2013. Schedule 5, Environmental Management Act CONTAMINATED SITES REGULATION. http://www.bclaws.ca/EPLibraries/bclaws_new/document/LOC/freeside/--%20E%20--/Environmental%20Management%20Act%20SBC%202003%20c.%2053/05_Regulations/21_375_96%20Contaminated%20Sites%20Regulation/375_96_07.xml#Schedule5 (accessed 15.08.13).
	British Columbia Regulations (2013)	English	British Columbia Regulations, 2013. Schedule 7, Environmental Management Act CONTAMINATED SITES REGULATION. http://www.bclaws.ca/EPLibraries/bclaws_new/document/LOC/freeside/--%20E%20--/Environmental%20Management%20Act%20SBC%202003%20c.%2053/05_Regulations/21_375_96%20Contaminated%20Sites%20Regulation/375_96_09.xml#Schedule7 (accessed 15.08.13).
	British Columbia Regulations (2013)	English	British Columbia Regulations, 2013. Schedule 10, Environmental Management Act CONTAMINATED SITES REGULATION. http://www.bclaws.ca/EPLibraries/bclaws_new/document/LOC/freeside/--%20E%20--/Environmental%20Management%20Act%20SBC%202003%20c.%2053/05_Regulations/21_375_96%20Contaminated%20Sites%20Regulation/375_96_12.xml#Schedule10 (accessed 15.08.13).

(Canada) Province of **Manitoba**	Manitoba Conservation (2011), CCME (2011)	English	Manitoba Conservation, 2011. Environmental Services Criteria/Standards and Guidelines. http://www.gov.mb.ca/conservation/envprograms/contams/standards/index.html (accessed 15.08.13).
(Canada) Province of **Newfoundland and Labrador**	Environment and Conservation, Government of Newfoundland and Labrador (2005)	English	Environment and Conservation, Government of Newfoundland and Labrador, 2005. Guidance Document for the Management of Impacted Sites. http://www.env.gov.nl.ca/env/env_protection/ics/guidancedocv.1.01external.pdf (accessed 15.08.13).
(Canada) **Northwest Territories**	Northwest Territories Department of Environment and Natural Resources (2003)	English	Northwest Territories Department of Environment and Natural Resources, 2003. Environmental Guideline for Contaminated Site Remediation. http://www.enr.gov.nt.ca/_live/documents/content/siteremediation.pdf (accessed 19.08.13).
Canada **Nova Scotia**	Nova Scotia Environment (2013)	English	Nova Scotia Environment, 2013. Table 1A Tier 1 Environmental Quality Standards for Soil at a Potable Site. http://www.novascotia.ca/nse/contaminatedsites/docs/Table_1A_Tier1_EQS_for_Soil_Potable_Site.pdf (accessed 15.08.13).
			Nova Scotia Environment, 2013. Table 1B Tier 1 Environmental Quality Standards for Soil at a Non-Potable Site. http://www.novascotia.ca/nse/contaminatedsites/docs/Table_1B_Tier1_EQS_for_Soil_Non-Potable_Site.pdf (accessed 15.08.13).
(Canada) **Nunavut**	Department of Environment, Government of Nunavut (2009)	English	Department of Environment, Government of Nunavut, 2009. Environmental Guideline for Contaminated Site Remediation. http://env.gov.nu.ca/sites/default/files/Guideline%20Contaminated%20Site%20Remediation.pdf (accessed 19.08.13).
(Canada) Province of **Ontario**	Ontario Ministry of The Environment (2011)	English	Ontario Ministry of the Environment, 2011. Soil, Ground Water and Sediment Standards for Use Under Part XV.1 of the Ontario Protection Act.PIBS# 7382e01. http://www.ene.gov.on.ca/environment/en/subject/brownfields/STDPROD_075745.html (accessed 19.08.13).
(Canada) Province of **Prince Edward Island**	Prince Edward Island Environment, Energy and Forestry (2010)	English	Prince Edward Island Environment, Energy and Forestry, 2010. Chapter E-9 Environmental Protection Act Contaminated Sites Registry Regulations. http://www.gov.pe.ca/law/regulations/pdf/E&09-03.pdf (accessed 19.08.13).
(Canada) Province of **Quebec**	Quebec Ministry of Sustainable Development, Environment and Parks (1998)	French	Quebec Ministry of Sustainable Development, Environment and Parks, 1998. Appendix 2: Generic criteria for soils and groundwater, Policy of soil protection and remediation of contaminated land (in French). http://www.mddep.gouv.qc.ca/sol/terrains/politique/annexe_2_tableau_1.htm (accessed 19.08.13).
(Canada) **Yukon**	Yukon Regulations (2002)	English	Yukon Regulations, 2002. O.I.C. 2002/171 Environment Act. http://www.gov.yk.ca/legislation/regs/oic2002_171.pdf (accessed 19.08.13).
People's Republic of **China**	Peoples Republic of China (1995)	Chinese	Peoples Republic of China, 1995. Environmental quality standard for soils (GB 15618-1995 1996-03-01) (in Chinese). http://kjs.mep.gov.cn/hjbhbz/bzwb/trhj/trhjzlbz/199603/t19960301_82028.htm (accessed 20.08.13).
	People's Republic of China Ministry of Environmental Protection (2006)	Chinese	People's Republic of China Ministry of Environmental Protection, 2006. National Standard for edible agriculture product (in Chinese). http://kjs.mep.gov.cn/hjbhbz/bzwb/trhj/trhjzlbz/200412/t20041229_63466.htm (accessed 20.08.13).
Peoples Republic of China (**Beijing**)	Beijing Municipal Environmental Protection Bureau (2011)	Chinese	Beijing Municipal Environmental Protection Bureau, 2011. Environmental Risk Assessment Screening venues soil values (in Chinese). http://www.bjepb.gov.cn/ (accessed 20.08.13).
Republic of **Costa Rica**	Ministry of Health (2011)	Spanish	Ministry of Health, 2011. No.-S-MINAET-MAG The President of the Republic Health Minister, Minister Of Environment, Energy and Telecommunications, and the Minister of Agriculture and Livestock (in Spanish). http://www.amcham.co.cr/newsletters/201103/contaminantes.pdf (accessed 21.08.13).
(**Croatia**) Agricultural Uni. Of Zagreb	Agricultural University of Zagreb (2008)	Croatian	Agriculture University of Zagreb, 2008. Preparation of Croatian Soil Monitoring Pilot Project (LIFE05 TCY/CRO/000105 Final Report) (in Croatian). http://www.azo.hr/Projects05 (accessed 21.08.13).
**Czech** Republic	Czech Republic Ministry of The Environment (1994)	English	Czech Republic Ministry of Environment, 1994. Decree 13/1994 of the Ministry of the Environment of the Czech Republic Specifying Some Details of Agricultural Land Resources Protection. http://faolex.fao.org/docs/pdf/cze62400E.pdf (accessed 22.08.13).

	European Commission (2007)	English	European Commission, 2008. Priority Substances and Certain other Pollutants (According to AnnexII of theDirective2008/105/EC). http://ec.europa.eu/environment/water/water-framework/priority_substances.htm (accessed 22.08.13).
Kingdom of **Denmark**	Danish Environmental Protection Agency (2010)	Danish	Danish Environmental Protection Agency, 2010. List of quality criteria in the contaminated soil and quality of drinking water (in Danish). http://www.mst.dk/NR/rdonlyres/0AB0AF23-4BD6-4901-BCD9-43F6F6FD6FC1/124958/Kvalitetskriterierjord_og_drikkevandfinaljuniogjul.pdf (accessed 22.08.13).
Republic of **Ecuador**	Ecuador Ministry of the Environment (2004)	Spanish	Ecuador Ministry of Environment, 2004. Environmental Quality Standard of Soil Resources and Criteria for Contaminated Soil (in Spanish). http://faolex.fao.org/docs/pdf/ecu112181.pdf (accessed 23.08.13).
Republic of **Estonia**	Estonia Ministry of the Environment (2004)	Estonian	Estonia Ministry of the Environment, 2004. In Soil and Groundwater Concentration Limits for of Hazardous Substances (No 12, Adopted 04/02/2004 RTL 2004, 40,662) (in Estonian). https://www.riigiteataja.ee/akt/961568 (accessed 23.08.13).
Republic of **Finland**	Finland Ministry Of The Environment (2007)	Finish	Finland Ministry of the Environment, 2007. Government Regulation of Soil Contamination and Cleaning Needs Assessment (in Finish). http://www.finlex.fi/data/sdliite/liite/5382.pdf (accessed 22.08.13).
French Republic (**France**)	European Commission (2007)	English	European Union, 2010. Regulations Commission Regulation (EU) No 756/2010. http://eur-lex.europa.eu/LexUriServ/LexUriServ.do?uri=OJ:L:2010:223:0020:0028:EN:PDF (accessed 09.09.13).
**Georgia**	Georgia Minister of Environment and Minister of Natural Resources (2006)	Georgian	Georgia Ministry of Environment and Minister of Natural Resources, 2006. Decree N538 e For the Calculation of Damage to the Environment. Appendix 4-Pollution of Land Quality Assessment Table (in Georgian). http://www.cenn.org/wssl/programs/zianis_daangariSebis_metodika_Methodology_Environmental_Damage.pdf (accessed 03.09.13).
	Minister of Health, Labor and Social Affairs (2001)	Georgian	Minister of Health, labor and social affairs, 2001. Environmental concentrations of pesticides approved hygiene standards (in Georgian). http://medportal.ge/mohsrv/lawfiles/781.htm (accessed 03.09.13).
Federal Republic of **Germany**	German Federal Ministry of the Environment, Nature Conservation and Nuclear Safety (1999)	German	German Federal Ministry of the Environment, Nature Conservation and Nuclear Safety, 1999. Federal Soil Protection and Contaminated Sites Regulation (BBODSCHG) (in German). http://www.bmu.de/fileadmin/bmu-import/files/pdfs/allgemein/application/pdf/bbodschv_uk.pdf (accessed 23.08.13).
**Hungary**	Hungary Ministry of the Environment (2000)	Hungarian	Hungary Ministry of the Environment, 2000. (June 2) Ministry of Environment, Ministry of Health, Ministry of Agriculture, Ministry of Transport co-regulation of groundwater limit values and quality necessary to protect the geological (in Hungarian). http://www.kvvm.hu/szakmai/karmentes/jogszab/jogszab02/tartalom2.htm (accessed 26.08.13).
Republic of **Italy**	Italy National Institute of Health (2006)	Italian	Italy National Institute of Health, 2006. Official Gazette no. 293 15-12-1999 (in Italian). http://gazzette.comune.jesi.an.it/293-99/supll218.htm (accessed 26.08.13).
(Italy) **Lombardi** Region	Tazzioli (1999)	Italian	Tazzioli, R., 1999. The Quality Standards Applicable for the Protection of Soil Pollution (in Italian). http://www.sindar.it/Editoriale/Allegati/Argomenti/suoli1.pdf (accessed 25.08.10).
(Italy) **Piedmont** Region	Tazzioli (1999)	Italian	Tazzioli, R., 1999. The Quality Standards Applicable for the Protection of Soil Pollution (in Italian). http://www.sindar.it/Editoriale/Allegati/Argomenti/suoli1.pdf (accessed 25.08.10).
(Italy) **Emili Romana** Region	Tazzioli (1999)	Italian	Tazzioli, R., 1999. The Quality Standards Applicable for the Protection of Soil Pollution (in Italian). http://www.sindar.it/Editoriale/Allegati/Argomenti/suoli1.pdf (accessed 25.08.10).
(Italy) **Liguria** Region	Tazzioli (1999)	Italian	Tazzioli, R., 1999. The Quality Standards Applicable for the Protection of Soil Pollution (in Italian). http://www.sindar.it/Editoriale/Allegati/Argomenti/suoli1.pdf (accessed 25.08.10).
Republic of **Latvia**	Latvia Cabinet of Ministers (2005)	Latvian	Latvia Cabinet of Ministers, 2005. Terms of soil and soil quality standards (in Latvian). http://likumi.lv/doc.php?id=120072 (accessed 27.08.13).
Republic of **Lithuania**	Lithuania Ministry of the Environment (2008)	Lithuanian	Contaminated Sites Management Environmental Protection (April 30, 2008) (in Lithuanian). http://tar.tic.lt/Default.aspx?id¼2&item¼results&aktoid¼43AD7376-25A2-47B1-9378-554EE563D95B (accessed 27.08.13).

**Malaysia**	Malaysia Environment Protection Department (2009)	English	Malaysia Department of Environment, 2009. Contaminated Land Management and Control Guidelines No. 1: Malaysian Recommended Site Screening Levels for Contaminated Land. http://www.doe.gov.my/portal/wp-content/uploads/Contaminated-Land-Management-and-Control-Guidelines-No-1_Malaysian-Recommended-Site-Screening-Levels-for-Contaminated-Land.pdf (accessed 28.08.13).
Republic of **Moldova**	Moldova Ministry of Ecology and Natural Resources (2004)	Romanian	Moldova Ministry of Ecology and Natural Resources, 2004. NOTE Nr. 383 of 08.08.2004 Instructions injury assessment of soil resources (in Romanian). http://lex.justice.md/index.php?action=view&view=doc&lang=1&id=310719 (accessed 30.08.13).
Kingdom of the **Netherlands**	Netherlands National Institute for Public Health and the Environment (2006)	English	Netherlands National Institute for Public Health and the Environment (2006). Preliminary draft of the Soil Quality Regulation. http://www.rwsleefomgeving.nl/publish/pages/92483/soil_quality_regulation_first_notification_24_311345.pdf (accessed 02.09.2013).
	Netherlands Ministry of Economic Affairs, Agriculture and Innovation (2006)	English	Netherlands National Institute for Public Health and Environment, 2001. Technical Evaluation of the Intervention Values for Soil/Sediment and Groundwater, RIVM Report 711701 023. http://www.rivm.nl/bibliotheek/rapporten/711701023.pdf (accessed 01.09.13).
	Netherlands National Institute for Public Health and the Environment (2009)	English	Netherlands National Institute for Public Health and the Environment, 2009. Soil Remediation Circular 2009 (in Dutch). http://www.esdat.com.au/Environmental%20Standards/Dutch/ENGELSE%20versie%20circulaire%20Bodemsanering%202009.pdf (accessed 02.09.2013).
**New Zealand**	New Zealand Ministry of the Environment (2012)	English	New Zealand Ministry of the Environment, 2012. Users' Guide National Environmental Standard for Assessing and Managing Contaminants in Soil to Protect Human Health. http://www.mfe.govt.nz/publications/rma/users-guide-nes-for-assessing-managing-contaminants-in-soil/guide-nes-for-assessing-managing-contaminants-in-soil.pdf (accessed 06.09.13).
	New Zealand Ministry of the Environment	English	New Zealand Ministry of the Environment. Proposed National Environmental Standard for Assessing and Managing Contaminants in Soil. http://www.mfe.govt.nz/publications/rma/proposed-nes-managing-contaminants-in-soil/page8.html (accessed 06.09.13).
	New Zealand Ministry of the Environment (1997)	English	New Zealand Ministry of the Environment, 1997. Health and Environmental Guidelines for Selected Timber Treatment Chemicals. http://www.mfe.govt.nz/publications/hazardous/timber-guide-jun97/timber-guide-jun97.pdf (accessed 06.09.13).
	New Zealand Ministry of the Environment (2006)	English	New Zealand Ministry of the Environment, 2006. Identifying, Investigating and Managing Risks Associated with Former Sheep-dip Sites. http://www.mfe.govt.nz/publications/hazardous/risks-former-sheep-dip-sites-nov06/risks-former-sheep-dip-sites-nov06.pdf (accessed 06.09.13).
	New Zealand Ministry of the Environment (2011)	English	New Zealand Ministry of the Environment, 2011. Methodology for Deriving Standards for Contaminants in Soil to Protect Human Health. http://www.mfe.govt.nz/publications/hazardous/deriving-standards-for-contaminants-in-soil/methodology-for-deriving-standards-for-contaminants-in-soil.pdf (accessed 06.09.13).
	New Zealand Ministry of the Environment (2013)	English	New Zealand Ministry of the Environment, 2013. Environmental Guideline Value (EGV). https://www.mfe.govt.nz/issues/managing-environmental-risks/contaminated-land/managing/egv-database.html (accessed 23.04.14).
(New Zealand) **Auckland City Council**	Cavanagh (2006)	English	Cavanagh, J.E., 2006. Comparison of Soil Guideline Values in New Zealand and Their Derivations. http://ecan.govt.nz/publications/Reports/ComparisonofSoilGuidelineValuesinNewZealand.pdf (accessed 06.09.13).
(New Zealand) **Auckland Regional Council**	Cavanagh (2006)	English	Cavanagh, J.E., 2006. Comparison of Soil Guideline Values in New Zealand and Their Derivations. http://ecan.govt.nz/publications/Reports/ComparisonofSoilGuidelineValuesinNewZealand.pdf (accessed 06.09.13).
(New Zealand) **Bay of Plenty**	Cavanagh (2006)	English	Cavanagh, J.E., 2006. Comparison of Soil Guideline Values in New Zealand and Their Derivations. http://ecan.govt.nz/publications/Reports/ComparisonofSoilGuidelineValuesinNewZealand.pdf (accessed 06.09.13).

(New Zealand) **Hastings District Council**	Cavanagh (2006)	English	Cavanagh, J.E., 2006. Comparison of Soil Guideline Values in New Zealand and Their Derivations. http://ecan.govt.nz/publications/Reports/ComparisonofSoilGuidelineValuesinNewZealand.pdf (accessed 06.09.13).
(New Zealand) **Tasmasn District Council**	Cavanagh (2006)	English	Cavanagh, J.E., 2006. Comparison of Soil Guideline Values in New Zealand and Their Derivations. http://ecan.govt.nz/publications/Reports/ComparisonofSoilGuidelineValuesinNewZealand.pdf (accessed 06.09.13).
(New Zealand) **Waikato Region**	Cavanagh (2006)	English	Cavanagh, J.E., 2006. Comparison of Soil Guideline Values in New Zealand and Their Derivations. http://ecan.govt.nz/publications/Reports/ComparisonofSoilGuidelineValuesinNewZealand.pdf (accessed 06.09.13).
Kingdom of **Norway**	Norwegian Pollution Control Authority (1999)	English	Norwegian Pollution Control Authority, 1999. Guidelines for the risk assessment of contaminated sites. http://www.miljodirektoratet.no/publikasjoner/luft/1691/ta1691.pdf (accessed 05.09.13).
Republic of **Panama**	Panama Ministry of Economy and Finance (2009)	Spanish	Panama Ministry of Economy and Finance, 2009. Executive Order 2-Which Sets the Standard for Soil Environmental Quality for Different Applications (in Spanish). http://www.anam.gob.pa/images/stories/normas_ambientalesdiproca/Decreto_Ejecutivos_No_2.pdf (accessed 05.09.13).
Republic of **Peru**	Peru Ministry of Environment (2013)	Spanish	Peru Ministry of Environment, 2013. Environmental Quality Standards (ECA) (in Spanish). http://www.minam.gob.pe/index.php?option=com_content&view=article&id=102:est (accessed 05.09.13).
Republic of **Poland**	Poland Minister of the Environment (2002)	Polish	Poland Minister of the Environment, 2002. Dz.U.2002.165.1359-On Quality Standards for Soil and Land (4 Oct. 2002) (in Polish). http://www.abc.com.pl/du-akt/-/akt/dz-u-02-165-1359 (accessed 05.09.13).
Republic of **Portugal**	Ontario Ministry of Environment And Energy (1997)	Portuguese and English	Ontario Ministry of Environment and Energy, 1997. Recommendations of the Institute of Waste (in Portuguese). http://ia600409.us.archive.org/9/items/guidelineforusea00ontauoft/guidelineforusea00ontauoft.pdf (accessed 05.09.13).
**Qatar**	Qatar Ministry of Environment (2007)	Arabic	Qatar Ministry of Environment, 2007. Ministry of Environment Implements a Program on Soil Quality in Qatar for Three Years (in Arabic). http://www.moe.gov.qa/English/News/Local/Pages/Ministry-of-Environment-Implements-a-Program-on-Soil-Quality-in-Qatar-for-Three-Years.aspx (accessed 06.09.13).
**Romania**	Romania Ministry of Waters, Forests and Environmental Protection (1997)	Romanian and English	Romanian Ministry of Water Forests and Environmental Protection, 1997. Approving the Regulation on the Assessment of Environmental Pollution. Official Gazette of Romania No. 303bis/06.11.1997 (in Romanian). http://www.moficial.ro/1997/303bis.pdf (accessed 06.12.11).
Russian Federation (**Russia**)	Russian State Construction Code (1997)	Russian	Russian State Construction Code, 1997. P 11-102-97 Engineering and Environmental Surveys for Construction. Appendix B. Criteria for Environmental Assessment of Pollution of Soil and Groundwater in Residential Areas (in Russian). http://ohranatruda.ru/ot_biblio/normativ/data_normativ/5/5155/index.php#i643158 (accessed 04.09.13).
	Russian Ministry of Environment and Natural Resources (1993)	Russian	Russian Ministry of Environment and Natural Resources, 1993. Table 2 and Table 3, The procedure for determining the extent of damage from land contamination by chemicals (in Russian). http://www.dioxin.ru/doc/N04-25.61-5678.htm (accessed 04.09.13).
(Russia) City of **Moscow**	Moscow Government (2004)	Russian	Moscow Government, 2004. Improving the Quality of Soils in the City of Moscow (in Russian). http://www.ecoekspert.ru/art/rcent/44.html (accessed 05.09.13).
(Russia) Republic of **Tatarstan**	Republic of Tatarstan Ministry of Environment and Natural Resources (2002)	Russian	Republic of Tatarstan Minister of Ecology and Natural Resources, 2002. Order-Sizing Penalties for Any Damage to Land and Vegetation (No. 314) (in Russian). eco.tatar.ru/rus/file/pub/pub_8462.doc (accessed 05.09.13).
Republic of **Serbia**	Serbia Ministry of Environment And Spatial Planning (1994)	English	Serbia Ministry of Environment and Spatial Planning, 1994. Rulebook on the Allowed Quantities of Hazardous and Harmful Substances in the Ground and on the Methods for Their Examination. Official Herald of the Republic of Serbia, No. 23/94. http://www-wds.worldbank.org/external/default/WDSContentServer/WDSP/IB/2005/04/21/000011823_20050421141937/Rendered/INDEX/E11400VOL.06.txt (accessed 08.08.11).

Republic of **Singapore**	Singapore National Environmental Agency (2010)	English	Singapore National Environmental Agency, 2010. Guideline on Environmental Baseline Study. http://www.jtc.gov.sg/Documents/EBSGuidelines.pdf (accessed 03.09.13).
Slovak Republic (**Slovakia**)	Slovakia Ministry of Agriculture (2004)	Slovak	Slovakia Ministry of Agriculture, 2004. Act No. 220/2004 Coll. On the Protection and Use of the Agricultural Soil (in Slovak). http://www.podnemapy.sk/portal/verejnost/konsolidacia/z_220_2004.pdf (accessed 03.09.13).
Republic of **Slovenia**	Slovenia Ministry of Environment and Spatial Planning (1996)	Slovenian	Slovenia Ministry of Environment and Spatial Planning, 1996. The Limit, Warning and Critical Levels of Hazardous Substances in Soil (in Slovenian) Official Gazette of the Republic of Slovenia 68/1996, 5769-5843 (Nov. 29, 1996). http://www.uradni-list.si/_pdf/1996/Ur/u1996068.pdf (accessed 03.09.13).
Republic of **South Africa**	South Africa Department of Environmental Affairs (2010)	English	South Africa Department of Environmental Affairs, 2010. Government Gazette, 23 August 2013, National Norms and Standards for the Assessment of Waste for Landfill Disposal. https://www.environment.gov.za/sites/default/files/legislations/nemwa59of2008_norms_standards_forassessment.pdf (accessed 03.09.13).
Kingdom of **Spain**	Spain Ministry of the Presidency (2005)	Spanish	Spain Ministry of the Presidency, 2005. Royal Decree 9/2005, of 14 January, the ratio of activities set potentially contaminating soil and criteria and standards for the declaration of contaminated soils (in Spanish). http://www.boe.es/boe/dias/2005/01/18/pdfs/A01833-01843.pdf (accessed 04.09.13).
(Spain) Autonomous Community of **Andalusia**	Andalusia Ministry of Environment (2006)	Spanish	Andalusia Ministry of Environment, 2006. Evaluation of Quality Soil and Levels of Generic Reference (in Spanish). http://www.juntadeandalucia.es/medioambiente/web/Bloques_Tematicos/Estado_Y_Calidad_De_Los_Recursos_Naturales/Suelo/Contaminacion_pdf/Evaluacion.pdf (accessed 04.09.13).
(Spain) Autonomous Community of **Aragon**	Government of Aragon (2005)	Spanish	Government of Aragon, 2005. Implementation of real decree 9/2005 DE contaminated soil in Aragon (in Spanish). http://www.camarazaragoza.com/medioambiente/docs/bibliocamara/bibliocamara165.pdf (accessed 04.12.13).
(Spain) Principality of **Asturias**	The Government of the Principality of Asturias (2005)	Asturianu	The Government of the Principality of Asturias, 2005. A Preliminary Report on The Situation of Contaminated Soils (in Asturianu). http://www.asturia.es/porta;/site/Asturias/menuitem.46a76b28f520ecaaf18e90bbb30a0a0/?vgnextoid=90f3c159b5d4f010VgnVCM100000b0030a0aRCRD&i18n.http.lang=es (accessed 07.12.13).
(Spain) Autonomous Community **Balearic Islands**	Ministry of Agriculture, Environment and Territory of Balearic Islands (2013)	Catalan	Ministry of Agriculture, Environment and Territory of Balearic Islands, (2013). Contaminated soil (in Catalan). http://www.caib.es/govern/sac/visor_proc.do?codi=507019&lang=en (accessed 07.12.13).
(Spain) **Basque** Country	Basque Government, Department of Environment, Planning, Agriculture and Fisheries (2005)	Basque	Basque Government Department of Environment, Planning, Agriculture and Fisheries, 2005. Law 1/2005 for the Prevention and Correction of Soil Contamination (in Basque), Official Gazette of Basque Country No 32, Feb.16, 2005. http://www.euskadi.net/bopv2/datos/2005/02/0500661a.pdf (accessed 04.09.13).
(Spain) Autonomous Community of **Canary Islands**	Government of Canary Islands (2007)	Spanish	Government of Canary Islands, 2007. Annex I, Natural Sciences, Official Bulletin No. Canarias. 113, Thursday June 7, 2007 (in Spanish). http://www.gobcan.es/boc/2007/113/boc-2007-113-anexo-12892-13233.pdf (accessed 07.12.13).
(Spain) Autonomous Community of **Cantabria**	Government of Cantabria (2006)	Spanish	Government of Cantabria, 2006. Establishing the legal regime regulating soil contaminated in Cantabria and creates Soil Stock Contaminated (in Spanish). https://www.netenvira.com/webnormas/webnormas/noticias/proyecto-de-decreto-de-suelos-contaminados-en-cantabria_3466_966_4137_0_1_in.html (accessed 07.12.13).
(Spain) Autonomous Community of **Castile and Leon**	Government of Castile And Leon	Spanish	Government of Castile and Leon. Progress Report CONTAMINATED SOIL (in Spanish). https://www.tramitacastillayleon.jcyl.es/web/jcyl/AdministracionElectronica/es/Plantilla100DetalleFeed/1251181050732/Tramite/1277228074975/Tramite (accessed 07.12.13).
(Spain) Autonomous Community of **Castile La Mancha**	Jiménez Ballesta Et Al. (2010)	Spanish	R. Jiménez Ballesta1, P. Conde Bueno2, J.A. Martín Rubí2, R. García Giménez, 2010. Geochemical background levels and influence of the geological setting on pedogeochemical baseline concentrations of trace elements in selected soils of Castilla-La Mancha (Spain) http://webcache.googleusercontent.com/search?q=cache:e1fp7nsyNloJ:estudiosgeol.revistas.csic.es/index.php/estudiosgeol/article/download/800/828+&cd=1&hl=en&ct=clnk&gl=us (accessed 07.12.13).

(Spain) Autonomous Community of **Catalonia**	Waste Agency of Catalonia (2005)	Catalan	Waste Agency of Catalonia, 2005. Royal Decree 9/2005, of 14 January, on the list of potentially soil polluting activities, and the criteria and standards for the declaration of contaminated soils. http://www20.gencat.cat/portal/site/arc/menuitem.60fb2478680e61fd624a1d25b0c0e1a0/?vgnextoid=795d0431b17d6210VgnVCM1000008d0c1e0aRCRD&vgnextchannel=795d0431b17d6210VgnVCM1000008d0c1e0aRCRD&vgnextfmt=default (accessed 07.12.13).
	Andalusia Ministry of Environment (2006)	Spanish	Andalusia Ministry of Environment, 2006. Evaluation of Quality Soil and Levels of Generic Reference (in Spanish). http://www.juntadeandalucia.es/medioambiente/web/Bloques_Tematicos/Estado_Y_Calidad_De_Los_Recursos_Naturales/Suelo/Contaminacion_pdf/Evaluacion.pdf (accessed 04.09.13).
(Spain) Autonomous City of **Ceuta**	Official Portal of Ceuta (2013)	Spanish	Official Portal of Ceuta, 2013. Official Gazette, CITY Ceuta, Management And Administration: CITY PALACE - Archive Official Gazette City Ceuta Management and Administration: CITY PALACE - Archive Number 5,307 (in Spanish). http://www.ceuta.es/ceuta/documentos/finish/1489-octubre/7935-bocce-5307-25-10-2013 (accessed 05.12.13).
(Spain) Autonomous Community of **Extremadura**	Ministry of Agriculture and Rural Development (2010)	Spanish	Ministry of Agriculture and Rural Development, 2010. ORDER of August 3, 2010 approving the application of regulated legal compliance and management requirements are established and good agricultural and environmental conditions in the Community Autónoma de Extremadura (2010050248) (in Spanish). http://doe.juntaex.es/pdfs/doe/2010/1540o/10050248.pdf (accessed 07.12.13).
(Spain) Autonomous Community of **Galicia**	Ministry of Environment and Sustainable Development of Galicia (2009)	Galician	Ministry of Environment and Sustainable Development of Galicia, 2009. DECREE 59/2009, of 26 February, on the traceability of waste is regulated (in Spanish). http://www.xunta.es/dog/Publicados/2009/20090324/Anuncio10CC2_es.html (accessed 07.12.13).
	Andalusia Ministry of Environment (2006)	Spanish	Andalusia Ministry of Environment, 2006. Evaluation of Quality Soil and Levels of Generic Reference (in Spanish). http://www.juntadeandalucia.es/medioambiente/web/Bloques_Tematicos/Estado_Y_Calidad_De_Los_Recursos_Naturales/Suelo/Contaminacion_pdf/Evaluacion.pdf (accessed 04.09.13).
(Spain) Autonomous Community of **La Rioja**	Government of La Rioja (2007)	Spanish	Government of La Rioja, 2007. Generic reference levels (NGR) NGR for metals and protection of human health (in Spanish). http://www.larioja.org/npRioja/default/defaultpage.jsp?idtab=449537 (accessed 07.12.13).
(Spain) Autonomous Community of **Madrid**	Spain Ministry of the Presidency (2005)	Spanish	Ministry of Environment and Planning, 2007. ORDER 761/2007 of 2 April, the Minister of the Environment and Planning, by amending Order 2770/2006 of 11 August, by which generic reference levels of heavy metals and other trace elements in contaminated soils down Madrid (in Spanish). http://www.madrid.org/cs/Satellite?blobcol=urldata&blobheader=application%2Fpdf&blobkey=id&blobtable=MungoBlobs&blobwhere=1158627540041&ssbinary=true (accessed 07.12.13).
(Spain) Autonomous City of **Melilla**	Ministry of Environment of the Autonomous City of Melilla (Undated)	Spanish	Ministry of Environment of the Autonomous City of Melilla, undated. Contaminated Soil (in Spanish). http://www.melillamedioambiente.com//index.php?option=com_content&task=view&id=201&Itemid=32 (accessed 05.12.13).
(Spain) Region of **Murcia**	Government of Region of Murcia (2011)	Spanish	Government of Region of Murcia, 2011. 2025 - Declaration of contaminated soils (in Spanish). http://www.carm.es/web/pagina?IDCONTENIDO=2025&IDTIPO=240&NOMBRECANAL=Calidad+del+Aire+y+Protecci%F3n+de+la+Atm%F3sfera&RASTRO=c643$m3063,3283 (accessed 04.12.13).
(Spain) Autonomous Community of **Navarra**	Department of Rural Development, Environment and Local Government (Undated)	Basque	Department of Rural Development, Environment and Local Government, undated. State Legislation of Navarra. http://www.navarra.es/home_es/Gobierno+de+Navarra/Organigrama/Los+departamentos/Desarrollo+Rural+Industria+Empleo+y+Medio+Ambiente/Acciones/Planes+especificos/Acciones+medio+ambiente/Informacion+ambiental/Elementos/Suelos/Legislacion/Estatal.htm (accessed 13.12.13).
(Spain) Autonomous Community of **Valencia**	Generalistat Valencian Regional Ministry of Infrastructure, Planning and the Environment (2007)	Catalan	Generalistat Valencian Regional Ministry of Infrastructure, Planning and the Environment, 2007. Floor Guide Contaminated (in Spanish). http://www.cma.gva.es/contenidoHtmlArea/contenido/52721/cas/Guia_suelos.pdf (accessed 07.12.13).
United Republic of **Tanzania**	Tanzanian Bureau of Standards (2007)	English	Tanzania Bureau of Standards, 2007. Soil Quality e Limits for Soil Contaminants in Habitat and Agriculture (TSZ 972). http://www.tbs.go.tz/standards/index.php/tbs/tzs_972/ (accessed 04.04.12).

Kingdom of **Thailand**	Thailand Ministry of Natural Resources And Environment (2004)	English	Thailand Ministry of Natural Resources and Environment, 2004. Soil Quality Standards for Habitat and Agriculture. http://www.pcd.go.th/info_serv/en_reg_std_soil01.html#s1 (accessed 07.09.13).
Republic of **Turkey**	Turkey Ministry of Environment And Forestry (2001)	Turkish	Turkey Ministry of Environment and Forestry, 2001. Soil Pollution Control Regulation (in Turkish), Official Gazette of Republic of Turkey No. 24609 (10/12/2001). http://www.bcm.org.tr/pdf/toprak_kir_yon.doc (accessed 07.09.13).
**Ukraine**	Ministry of Health of Ukraine (2001)	Ukrainian	Ministry of Health of Ukraine, 2001. Appendix 2 and 3, State sanitary rules and norms DSanPiN 8.8.1.2.3.4-000-2001 (in Ukrainian). http://www.uazakon.com/documents/date_8m/pg_grwssr/index.htm (accessed 07.09.13).
(United Kingdom) Anglian Water Services	Anglian Water Services Ltd. (2010)	English	Anglian Water Services Ltd, 2010. Information for Developers about Contaminated Land and Ground Condition Assessment. http://www.anglianwater.co.uk/_assets/media/Contaminated-sites.pdf (accessed 07.09.13).
(United Kingdom) **White Young Green Environmental Ltd**	White Young Green Environmental Ltd. (2008)	English	White Young Green Environmental Ltd, 2008. Sainsburys Supermarkets Ltd Waterlooville Ground Condition Assessment Report, Appendix DeWYGEs TVSs. http://www5.havant.gov.uk/AcolaidIntegration/GetDocumentContent.aspx?documentId=195262 (accessed 07.09.13).
Republic of **Uzbekistan**	Head of State Health Officer of The Republic of Uzbekistan (2005)	Russian	Head of State health officer of the Republic of Uzbekistan, 2005. Sanitary Maximum Permissible Concentration (MPC) and the Indicative Permissible Concentration (APC) of Exogenous Harmful Substances in Soil 2005 (in Russian). http://www.lex.uz/Pages/GetAct.aspx?lact_id=1905543&search_text= (accessed 10.10.13).
Socialist Republic of **Vietnam**	Republic of Vietnam (2008)	Vietnamese	Republic of Vietnam, 2008. National technical regulation on the pesticide residues in the soils (in Vietnamese). http://vea.gov.vn/vn/vanbanphapquy/tcmt/Documents/Quy%20chuan%20ky%20thuat%20quoc%20gia%20ve%20MT/QC_HCBVTV%2004doc.pdf (accessed 08.09.13).
	Republic of Vietnam (1995)	Vietnamese	Republic of Vietnam, 1995. TCVN5941-1995 (in Vietnamese). http://thuvienkhcn.vinhlong.gov.vn/tailieukhcn/data/TieuChuantoanvan/MOITRUONG/TCVN_5941_95.pdf (accessed 22.11.13).
Jurisdictions other than United Nations member states			
**Union of Soviet Socialist Republics** (USSR)	The State Standard of the Ussr (1983)	Russian	The State Standard of the USSR, 1983. Soil: Classification of Chemicals for Pollution Control. 17.4.1.02-83 Standard (in Russian). http://zakonbase.ru/content/base/66884 (accessed 04.09.13).
	State Medical Officer of the USSR (1982)	Russian	State Medical Officer of the USSR, 1982. Guidelines in hygienic substantiation MPC Chemical Substances in the soil. August 5, 1982 N 2609-82 (in Russian). http://www.libussr.ru/doc_ussr/usr_11234.htm (accessed 04.09.13).
	The USSR Ministry (1991)	Russian	The USSR Ministry, 1991. The list of maximum permissible concentration (MPC) of chemicals in the soil № 6229-91. Approved. USSR Ministry of 19/11/91 (in Russian). http://pravo.levonevsky.org/baza/soviet/sssr0012.htm (accessed 04.09.13).
	Ministry Of Health of the USSR (1980)	Russian	Ministry of Health of the USSR, 1980. Maximum allowable concentrations of chemicals in the soil (MPC) (in Russian). http://www.libussr.ru/doc_ussr/usr_10508.htm (accessed 04.09.13).

Table S2. Worldwide pesticide drinking water regulatory jurisdictions references and dates accessed.

Jurisdictions	Sources	Languages	Websites References
			Multinational organizations
**East Africa Community**	East African Community (2012)	English	East African Community, 2012. Potable Water Specification. www.eac-quality.net/fileadmin/eac_quality/user_documents/3_pdf/DEAS_12_POTABLE_WATER_2012.docx+&cd=1&hl=en&ct=clnk&gl=us (Accessed September 17. 2014).
**European Union**	European Union (1998)	English	European Union, 1998. On the quality of water intended for human consumption. http://eur-lex.europa.eu/legal-content/EN/TXT/PDF/?uri=CELEX:31998L0083&from=EN (Accessed August 18. 2014).
**Gulf Standardization Organization**	Gulf Standardization Organization (2012)	Arabic and English	Gulf Standardization Organization, 2012. GSO5/ FDS / 2012 Bottled drinking water (In Arabic and English). http://www.khcnpy.gov.vn/ChicucTDC/uploads/14_1771_00_x.pdf (Accessed September 4. 2014).
**World Health Organization**	World Health Organization (2011)	English	World Health Organization, 2011. Guidelines for drinking-water quality fourth edition. http://www.who.int/water_sanitation_health/publications/2011/dwq_chapters/en/ (Accessed April 5. 2014).
			United Nations member states
Republic of **Albania**	Albania Institute for European Environmental Policy (2007)	Albanian	Albania Institute for European Environmental Policy, 2007. Drinking water quality standards (STASH 3904:1997 ICS 13060) (In Albanian). http://ec.europa.eu/environment/archives/international_issues/pdf/report_albania.pdf (Accessed September 10. 2014).
Principality of **Andorra**	Andorra Official Gazette (1999)	Catalan	Andorra Official Gazette, 1999. Regulation for technical healthcare delivery and quality control of drinking water for human (in Catalan).https://www.bopa.ad/bopa/011044/Pagines/19D62.aspx (Accessed August. 14. 2014).
**Antigua** and **Barbuda**	Environmental Solutions Antigua Limited (2008)	English	Environmental Solutions Antigua Limited, 2008. Water Quality Guidelines for Antigua & Barbuda. http://gefantigua.org/wp-content/uploads/2010/11/Water-Quality-Guidelines-for-AB-Final_0.pdf (Accessed September 18. 2014).
**Argentine** Republic	Argentine Official Gazette (1993)	Spanish	Argentine Official Gazette 1993. Regulatory Decree Law 24,051 on hazardous waste regime (In Spanish). http://www.ambiente.gov.ar/?aplicacion=normativa&IdNorma=538&IdSeccion=22 (Accessed August. 14. 2014).
Commonwealth of **Australia**	National Health and Medical Research Council (2013)	English	National Health and Medical Research Council, 2013. Australian Drinking Water Guidelines. http://www.nhmrc.gov.au/_files_nhmrc/publications/attachments/eh52_aust_drinking_water_guidelines_update_131216.pdf (Accessed August 15. 2014).
(Australia) **Australian Capital Territory**	Australian Capital Territory Ministry of Health (2007)	English	Australian Capital Territory Ministry of Health. Health (Drinking Water) Code of Practice. http://www.legislation.act.gov.au/di/2007-62/current/pdf/2007-62.pdf (Accessed August 15. 2014).
(Australia) State of **Tasmania**	Department of Health and Human Services (1997)	English	Department of Health and Human Services, 1997.Public Health Act 1997 Drinking Water Quality Guidelines. http://www.dhhs.tas.gov.au/__data/assets/pdf_file/0013/53320/drinking_water_quality_guidelines17_nov_05.pdf (Accessed August 15. 2014).
(Australia) State of **New South Wales**	Department of Health (2014)	English	Department of Health, 2014. Drinking Water Quality. http://www.health.nsw.gov.au/environment/water/Pages/drinking-water.aspx (Accessed August 15. 2014).
(Australia) State of **Northern Australia**	Department of Health (2014)	English	Department of Health, 2014. Water Quality. http://www.health.nt.gov.au/environmental_health/water_quality/index.aspx (Accessed August 15. 2014).
(Australia) State of **Queensland**	Government of Queensland (2014)	English	Government of Queensland, 2014. Public Health Regulation. https://www.legislation.qld.gov.au/LEGISLTN/CURRENT/P/PubHealR05.pdf (Accessed August 15. 2014).
(Australia) State of **South Australia**	Government of South Australia (2011)	English	Government of South Australia, 2011. Safe Drinking Water Act. http://www.sahealth.sa.gov.au/wps/wcm/connect/Public+Content/SA+Health+Internet/Protecting+public+health/Water+quality/ (Accessed August 15. 2014).

(Australia) State of **Western Australia**	Department of Health (2014)	English	Department of Health, 2014. Australian drinking water guidelines. http://www.public.health.wa.gov.au/3/657/2/australian_drinking_water_guidelines.pm (Accessed August 15. 2014).
Republic of **Austria**	Austria Department of Health (2013)	German	Austria Department of Health, 2013. Total legislation for drinking water regulations, as amended on 22.02.2013 (In German). https://www.verbrauchergesundheit.gv.at/lebensmittel/rechtsvorschriften/oesterreich/mwasser.html (Accessed August 16. 2014).
Commonwealth of the **Bahamas**	The Bahamas Water and Sewerage Corporation (1999)	English	The Bahamas Water and Sewerage Corporation, 1999. Customer Service Guide. http://www.wsc.com.bs/WSC_Customer%20Service%20Guide_Nov2012.pdf (Accessed August 16. 2014).
People's Republic of **Bangladesh**	Amio Water Treatment Limited (2010)	English	Amio Water Treatment Limited, 2010. Bangladesh drinking water quality standards. http://www.amiowater.com/index.php?menu_id=53&exmenu=53&page=1 (Accessed September 10. 2014).
Republic of **Belarus**	Belarus Ministry of Health (2013)	Russian	Belarus Ministry of Health, 2013. On approval of the Sanitary rules and regulations" Hygienic requirements for drinking water, packaged in containers" (In Russian). http://pravo.newsby.org/belarus/postanov12/pst861.htm (Accessed August 16. 2014).
Kingdom of **Belgium**	Brussels-Capital Region Government (2014)	English	Brussels-Capital Region Government, 2014. Annual water consumption in the Brussels-Capital Region is around 60 million cubic meters. http://www.brussels.irisnet.be/living-in-brussels/environment/water-quality (Accessed August 18. 2014).
**Belize**	Belize Agricultural Health Authority (2003)	English	Belize Agricultural Health Authority, 2003. Belize Agricultural Health Authority Act. http://www.vertic.org/media/National%20Legislation/Belize/BZ_Agricultural_Health_Authority_Act.pdf (Accessed September 18. 2014).
Kingdom of **Bhutan**	Codex Alimentarius (2001)	English	Codex Alimentarius, 2001. General Standard for Bottled/Packaged Drinking Waters. www.codexalimentarius.org/input/download/standards/.../CXP_048e.pdf (Accessed September 17. 2014).
Plurinational State of **Bolivia**	Bolivia Ministry of Public Works and Services Vice of Basic Services (2004)	Spanish	Bolivia Ministry of Public Works and Services Vice of Basic Services, 2004. Potable Water – Requirements (In Spanish). http://paap.mmaya.gob.bo/__ucp/agua_saneamiento/NORMAS/NB%20512,495-496%20CalidadAgua%20-%20oct2004/NB512%20AP_Requisitos.pdf (Accessed August 18. 2014).
Republic of **Botswana**	Water Utilities Corporation (2000)	English	Water Utilities Corporation, 2000. Botswana Standard for Drinking Water Quality Specification (BOS 32:2000). http://www.limpoporak.com/_system/DMSStorage/3461en/Botswana_WQGuidelines__BOS_32_2000.pdf (Accessed September 10. 2014).
Federative Republic of **Brazil**	Brazil Ministry of Health (2004)	Portuguese	Brazil Ministry of Health, 2004. Ordinance MS No 518/2004 (In Portuguese). http://www.tratamentodeagua.com.br/a1/artigo/portaria_quinhentos_e_dezoito.pdf (Accessed August 19. 2014).
(Brazil)State of **San Paolo**	Government of State of San Paolo (2008)	Portuguese	Government of State of San Paolo, 2008. Guiding Values of Intervention State of São Paulo (in Portuguese). http://www.mma.gov.br/port/conama/processos/FE4582B1/GTAreasContApresCETESB.pdf (Accessed August 20. 2014).
Republic of **Bulgaria**	Bulgaria Ministry of Health (2001)	Bulgarian	Bulgaria Ministry of Health, 2001. Ordinance № 9 dated 16.03.2001 on the quality of water for drinking and domestic purposes (in Bulgarian). http://www.vikmontana.com/docs/NAREDBA_9_ot_16032001.pdf (Accessed August 20. 2014).
Kingdom of **Cambodia**	Cambodia Ministry of Industry Mines and Energy (2004)	English	Cambodia Ministry of Industry Mines and Energy, 2004. Drinking Water Quality Standards. http://www.rdic.org/MIME-Drinking-Water-Quality-Standards-2004-en.pdf (Accessed September 10. 2014).
**Canada**	Health Canada (2012)	English	Health Canada, 2012. Guidelines for Canadian Drinking Water Quality - Summary Table. http://www.hc-sc.gc.ca/ewh-semt/pubs/water-eau/2012-sum_guide-res_recom/index-eng.php (Accessed August 20. 2014).
(Canada) Province of **Alberta**	Alberta Health Services (2013)	English	Alberta Health Services, 2013. Water Quality Regulations (Alberta). http://www.albertahealthservices.ca/Advisories/ne-pha-water-quality-backgrounder.pdf (Accessed August 20. 2014).

(Canada) Province of **British Columbia**	British Columbia Ministry of Health (undated)	English	British Columbia Ministry of Health. Drinking Water Treatment Objectives (Microbiological) for Surface Water Supplies in British Columbia. http://www.health.gov.bc.ca/protect/dw_treatment-objectives.html (Accessed August 21. 2014).
(Canada) Province of **Newfoundland and Labrador**	Government of Newfoundland and Labrador (2013)	English	Government of Newfoundland and Labrador, 2013. Drinking Water Safety in Newfoundland and Labrador. http://www.env.gov.nl.ca/env/waterres/reports/drinking_water/annual_report_2013.pdf (Accessed August 20. 2014).
(Canada) **Northwest Territories**	Canada Northwest Territories Municipal and Community Affairs (undated)	English	Canada Northwest Territories Municipal and Community Affairs. Northwest Territories Drinking Water Quality Database. http://www.maca.gov.nt.ca/operations/water/homepage.asp (Accessed August 20. 2014).
Canada **Nova Scotia**	Government of Nova Scotia (2012)	English	Government of Nova Scotia, 2012. Nova Scotia Treatment Standards for Municipal Drinking Water Systems. http://www.novascotia.ca/nse/water/docs/Treatment_Standards_for_Municipal_Drinking_Water_Systems.pdf (Accessed August 21. 2014).
	Nova Scotia Environment and Labor (undated)	English	Nova Scotia Environment and Labor. A Guide for Municipal Water Works on How the Guidelines for Canadian Drinking Water Quality are Implemented in Nova Scotia . http://www.novascotia.ca/nse/water/docs/GuideonGuidelinesforCanadianDrinkingWaterQuality.pdf (Accessed August 21. 2014).
(Canada) **Nunavut**	National Collaborating Centre for Environmental Health (2014)	English	National Collaborating Centre for Environmental Health, 2014. Small Drinking Water Systems: Who Does What in Nunavut. http://ncceh.ca/sites/default/files/SDWS_Who_What_Nunavut.pdf (Accessed August 21. 2014).
(Canada) Province of **Ontario**	Canadian Institute for Environmental Law and Policy (2003)	English	Canadian Institute for Environmental Law and Policy, 2003. Drinking Water Quality Standards in Ontario – Are They Tough. http://cielap.org/pdf/drinkingwaterstandards.pdf (Accessed August 21. 2014).
(Canada) Province of **Prince Edward Island**	Prince Edward Island Department of Environment, Labor and Justice (2012)	English	Prince Edward Island Department of Environment, Labor and Justice, 2012. Private Water Supplies (Wells). http://www.gov.pe.ca/environment/index.php3?number=1015748&lang=E (Accessed August 21. 2014).
(Canada) Province of **Quebec**	Government of Quebec (2014)	English	Government of Quebec, 2014. Regulation respecting the quality of drinking water. http://www2.publicationsduquebec.gouv.qc.ca/dynamicSearch/telecharge.php?type=2&file=//Q_2/Q2R40_A.htm (Accessed August 21. 2014).
(Canada) Province of **Saskatchewan**	Saskatchewan Environment (2006)	English	Saskatchewan Environment, 2006. Saskatchewan’s Drinking Water Quality Standards and Objectives (summarized). http://www.saskh20.ca/DWBinder/EPB207Drinking_Water_Standards_post.pdf (Accessed August 21. 2014).
(Canada) **Yukon**	Government of Yukon (2007)	English and French	Government of Yukon, 2007. Public Health and Safety Act (In English and French). http://www.gov.yk.ca/legislation/regs/oic2007_139.pdf (Accessed August 21. 2014).
Republic of **Chile**	Chile Ministry of Public Works (2005)	Spanish	Chile Ministry of Public Works, 2005. NCH 409/1 Drinking Water Quality Standard (In Spanish). http://ciperchile.cl/pdfs/11-2013/norovirus/NCh409.pdf (Accessed August 22. 2014).
People’s Republic of **China**	China Department of Health (2007)	Chinese	China Department of Health, 2007. GB 5749-85 Standards for Drinking Water Quality 2007 (In Chinese). http://www.sx-watersupply.gov.cn/NewsDetails.asp?d=1557 (Accessed August 22. 2014).
Republic of **Colombia**	Colombian Institute for Technical Standards and Certification (1994)	Spanish	Colombian Institute for Technical Standards and Certification, 1994. Official standards for water quality Colombia (In Spanish) https://www.imta.gob.mx/cotennser/images/docs/NOI/Normas_oficiales_para_la_calidad_del_agua_colombia.pdf (Accessed August 22. 2014).
Republic of **Costa Rica**	Costa Rica Minister of Finance (2005)	Spanish	Costa Rica Minister of Finance, 2005. Drinking water regulation (in Spanish). http://www.aguanalisis.com/downloads/Reglamento-de-Calidad-del-Agua-Potable-32327-S-La-Gaceta-84.pdf (Accessed August 23. 2014).

Republic of **Croatia**	Croatia Ministry of Health and Social Welfare (2007)	Croatian	Croatia Ministry of Health and Social Welfare, 2007. Ordinance Health Drinking Water (in Croatian). http://narodne-novine.nn.hr/clanci/sluzbeni/339082.html (Accessed August 19. 2014).
Republic of **Cuba**	Cuba Government (1997)	Spanish	Cuba Government, 1997. Communal Hygiene Drinking Water Health Requirements (In Spanish). https://www.imta.gob.mx/cotennser/images/docs/NOI/Normas_oficiales_para_la_calidad_del_agua_cuba.pdf (Accessed September 10. 2014).
Republic of **Cyprus**	Cyprus Ministry of Agriculture, Natural Resources and Environment (1999)	English	Cyprus Ministry of Agriculture, Natural Resources, and Environment, 1999. Water Treatment And Desalination Plants. http://www.moa.gov.cy/moa/wdd/wdd.nsf/booklets_en/DAEF71F997D25DF9C2256E85004B1583/$file/pages%201-23%20(2.41MB).pdf (Accessed September 15. 2014).
**Czech** Republic	European Commission (1998)	Czech	European Commission, 1998. The quality of water intended for human consumption (In Czech). http://www.sgpstandard.cz/editor/files/on_line/styk/demo/voda/eu/1998_83_es.pdf (Accessed August 25. 2014).
Kingdom of **Denmark**	Nature Agency of Denmark (2014)	Danish	Nature Agency of Denmark, 2014. Ordinance 292 of 26/03/2014 (In Danish). http://naturstyrelsen.dk/vandmiljoe/vand-i-hverdagen/drikkevand/ (Accessed August 25. 2014).
**Dominican** Republic	Dominican Ministry of Public Health and Social Assistance (2005)	Spanish	Dominican Ministry of Public Health and Social Assistance, 2005. DECREE 42-05 Make the Rules of Drinking Water (In Spanish). http://salud.gob.do/download/docs/Decretos/Decreto42-05.pdf (Accessed September 10. 2014).
Republic of **Ecuador**	Ecuadorian Institute of Standards (2011)	Spanish	Ecuadorian Institute of Standards, 2011. Technical Standard Ecuador NTE INEN 1108: 2011 fourth review (In Spanish).https://law.resource.org/pub/ec/ibr/ec.nte.1108.2011.pdf (Accessed August 25. 2014).
Arab Republic of **Egypt**	WHO-Regional Office for the Eastern Mediterranean (2006)	English	WHO-Regional Office for the Eastern Mediterranean, 2006. A compendium of drinking-water quality standards in the Eastern Mediterranean Region. http://applications.emro.who.int/dsaf/dsa1183.pdf (Accessed September 14. 2014).
Republic of **Estonia**	Estonia Minister of Social Affairs (2013)	Estonian	Estonia Minister of Social Affairs, 2013. Drinking water quality and control requirements and analysis methods 2013 (In Estonian). https://www.riigiteataja.ee/akt/111012013002 (Accessed August 26. 2014).
Federal Democratic Republic of **Ethiopia**	World Health Organization (2010)	English	World Health Organization, 2010. Rapid Assessment of Drinking-Water Quality in the Federal Democratic Republic of Ethiopia. http://www.wssinfo.org/fileadmin/user_upload/resources/RADWQ_Ethiopia.pdf (Accessed September 16. 2014).
Republic of **Fiji**	Secretariat of the Pacific Community (2005)	English	Secretariat of the Pacific Community, 2005. Drinking Water Quality in the Pacific Island Countries: Situation Analysis and Needs Assessment. http://ict.sopac.org/VirLib/JC0181.pdf (Accessed September 22. 2014).
Republic of **Finland**	Finland Minister of Social Affairs and Health (2001)	Finish	Finland Minister of Social Affairs and Health, 2001. Social Affairs and Health Decree of the Ministry of drinking water quality standards, and surveillance studies 461/2000 (In Finish). http://www.finlex.fi/fi/laki/alkup/2001/20010401 (Accessed August 26. 2014).
**French** Republic	France Ministry of Ecology, Sustainable Development and Energy (1998)	French	France Ministry of Ecology, Sustainable Development, and Energy, 1998. DIRECTIVE 98/83 / EC 1998 (In French). http://eur-lex.europa.eu/LexUriServ/LexUriServ.do?uri=OJ:L:1998:330:0032:0054:FR:PDF (Accessed August 26. 2014).
Republic of the **Gambia**	Gambia Environmental Quality Standards Board (1999)	English	Gambia Environmental Quality Standards Board, 1999. Environmental Quality Standards Regulations. http://faolex.fao.org/docs/pdf/gam95812.pdf (Accessed September 16. 2014).
**Georgia**	Georgia Ministry of Justice (2007)	Georgian	Georgia Ministry of Justice, 2007. Technical regulations for drinking water (In Georgian). http://www.momxmarebeli.ge/images/file_955911.pdf (Accessed August 26. 2014).
Federal Republic of **Germany**	Germany Federal Ministry of Justice and Consumer Protection (2001)	German	Germany Federal Ministry of Justice and Consumer Protection, 2001. Regulation on the quality of water intended for human consumption 2001 (In German). http://www.gesetze-im-internet.de/trinkwv_2001/index.html#BJNR095910001BJNE000603377 (Accessed August 26. 2014).

**Hellenic** Republic	Greece CENTRAL PUBLIC HEALTH LABORATORY (1998)	Greek	Greece Central Public Health Laboratory, 1998. Directive 98/83 RC Council Of Europe Of 3-11-1998 "On the Water Quality of Human Consumption" (In Greek). http://www.edye.gr/htm2/EFHMER_NEA_ODHGIA.htm (Accessed August 26. 2014).
Republic of **Guatemala**	Guatemala Government (1999)	Spanish	Guatemala Government, 1999. Potable Water Standard (In Spanish). http://www.ecosistemas.com.gt/docs/01-COGUANOR-NGO-29001-1a-Revision-Agua-potable.pdf (Accessed September 19. 2014).
Republic of **Honduras**	Honduras Department of health (1995)	Spanish	Honduras Department of health, 1995. Technical standard for drinking water quality (In Spanish). http://www.salud.gob.hn/transparencia/archivos/regulacion/leyes/NORMA%20TECNICA%20CALIDAD%20AGUA%20POTABLE%20%20Honduras.pdf (Accessed September 19. 2014).
Republic of **Hungary**	Hungary National Public Health and Medical Officer Service (2001)	Hungarian	Hungary National Public Health and Medical Officer Service, 2001. Regulation (October 25th) on. Drinking water quality requirements and control arrangements (In Hungarian). http://net.jogtar.hu/jr/gen/hjegy_doc.cgi?docid=A0100201.KOR (Accessed August 26. 2014).
Republic of **Indonesia**	Indonesia Government (1990)	Indonesian	Indonesia Government, 1990. Water Quality Standards in Public Waters (In Indonesian). http://muhalikhsan.blogspot.com/2013/09/standar-kualitas-air-minum-negara.html (Accessed September 18. 2014).
Republic of **Iraq**	Iraq Central Agency for Meteorology and Quality Control (2001)	Arabic and English	Iraq Central Agency for Meteorology and Quality Control, 2001. Iraqi Legislation No. (417)-2001 Standard Specification for Drinking water (In Arabic and English). http://www.oil.gov.iq/Envirnment/PDF%20files/18.pdf (Accessed September 14. 2014).
**Ireland**	Ireland EPA (2007)	English	Ireland EPA, 2007. Standards for drinking water quality. https://www.epa.ie/pubs/advice/drinkingwater/privatewatersupplieshandbook/Section%202.pdf (Accessed August 27. 2014).
State of **Israel**	Israel Ministry of Health (2000)	Hebrew	Israel Ministry of Health, 2000. Public Health Regulations (Sanitary Quality of Drinking Water) (In Hebrew). www.old.health.gov.il/.../a205_israeli_drinking_water_regulations.doc (Accessed August 27. 2014).
Republic of **Italy**	Navy Medicine (2012)	English	Navy Medicine, 2012. Environmental Final Governing Standards Italy. http://www.med.navy.mil/sites/nmcphc/Documents/environmental-programs/bumed-eqp/overseas-environmental-compliance/FGS0-Italy-2012.pdf (Accessed August 27. 2014).
	Italy Ministry of Health (2001)	Italian	Italy Ministry of Health, 2001. General Directorate of Prevention (In Italian). http://www.arpa.veneto.it/temi-ambientali/acqua/file-e-allegati/normativa-2010/Decreto_legislativo_31_2001.pdf (Accessed August 27. 2014).
**Japan**	Japan Ministry of Health, Labor and Welfare (2001)	English and Japanese	Japan Ministry of Health, Labor and Welfare, 2001. General Standard for Bottled/Packaged Drinking Waters (In English and Japanese). http://www.mhlw.go.jp/stf/shingi/2r9852000000ip55-att/2r9852000000ipsg.pdf (Accessed August 27. 2014).
Hashemite Kingdom of **Jordan**	The Jordanian Institute of Standards and Metrology (2001)	English	The Jordanian Institute of Standards and Metrology, 2001. Drinking-water Standards: JS 286:2001. https://www3.opic.gov/environment/eia/ammanwater/Annex%20C21-Drinking%20Water%20Standards.pdf (Accessed September 14. 2014).
Republic of **Kazakhstan**	Kazakhstan Government (2001)	Russian	Kazakhstan Government, 2001. SanPin 2.1.4.1074-01 Drinking Water (In Russian). http://www.aquapool.kz/html/?id=116 (Accessed August 28. 2014).
Republic of **Kiribati**	Secretariat of the Pacific Community (2005)	English	Secretariat of the Pacific Community, 2005. Drinking Water Quality in the Pacific Island Countries: Situation Analysis and Needs Assessment. http://ict.sopac.org/VirLib/JC0181.pdf (Accessed September 22. 2014).
Republic of **Korea**	Korea Ministry of Environment (2011)	English	Korea Ministry of Environment, 2011. The Drinking Water Quality Standard. http://www.apec-vc.or.kr/?p_name=database&sort=program&sort2=EP5&gotopage=28&query=view&unique_num=ED2011081273 (Accessed September 4. 2014).
State of **Kuwait**	WHO-Regional Office for the Eastern Mediterranean (2006)	English	WHO-Regional Office for the Eastern Mediterranean, 2006. A compendium of drinking-water quality standards in the Eastern Mediterranean Region. http://applications.emro.who.int/dsaf/dsa1183.pdf (Accessed September 14. 2014).

Republic of **Latvia**	Latvia Ministry of Health (2004)	Latvian	Latvia Ministry of Health, 2004. Drinking water monitoring and control (In Latvian). http://www.vi.gov.lv/lv/vides-veseliba/dzeramais-udens/dzerama-udens-uzraudziba-un-kontrole (Accessed August 30. 2014).
**Lebanese** Republic	WHO-Regional Office for the Eastern Mediterranean (2006)	English	WHO-Regional Office for the Eastern Mediterranean, 2006. A compendium of drinking-water quality standards in the Eastern Mediterranean Region. http://applications.emro.who.int/dsaf/dsa1183.pdf (Accessed September 14. 2014).
Principality of **Liechtenstein**	Liechtenstein Drinking Water Inspectorate (1999)	English	Liechtenstein Drinking Water Inspectorate, 1999. Review on the Approaches to Water Quality Monitoring and Assessment in Europe. http://dwi.defra.gov.uk/research/completed-research/reports/dwi0799.pdf (Accessed September 16. 2014).
Republic of **Lithuania**	Lithuania Ministry of Health (2003)	Lithuanian	Lithuania Ministry of Health, 2003. Lithuanian Hygiene Standard HN 24: 2003 Drinking Water Safety and Quality Requirements (In Lithuanian). http://www.am.lt/VI/article.php3?article_id=8252 (Accessed August 31. 2014).
Grand Duchy of **Luxembourg**	Luxembourg Collection of Legislation (2002)	French	Luxembourg Collection of Legislation, 2002. Water Quality for Human Consumption (In French). http://www.legilux.public.lu/leg/a/archives/2002/0115/a115.pdf (Accessed August 31. 2014).
**Malaysia**	Malaysia Ministry of Health (2010)	English	Malaysia Ministry of Health, 2010. Drinking Water Quality Standard. http://kmam.moh.gov.my/public-user/drinking-water-quality-standard.html (Accessed August 31. 2014).
Republic of **Malta**	Malta government (2009)	Maltese	Malta government, 2009. Regulations 2009 Water Intended for Consumption Human (In Maltese). http://faolex.fao.org/docs/pdf/mlt84621.pdf (Accessed August 31. 2014).
Republic of **Mauritius**	Mauritius Government Gazette (1996)	English	Mauritius Government Gazette, 1996. Drinking Water Standards. http://environment.gov.mu/English/Pages/Legislations/Standards/Drinking-water.aspx (Accessed September 16. 2014).
United **Mexican** States	Government of Mexico (1994)	Spanish	Government of Mexico, 1994. Environmental Health Water Use for Human Consumption - Permissible Limits of Quality and Treatments to be Bound Water for Drinking Water (In Spanish). https://www.gov.mt/mt/Services-And-Information/Pages/Ir-Regolamentazzjoni-Ambjentali.aspx (Accessed August 31. 2014).
**Mongolia**	Government of Mongolia (2005)	Mongolian	Government of Mongolia, 2005. Drinking water Health and safety requirements, quality and safety assessment (In Mongolian). http://estandard.mn/filebase/files/0900-2005.pdf (Accessed September 1. 2014).
Kingdom of **Morocco**	WHO-Regional Office for the Eastern Mediterranean (2006)	English	WHO-Regional Office for the Eastern Mediterranean, 2006. A compendium of drinking-water quality standards in the Eastern Mediterranean Region. http://applications.emro.who.int/dsaf/dsa1183.pdf (Accessed September 14. 2014).
Republic of **Nauru**	Secretariat of the Pacific Community (2005)	English	Secretariat of the Pacific Community, 2005. Drinking Water Quality in the Pacific Island Countries: Situation Analysis and Needs Assessment. http://ict.sopac.org/VirLib/JC0181.pdf (Accessed September 22. 2014).
Kingdom of the **Netherlands**	Government of Netherlands (2014)	Dutch	Government of Netherlands, 2014. Drinking Water Decision (In Dutch). http://wetten.overheid.nl/BWBR0030111/geldigheidsdatum_02-09-2014 (Accessed September 2. 2014).
**New Zealand**	New Zealand Ministry of Health (2008)	English	New Zealand Ministry of Health, 2008. Drinking-water Standards for New Zealand. http://www.moh.govt.nz/notebook/nbbooks.nsf/0/237C397A37EFADA9CC25750600720E08/$file/drinking-water-standards-2008.pdf (Accessed September 2. 2014).
Republic of **Nicaragua**	Nicaragua Ministry of Health (1994)	Spanish	Nicaragua Ministry of Health, 1994. Water Quality Standards for Consumer Human (In Spanish). https://www.imta.gob.mx/cotennser/images/docs/NOI/Normas_oficiales_para_la_calidad_del_agua_nicaragua.pdf (Accessed September 19. 2014).
Federal Republic of **Nigeria**	Standards Organization of Nigeria (2007)	English	Standards Organization of Nigeria, 2007. Nigerian Standard for Drinking Water Quality. http://www.unicef.org/nigeria/ng_publications_Nigerian_Standard_for_Drinking_Water_Quality.pdf (Accessed September 2. 2014).
Kingdom of **Norway**	Norway Ministry of Health and Care Services (2001)	Norwegian	Norway Ministry of Health and Care Services, 2001. Regulations on water supply and drinking water (Drinking Water Regulations) (In Norwegian). http://lovdata.no/dokument/SF/forskrift/2001-12-04-1372 (Accessed September 2. 2014).

Islamic Republic of **Pakistan**	Pakistan Standards and Quality Control Authority (undated)	English	Pakistan Standards and Quality Control Authority. Drinking Water Quality Guidelines and Standards. http://www.pcrwr.gov.pk/Press%20Releases%202012/COMSTECH%20Workshop/Ms%20Saiqa%20Imran/05-Water%20Quality%20Standards.pdf (Accessed September 11. 2014).
Republic of **Palau**	Environmental Quality Protection Board (undated)	English	Environmental Quality Protection Board. Public Water Supply System Regulations. http://faolex.fao.org/docs/pdf/pau32797.pdf (Accessed September 22. 2014).
Republic of **Peru**	Peru Ministry of Health (2011)	Spanish	Peru Ministry of Health, 2011. Regulation of Water Quality Human Consumption (In Spanish). http://www.digesa.minsa.gob.pe/publicaciones/descargas/reglamento_calidad_agua.pdf (Accessed September 3. 2014).
Republic of the **Philippines**	Philippines Department of Health (2007)	English	Philippines Department of Health, 2007. Philippine National Standards for Drinking Water. http://www.lwua.gov.ph/downloads_14/Philippine%20National%20Standards%20for%20Drinking%20Water%202007.pdf (Accessed September 18. 2014).
Republic of **Poland**	Poland Ministry of Health (2007)	Polish	Poland Ministry of Health, 2007. The quality of water intended for human use (In Polish). http://www.wodociagiostrzeszowskie.pl/asp/pliki/pobierz/rozporzadzenie-_jakosc_wody.pdf (Accessed September 3. 2014).
**Portuguese** Republic	Portugal Ministry of Environment, Planning and Regional Development (2007)	Portuguese	Portugal Ministry of Environment, Planning and Regional Development, 2007. Gazette, 1st Series - No. 164 - August 27, 2007 (In Portuguese). http://dre.pt/pdf1s/2007/08/16400/0574705765.pdf (Accessed September 3. 2014).
State of **Qatar**	The Gulf Cooperation Council (GCC) Standardization (2012)	Arabic and English	The Gulf Cooperation Council (GCC) Standardization, 2012. Bottled drinking water (In Arabic and English). http://old.sfda.gov.sa/NR/rdonlyres/144C70B0-4648-4A6E-A06C-99EEB6FB8772/0/%D9%85%D9%8A%D8%A7%D9%87%D8%A7%D9%84%D8%B4%D8%B1%D8%A8%D8%A7%D9%84%D9%85%D8%B9%D8%A8%D8%A7%D8%A9%D8%AE%D9%84%D9%8A%D8%AC%D9%8A%D8%A7%D9%86%D8%AC%D9%84%D9%8A%D8%B2%D9%8A1.pdf (Accessed September 3. 2014).
**Russian** Federation	Russian Ministry of Health (2002)	Russian	Russian Ministry of Health, 2002. DRINKING WATER.HYGIENE REQUIREMENTS FOR QUALITY WATER SanPin 2.1.4.1074-01 (In Russian). http://ross-water.com/files/sanpin/file-2.pdf?1242648352 (Accessed August 28. 2014).
	Russian Ministry of Health (2002)	Russian	Russian Ministry of Health, 2002. Drinking water. Hygienic requirements for water quality, packaged in a container Quality control. SanPin 2.1.4.1116-02 (In Russian). http://www.lin.irk.ru/new/files/2141116-02.pdf (Accessed August 28. 2014).
	Russian Ministry of Health (2007)	Russian	Russian Ministry of Health, 2007. Maximum permissible concentration (MPC) CHEMICALS in Water drinking and cultural and COMMUNITY WATER (In Russian). http://www.ohranatruda.ru/ot_biblio/normativ/data_normativ/41/41363/index.php (Accessed August 28. 2014).
	Russian Ministry of Health (1999)	Russian	Russian Ministry of Health, 1999. Maximum permissible concentration (MPC) of chemicals in water bodies drinking and cultural and community water Hygienic standards GN 2.1.5.2280-07 (In Russian). http://www.ohranatruda.ru/ot_biblio/normativ/data_normativ/51/51955/index.php (Accessed August 28. 2014).
	Russian Ministry of Health (1998)	Russian	Russian Ministry of Health, 1998. Drinking Water. Hygienic requirements for quality of centralized drinking water supply. Quality Control SanPin 2.1.4.559-96 (In Russian). http://www.opengost.ru/iso/13_gosty_iso/13060_gost_iso/1306020_gost_iso/3989-mu-2.1.4.682-97-metodicheskie-ukazaniya-po-vnedreniyu-i-primeneniyu-sanitarnyh-pravil-i-norm-sanpin-2.1.4.559-96-pitevaya-voda.html (Accessed August 28. 2014).
Republic of **Rwanda**	Rwanda Standards Board (2013)	English	Rwanda Standards Board, 2013. Drinking Water Standards to be Finalized In EAC Region. http://www.rwanda-standards.org/~rbs/main-nav/media-room/news-and-events/news-details/article/drinking-water-standards-to-be-finalised-in-eac-region-supfont-colorrednewfontsup.html (Accessed September 17. 2014).
**Saint Lucia**	Caricom Regional Organization for Standards and Quality (undated)	English	Caricom Regional Organization for Standards and Quality. Guidelines for Recreational Water Quality. http://www.ssb.sr/smartcms/downloads/dcrs%2036%20guidelines%20for%20recreational%20water%20qualityDRAFT.pdf (Accessed September 21. 2014).

Republic of **Serbia**	Serbia Official Gazette (1999)	English	Serbia Official Gazette, 1999. The hygienic quality of drinking water. www.zjz.org.rs/wp-content/uploads/2013/04/pravilnik-o-higijenskoj-ispravnosti-vode-za-pice.pdf+&cd=1&hl=en&ct=clnk&gl=us (Accessed August 19. 2014).
Republic of **Singapore**	Government of Singapore (2008)	English	Government of Singapore, 2008. Environmental Public Health (Quality of Piped Drinking Water) Regulations. http://www.wsportal.org/uploads/IWA%20Toolboxes/WSP/EPH%20Regulations.pdf (Accessed September 5. 2014).
**Slovak** Republic	Council Regulation Government of the Slovak Republic (2010)	Slovak	Council Regulation Government of the Slovak Republic, 2010. Laying down requirements for water intended for human consumption and control of the quality of water intended for human consumption (In Slovak). http://www.ecoli.sk/files/documents/nv_496_2010.pdf (Accessed September 5. 2014).
Republic of **Slovenia**	Slovenia Ministry of Health (2004)	Slovenian	Slovenia Ministry of Health, 2004. RULES of drinking water (In Slovenian). http://www.uradni-list.si/files/RS_-2004-019-00865-OB~P001-0000.PDF#!/pdf (Accessed September 5. 2014).
Republic of **South Africa**	South Africa Department of water and sanitation (2005)	English	South Africa Department of water and sanitation, 2005. Drinking Water Quality Management Guide for Water Services Authorities. http://www.dwaf.gov.za/Documents/Other/DWQM/DWQMWSAGuideSept05.pdf (Accessed September 7. 2014).
Kingdom of **Spain**	Government of Spain (2003)	Spanish	Government of Spain, 2003. Quality drinking water by the health criteria are established (In Spanish). http://www.aguaplaza.com/download/agua_potable_consumo_humano_rd_140_2003.pdf (Accessed September 7. 2014).
Republic of the **Sudan**	WHO-Regional Office for the Eastern Mediterranean (2006)	English	WHO-Regional Office for the Eastern Mediterranean, 2006. A compendium of drinking-water quality standards in the Eastern Mediterranean Region. http://applications.emro.who.int/dsaf/dsa1183.pdf (Accessed September 14. 2014).
Kingdom of **Sweden**	Sweden Nutrition and Food Agency, 2001	Swedish	Sweden Nutrition and Food Agency, 2001. NFA regulations on drinking water (In Swedish). http://www.svensktvatten.se/Documents/Kategorier/Dricksvatten/Lagar%20och%20f%C3%B6reskrifter/F%C3%B6reskrifter%20dricksvatten%202011_3.pdf (Accessed September 7. 2014).
**Swiss** Confederation	Switzerland Department of Consumer and Veterinary (2014)	French	Switzerland Department of Consumer and Veterinary, 2014. Chemical standards for drinking water Federal Ordinance on foreign substances and components (In French). http://www.vd.ch/fileadmin/user_upload/organisation/dse/scav/sire/Eaux_potables_Normes_Composition_chimique.pdf (Accessed September 7. 2014).
**Syrian** Arab Republic	WHO-Regional Office for the Eastern Mediterranean (2006)	English	WHO-Regional Office for the Eastern Mediterranean, 2006. A compendium of drinking-water quality standards in the Eastern Mediterranean Region. http://applications.emro.who.int/dsaf/dsa1183.pdf (Accessed September 14. 2014).
United Republic of **Tanzania**	Tanzania Bureau of Standards (2009)	English	Tanzania Bureau of Standards, 2009. Tanzania Water Quality Standards. http://www.frameweb.org/CommunityBrowser.aspx?id=4408 (Accessed September 7. 2014).
Kingdom of **Thailand**	Thailand Ministry of Health (2001)	Thai	Thailand Ministry of Health, 2001. (No. 256) BE 2545 Drinking Water in sealed containers (No. 4) (In Thai). http://plan.dgr.go.th/school/5.pdf (Accessed September 7. 2014).
Kingdom of **Tonga**	Secretariat of the Pacific Community (2005)	English	Secretariat of the Pacific Community, 2005. Drinking Water Quality in the Pacific Island Countries: Situation Analysis and Needs Assessment. http://ict.sopac.org/VirLib/JC0181.pdf (Accessed September 22. 2014).
Republic of **Tunisia**	Global water and wastewater quality regulations (2012)	English	Global water and wastewater quality regulations, 2012. 57.1 Regulatory framework for drinking water, wastewater discharge and reuse. http://www.globalwaterintel.com/client_media/uploaded/Global%20Regulation/Sample_Chapter.pdf (Accessed September 12. 2014).
**Tuvalu**	Secretariat of the Pacific Community (2005)	English	Secretariat of the Pacific Community, 2005. Drinking Water Quality in the Pacific Island Countries: Situation Analysis and Needs Assessment. http://ict.sopac.org/VirLib/JC0181.pdf (Accessed September 22. 2014).
Republic of **Uganda**	Uganda Ministry of Tourism, Trade and Industry (2008)	English	Uganda Ministry of Tourism, Trade and Industry, 2008. Drinking (potable) Water Specification. https://law.resource.org/pub/ug/ibr/us.201.2008.html (Accessed September 17. 2014).

**Ukraine**	Ukraine Water Health (undated)	Russian	Ukraine Water Health. Drinking water quality standards in Ukraine (In Russian). http://water-health.org.ua/?Standarty_kachestva_pitmzevoi_vody:Normativy_kachestva_pitmzevoi_vody_v_Ukraine (Accessed September 8. 2014).
**United Kingdom** of Great Britain and Northern Ireland	United Kingdom Drinking Water Inspectorate (2000)	English	United Kingdom Drinking Water Inspectorate, 2000. Guidance on the Implementation of the Water Supply (Water Quality) Regulations 2000 (as amended) IN ENGLAND. http://dwi.defra.gov.uk/stakeholders/guidance-and-codes-of-practice/WS(WQ)-regs-england2010.pdf (Accessed September 8. 2014).
**Northern Ireland**	Statutory Rules of Northern Ireland (2007)	English	STATUTORY RULES OF NORTHERN IRELAND, 2007. The Water Supply (Water Quality) Regulations (Northern Ireland). http://www.legislation.gov.uk/nisr/2007/147/pdfs/nisr_20070147_en.pdf (Accessed September 2. 2014).
Eastern Republic of **Uruguay**	Uruguay Administration of Sanitary Works (2006)	Spanish	Uruguay Administration of Sanitary Works, 2006. Internal Quality Standard Drinking Water (In Spanish). http://www.ose.com.uy/descargas/reclutamiento/norma_interna_de_calidad_agua_potable_de_OSE.pdf (Accessed September 8. 2014).
Republic of **Uzbekistan**	Uzbekistan Ministry of Health (2006)	Russian	Uzbekistan Ministry of Health, 2006. Health Criteria and Control Water Quality of Centralized Drinking Water Supply of the Uzbek Population (In Russian). http://www.lex.uz/pages/GetAct.aspx?lact_id=1934624 (Accessed September 9. 2014).
Republic of **Vanuatu**	Secretariat of the Pacific Community (2005)	English	Secretariat of the Pacific Community, 2005. Drinking Water Quality in the Pacific Island Countries: Situation Analysis and Needs Assessment. http://ict.sopac.org/VirLib/JC0181.pdf (Accessed September 22. 2014).
	Secretariat of the Pacific Community (2005)	English	
Bolivarian Republic of **Venezuela**	Venezuela Ministry of Health and Welfare (1998)	Spanish	Venezuela Ministry of Health And Welfare, 1998. Health Standards for Drinking Water Quality (In Spanish). http://www.bvsde.paho.org/bvsacg/e/cd-cagua/normas/lac/20.VEN/01.norma.pdf (Accessed September 9. 2014).
Socialist Republic of **Viet Nam**	Vietnam Ministry of Health (2002)	Vietnamese	Vietnam Ministry of Health, 2002. Water hygiene standards (In Vietnamese). http://thuvienphapluat.vn/archive/Quyet-dinh-1329-2002-Q%C3%90-BYT-Tieu-chuan-ve-sinh-nuoc-an-uong-vb15749.aspx (Accessed September 9. 2014).
Jurisdictions other than United Nations member states			
**Palestine**	Palestinian Water Authority (1997)	Arabic	Palestinian Water Authority, 1997. Measurements of the quality of drinking water in Palestine (In Arabic). site.iugaza.edu.ps/amughari/files/Palestinian-Drinking-water-Quality-stndrds.doc+&cd=1&hl=en&ct=clnk&gl=us (Accessed September 11. 2014).
**Union of Soviet Socialist Republics** (USSR)	Medical Officer of the USSR (1981)	Russian	Medical Officer of the USSR, 1981. Guidelines for Hygiene Control Products from Synthetic Materials Offered for Use in Practice Drinking-water supply (In Russian). http://www.libussr.ru/doc_ussr/usr_10683.htm (Accessed August 29. 2014).
	State Sanitary of the USSR (1987)	Russian	State Sanitary of the USSR, 1987. Instruction Sanitary and Chemical Research Products of Plastics, Used for Use in Domestic and Drinking Water Supply and Water Resources (In Russian). http://www.libussr.ru/doc_ussr/usr_13837.htm (Accessed August 29. 2014).

Table S3. U.S. pesticide soil regulatory jurisdictions references and dates accessed.

Jurisdictions	Sources	Website References
	U.S. national jurisdictions	
U.S. Environmental Protection Agency	U.S. Environmental Protection Agency (2013)	U.S. Environmental Protection Agency, 2013. Risk-Based Screening Table. http://www.epa.gov/reg3hwmd/risk/human/rb-concentration_table/Generic_Tables/index.htm (accessed 10.01.14).
National Oceanic and Atmospheric Administration	National Oceanic and Atmospheric Administration Office of Response and Restoration (2008)	National Oceanic and Atmospheric Administration Office of Response and Restoration, 2008. Screening Quick Reference Tables. http://www.gesamp.org/data/gesamp/files/file_element/4a2a322c8acb2c26cc0234685eac71fa/SQuiRTs.pdf (accessed 23.01.14).
National Oceanic and Atmospheric Administration	Boeing Company, National Aeronautics and Space Administration and Department of Energy (2010)	Boeing Company, National Aeronautics and Space Administration and Department of Energy, 2010. Expedited List of Rural Residential Risk-Based Screening Levels – Tables. http://www.dtsc-ssfl.com/default.asp?V_DOC_ID=910&subID=8&subID2=8 (accessed 22.01.14).
Department of Energy	Boeing Company, National Aeronautics and Space Administration and Department of Energy (2010)	Boeing Company, National Aeronautics and Space Administration and Department of Energy, 2010. Expedited List of Rural Residential Risk-Based Screening Levels – Tables. http://www.dtsc-ssfl.com/default.asp?V_DOC_ID=910&subID=8&subID2=8 (accessed 22.01.14).
U.S. Army	U.S. Army Center for Health Promotion and Preventive Medicine (2013)	U.S. Army Center for Health Promotion and Preventive Medicine, 2013. Technical Guide 230 Environmental Health Risk Assessment and Chemical Exposure Guidelines for Deployed Military Personnel. http://phc.amedd.army.mil/PHC%20Resource%20Library/TG230.pdf (accessed 23.01.14).
Agency of Toxic Substance and Disease Registry	Agency of Toxic Substance and Disease Registry (2008, 2009a,f, 2010c, 2013)	Agency of Toxic Substance and Disease Registry, 2008. Public Health Assessment for KEN FOSTER FARM (FORMER) SHERWOOD, OREGON. http://www.atsdr.cdc.gov/hac/pha/KenFosterFarm/KenFosterFarmFinalPHA040308.pdf (accessed 29.01.14).
		Agency of Toxic Substance and Disease Registry, 2009a. Public Health Assessment PFOHL Brothers Landfill Cheektowaga, Erie County, New York. http://www.atsdr.cdc.gov/HAC/pha/PHA.asp?docid=253&pg=0 (accessed 29.01.14).
		Agency of Toxic Substance and Disease Registry, 2010c. Pesticide Misapplication in a Private Residence Parsons, Labette County, KS 67357. http://www.atsdr.cdc.gov/HAC/pha/ParsonsMalathionStrikeTeam/ParsonsMalathionStrikeTeamHC1117.pdf (accessed 29.01.14).
		Agency of Toxic Substance and Disease Registry, 2013. Wright Chemical Corporation NPL Site Adjacent to Livingston Creek Riegelwood, Columbus County, North Carolina. http://epi.publichealth.nc.gov/oee/hace/docs/WrightChemical_ColumbusCo.pdf (accessed 29.01.14).
	U.S. state and regional jurisdictions	
State of Alabama	Alabama Department of Environmental Management (2008)	Alabama Department of Environmental Management, 2008. Alabama Risk-Based Corrective Action Guidance Manual. http://adem.alabama.gov/programs/land/landforms/ARBCAManual.pdf (accessed 09.01.14).
State of Alaska	Alaska Department of Environmental Conservation (2012)	Alaska Department of Environmental Conservation, 2012. State of Alaska Selected Oil and Other Hazardous Substances Pollution Control Statutes and Regulations. http://dec.alaska.gov/spar/docs/18%20AAC%2075%20Rev%20April%208,%202012.pdf (accessed 09.01.14).
State of Arizona	Arizona Administrative Code (2009)	Arizona Administrative Code, 2009. Title 18-Environmental Quality Chapter, 7-Department Of Environmental Quality Remedial Action. http://www.azsos.gov/public_services/title_18/18-07.htm (accessed 09.01.14).
	Arizona Department of Environmental Quality (2002)	Arizona Department of Environmental Quality, 2002. Arizona Department of Environmental Quality UST Program Release Reporting & Corrective Action Guidance. http://www.azdeq.gov/environ/waste/ust/lust/tg.html (accessed 09.01.14).
State of Arkansas	Arkansas Department of Environmental Quality (2008)	Arkansas Department of Environmental Quality, 2008. Arkansas Department of Environmental Quality Clandestine Laboratory Remediation Cleanup Standards. http://www.adeq.state.ar.us/hazwaste/branch_programs/pdfs/clandestine_lab_cleanup_standards.pdf (accessed 10.01.14).
	U.S. Environmental Protection Agency (2013)	U.S. Environmental Protection Agency, 2013. Risk-Based Screening Table. http://www.epa.gov/reg3hwmd/risk/human/rb-concentration_table/Generic_Tables/index.htm (accessed 10.01.14).

State of California	California Environmental Protection Agency Office of Environmental Health Hazard Assessment (2010)	California Environmental Protection Agency Office of Environmental Health Hazard Assessment, 2010. Soil-Screening Numbers - Updated Table. http://oehha.ca.gov/risk/chhsltable.html (accessed 10.01.14).
(California) City of Oakland	City of Oakland Public Works Agency (2000)	City of Oakland Public Works Agency, 2000. Oakland Urban Land Redevelopment Program: Guidance Document. http://www2.oaklandnet.com/Government/index.htm (accessed 22.01.14).
(California) San Francisco Bay Regional Water Quality Control Board	San Francisco Bay Regional Water Quality Control Board (2013)	San Francisco Bay Regional Water Quality Control Board, 2013. ESL - Environmental Screening Levels. http://www.waterboards.ca.gov/rwqcb2/water_issues/programs/esl.shtml (accessed 22.01.14).
State of Colorado	Colorado Department of Public Health and Environment (2011)	Colorado Department of Public Health and Environment, 2011. Colorado Soil Evaluation Values (CSEV) Table. http://www.colorado.gov/cs/Satellite?blobcol=urldata&blobheadername1=Content-Disposition&blobheadername2=Content-Type&blobheadervalue1=inline%3B+filename%3D%22Colorado+Soil+Evaluation+Values+%28CSEV%29+Table.pdf%22&blobheadervalue2=application%2Fpdf&blobkey=id&blobtable=MungoBlobs&blobwhere=1251813117546&ssbinary=true (accessed 10.01.14).
State of Connecticut	Department of Energy and Environmental Protection (2013)	Department of Energy and Environmental Protection, 2013. Regulation of Department of Energy and Environmental Protection Concerning Remediation Standard. http://www.ct.gov/deep/lib/deep/regulations/22a/22a-133k-1through3.pdf (accessed 10.01.14).
State of Delaware	Delaware Department of Natural Resources and Environmental Control (1999, 2013)	Delaware Department of Natural Resources and Environmental Control, 1999. Remediation Standards Guidance under the Delaware Hazardous Substance Cleanup Act. http://www.dnrec.state.de.us/DNREC2000/Divisions/AWM/sirb/DOCS/PDFS/Misc/RemStnd.pdf (accessed 13.01.14).
		Delaware Department of Natural Resources and Environmental Control, 2013. Screening Level Table. http://www.dnrec.delaware.gov/dwhs/sirb/Documents/Screening%20Level%20Table.pdf (accessed 13.01.14).
State of Florida	Florida Department of Environmental Protection (2005)	Florida Department of Environmental Protection, 2005. Table II - Soil Cleanup Target Levels. http://www.dep.state.fl.us/waste/quick_topics/rules/documents/62-777/62-777_TableII_SoilCTLs.pdf (accessed 11.01.14).
(Florida) Miami-Dade County	Code of Miami-Dade County (2008)	Code of Miami-Dade County, 2008. Table II - Soil Cleanup Target Levels, Ordinance Amending Portions of Section 24-44 (2). http://www.miamidade.gov/environment/library/memos/rbca-ordinance.pdf (accessed 22.01.14).
State of Georgia	Georgia Department of Natural Resources (1993)	Georgia Department of Natural Resources, 1993. Appendix I Regulated Substances and Soil Concentrations That Trigger Notification. http://rules.sos.state.ga.us/docs/391/3/19/Appendix%20I-IV.pdf (accessed 11.01.14).
State of Hawaii	Hawaii Department of Health (2011)	Hawaii Department of Health, 2011. Environmental Hazard Evaluation and Environmental Action Levels. http://eha-web.doh.hawaii.gov/eha-cma/Leaders/HEER/environmental-hazard-evaluation-and-environmental-action-levels (accessed 14.01.14).
State of Idaho	Idaho Department of Environmental Quality (2004)	Idaho Department of Environmental Quality, 2004. Risk Evaluation Manuals Idaho Risk Evaluation Manual Final 2004-Appendix 1. http://www.deq.idaho.gov/waste-mgmt-remediation/remediation-activities/risk-evaluation-manuals.aspx (accessed 14.01.14).
State of Illinois	Illinois Administrative Code (2010)	Illinois Administrative Code, 2010. PART 742 Tiered Approach to Corrective Action Objectives, Section 742.TABLE I Chemicals Whose Tier 1 Class I Groundwater Remediation Objective. http://www.ipcb.state.il.us/documents/dsweb/Get/Document-38408 (accessed 14.01.14).
	Illinois Environmental Protection Agency (2011)	Illinois Environmental Protection Agency, 2011. Chemicals not in TACO Tier I Tables. http://www.epa.state.il.us/land/taco/chemicals-not-in-taco-tier-1-tables.html (accessed 14.01.14).
State of Indiana	Indiana Department of Environmental Management (2013)	Indiana Department of Environmental Management, 2013. Remediation Closure Guide with Corrections-Appendix A: Screening Levels. http://www.in.gov/idem/files/risc_screening_table_2013_explanatory.pdf (accessed 14.01.14).
State of Iowa	Iowa Department of Natural Resources (2013)	Iowa Department of Natural Resources, 2013. Statewide Standards for Contaminants in Soil and Groundwater. https://programs.iowadnr.gov/riskcalc/pages/standards.aspx (accessed 16.01.14).
State of Kansas	Kansas Department of Health and Environment (2010)	Kansas Department of Health and Environment, 2010. Risk-Based Standards For Kansas Risk Manual – 5th Version. http://www.kdheks.gov/remedial/download/RSK_Manual_10.pdf (accessed 14.01.14).
Commonwealth of Kentucky	Kentucky Energy and Environmental Cabinet (2011)	Kentucky Energy and Environmental Cabinet, 2011. Cleanup Standards for Voluntary Cleanup. http://waste.ky.gov/SFB/Pages/Brownfields.aspx (accessed 14.01.14).
	U.S. Environmental Protection Agency (2013)	U.S. Environmental Protection Agency, 2013. Risk-Based Screening Table. http://www.epa.gov/reg3hwmd/risk/human/rb-concentration_table/Generic_Tables/index.htm (accessed 10.01.14).

State of Louisiana	Louisiana Department of Environmental Quality (2003)	Louisiana Department of Environmental Quality, 2003. Recap 2003, Table 1 e Screening Option Screening Standards for Soil and Groundwater. http://www.deq.state.la.us/portal/Portals/0/RemediationServices/Table1.pdf (accessed 14.01.14).
State of Maine	Maine Department of Environmental Protection (2011, 2013)	Maine Department of Environmental Protection, 2011. Guidance for Human Health Risk Assessments for Hazardous Substance Sites in Maine February. http://www.maine.gov/dep/spills/publications/guidance/rags/final_5-8-2013/1%20Risk%20Manual-Feb_2011-%20CC.pdf (accessed 14.01.14).
		Maine Department of Environmental Protection, 2013. Maine Remedial Action Guidelines (RAGs) for Sites Contaminated with Hazardous Substances. http://www.maine.gov/dep/spills/publications/guidance/rags/final_5-8-2013/2%20ME-RAGS_Final_5-8-2013%20Corrected%20Copy.pdf (accessed 14.01.14).
	U.S. Environmental Protection Agency (2013)	U.S. Environmental Protection Agency, 2013. Risk-Based Screening Table. http://www.epa.gov/reg3hwmd/risk/human/rb-concentration_table/Generic_Tables/index.htm (accessed 10.01.14).
State of Maryland	Maryland Department of the Environment (2008)	Maryland Department of the Environment, 2008. Cleanup Standards for Soil and Groundwater. http://www.mde.state.md.us/assets/document/Final%20Update%20No%202.1%20dated%205-20-08(1).pdf (accessed 14.01.14).
Commonwealth of Massachusetts	Massachusetts Department of Environmental Protection (2014)	Massachusetts Department of Environmental Protection, 2014. 310 CMR 40.0000: Massachusetts Contingency Plan. http://www.mass.gov/eea/agencies/massdep/cleanup/regulations/massachusetts-contingency-plan.html (accessed 16.01.14).
State of Michigan	Michigan Department of Environmental Quality (2012)	Michigan Department of Environmental Quality, 2012. Attachment 1 Table 2. Soil: Residential Part 201 Generic Cleanup Criteria and Screening Levels. http://www.michigan.gov/documents/deq/deq-rrd-OpMemo_1-Attachment1Table2SoilResidential_283553_7.pdf (14.01.14).
State of Minnesota	Minnesota Pollution Control Agency (2009)	Minnesota Pollution Control Agency, 2009. Table A3.1 Summary of Tier 1 Soil Reference Value (SRY) Information (2009 Version). http://www.pca.state.mn.us/index.php/view-document.html?gid=3152 (accessed 14.01.14).
State of Mississippi	Mississippi Department of Environmental Quality (2002)	Mississippi Department of Environmental Quality, 2002. Mississippi Department of Environmental Quality Risk Evaluation Procedures for Voluntary Cleanup and Redevelopment of Brownfield Sites. http://www.deq.state.ms.us/MDEQ.nsf/pdf/GARD_brownfieldrisk/$File/Proced.pdf?OpenElement (accessed 14.01.14).
State of Missouri	Missouri Department of Natural Resources (2010)	Missouri Department of Natural Resources, 2010. Default Target Levels and Tier 1 Risk-Based Target Levels-Appendix B. http://www.dnr.mo.gov/env/hwp/mrbca/docs/appb-6-06.pdf (accessed 15.01.14).
State of Montana	Montana Department of Environmental Quality (2012)	Montana Department of Environmental Quality, 2012. Voluntary Cleanup and Redevelopment Act Application Guide. http://deq.mt.gov/StateSuperfund/vcraguide.mcpx (accessed 15.01.14).
	U.S. Environmental Protection Agency (2013)	U.S. Environmental Protection Agency, 2013. Risk-Based Screening Table. http://www.epa.gov/reg3hwmd/risk/human/rb-concentration_table/Generic_Tables/index.htm (accessed 10.01.14).
State of Nebraska	Nebraska Department of Environmental Quality (2012)	Nebraska Department of Environmental Quality, 2012. Nebraska Voluntary Cleanup Program Guidance NDEQ VCP Remediation Goals Table 1 - Direct Contact Exposure Pathways. http://www.deq.state.ne.us/Publica.nsf/23e5e39594c064ee852564ae004fa010/d243c2b56e34ea8486256f2700698997/$FILE/VCP%20GD%20Sept%202012%20FINAL.pdf (accessed 15.01.14).
State of Nevada	Nevada Division of Environmental Protection (2009, 2013)	Nevada Division of Environmental Protection, 2009. NDEP Draft Guidelines for Discovery Events (Soil RCs). http://ndep.nv.gov/bca/cem/cover.htm (accessed 15.01.14).
		Nevada Division of Environmental Protection, 2013. Nevada Division of Environmental Protection Basic Comparison Levels. http://ndep.nv.gov/bmi/docs/bcl_calculations_august_2013.pdf (accessed 15.01.14).
State of New Hampshire	New Hampshire Code of Administrative Rules (2008)	New Hampshire Code of Administrative Rules, 2008. New Hampshire Code of Administrative Rules. http://des.nh.gov/organization/commissioner/legal/rules/documents/env-or600.pdf (accessed 16.01.14).
		New Hampshire Department of Environmental Services, 2013. Appendix E, NHDES Risk Characterization and Management Policy. http://des.nh.gov/organization/divisions/waste/hwrb/documents/rcmp.pdf (accessed 15.01.14).
State of New Jersey	New Jersey Department of Environmental Protection (1999, 2012)	New Jersey Department of Environmental Protection, 1999. Soil Cleanup Criteria. http://www.nj.gov/dep/srp/guidance/scc/ (accessed 16.01.14).
		New Jersey Department of Environmental Protection, 2012. Appendix 1 Table 1A, Remediation Standards. http://www.nj.gov/dep/rules/rules/njac7_26d.pdf (accessed 16.01.14).
New Mexico	New Mexico Environment Department (2012)	New Mexico Environment Department, 2012. Appendix A Table A-1: NMED Soil Screening Levels, Risk Assessment Guidance for Site Investigations and Remediation.

		http://www.nmenv.state.nm.us/HWB/documents/NMED_RA_Guidance_for_SI_and_Remediation_6-14-2012.pdf (accessed 15.01.14).
	U.S. Environmental Protection Agency (2013)	U.S. Environmental Protection Agency, 2013. Risk-Based Screening Table. http://www.epa.gov/reg3hwmd/risk/human/rb-concentration_table/Generic_Tables/index.htm (accessed 10.01.14).
State of New York State	New York State Department of Environmental Conservation (2006, 2010)	New York State Department of Environmental Conservation, 2006. 6 NYCRR PART 375 Environmental Remediation Programs Subparts 375-1 to 375- 4 & 375-6. http://www.dec.ny.gov/docs/remediation_hudson_pdf/part375.pdf (accessed 17.01.14).
		New York State Department of Environmental Conservation, 2010. CP-51/Soil Cleanup Guidance. http://www.dec.ny.gov/docs/remediation_hudson_pdf/cpsoil.pdf (accessed 17.01.14).
(New York) New York City	New York City Office of Environmental Remediation (2014)	New York City Office of Environmental Remediation, 2014. NYC Voluntary Cleanup Program. http://www.nyc.gov/html/oer/html/voluntary-cleanup-program/vcp.shtml (accessed 18.04.14).
(New York) Suffolk County	Suffolk County Department of Health Services (2011)	Suffolk County Department of Health Services, 2011. Pump out and Soil Cleanup Criteria. http://www.aparo.com/files/deq_pc_sc_sanitarycode_article12_SOP.pdf (accessed 22.01.14).
State of North Carolina	North Carolina Department of Environment and Natural Resources (2005, 2012, 2013)	North Carolina Department of Environment and Natural Resources, 2005. "Contained-in" Policy for Soil Contaminated with Listed Hazardous Waste. http://portal.ncdenr.org/c/document_library/get_file?p_l_id=38491&folderId=328599&name=DLFE-9853.pdf (accessed 16.01.14).
		North Carolina Department of Environment and Natural Resources, 2012. Maximum Soil Contaminant Concentration Levels (MSCCs). http://portal.ncdenr.org/web/wm/ust/guidance#OTHRCAB (accessed 16.01.14).
		North Carolina Department of Environment and Natural Resources, 2013. Inactive Hazardous Sites Branch Preliminary Soil Remediation Goals (PSRG) Table. http://portal.ncdenr.org/c/document_library/get_file?uuid=5539ecfb-739f-4345-9459-b514508135f1&groupId=38361 (accessed 16.01.14).
State of Ohio	Ohio Administrative Code (2009)	Ohio Administrative Code, 2009. 3745-300-08 Generic numerical standards. http://epa.ohio.gov/Portals/30/rules/2012/Rule%203745-300-08.pdf (accessed 17.01.14).
	Ohio Environmental Protection Agency (2005)	Ohio Environmental Protection Agency, 2005. Table 3, Use of Risk-Based Numbers in the Remedial Response Process Overview. http://www.epa.ohio.gov/portals/30/rules/Use%20of%20Risk-Based%20Numbers%20in%20RR%20Process.pdf (accessed 17.01.14).
		Ohio Environmental Protection Agency, XXXX. Voluntary Action Program Chemical Information Database and Applicable Regulatory Standards (CIDARS). http://www.epa.state.oh.us/portals/30/rules/CIDARS%20Database.xls (accessed 17.01.14).
	U.S. Environmental Protection Agency (2013)	U.S. Environmental Protection Agency, 2013. Risk-Based Screening Table. http://www.epa.gov/reg3hwmd/risk/human/rb-concentration_table/Generic_Tables/index.htm (accessed 10.01.14).
State of Oklahoma	Oklahoma Department of Environmental Quality (2013)	Oklahoma Department of Environmental Quality, 2013. Risk-Based Decision Making for Site Cleanup. http://www.deq.state.ok.us/factsheets/land/SiteCleanUp.pdf (17.01.14).
	U.S. Environmental Protection Agency (2013)	
State of Oregon	Oregon Department of Environmental Quality (2010, 2012)	Oregon Department of Environmental Quality, 2010. Appendix D: Data Reporting and Screening Table. http://www.deq.state.or.us/lq/cu/stmwtrguidance.htm (accessed 17.01.14).
		Oregon Department of Environmental Quality, 2012. Risk-Based Concentrations DEQ RBCs Table. http://www.deq.state.or.us/lq/rbdm.htm (accessed 17.01.14).
	U.S. Environmental Protection Agency (2013)	U.S. Environmental Protection Agency, 2013. Risk-Based Screening Table. http://www.epa.gov/reg3hwmd/risk/human/rb-concentration_table/Generic_Tables/index.htm (accessed 10.01.14).
Commonwealth of Pennsylvania	Pennsylvania Department of Environmental Protection (2014)	Pennsylvania Department of Environmental Protection, 2014. Table 3a, Statewide Health Standards. http://www.portal.state.pa.us/portal/server.pt/community/land_recycling_program/20541/statewide_health_standards/1034862 (accessed 18.01.14).
Rhode Island	Rhode Island Department of Environmental Management (2011)	Rhode Island Department of Environmental Management, 2011. TABLE 1, Rules and Regulations for the Investigation and Remediation of Hazardous Material Releases. http://www.dem.ri.gov/pubs/regs/regs/waste/remreg11.pdf (accessed 18.01.14).

State of Tennessee	Tennessee Department of Environment and Conservation (2001)	Tennessee Department of Environment and Conservation, 2001. Standard Operating Procedure for the State Remediation Program. http://www.tn.gov/environment/docs/dor/srpsop.pdf (accessed 18.01.14).
State of Texas	Texas Commission on Environmental Quality (2003, 2006a,b, 2012)	Texas Commission on Environmental Quality, 2003. Subchapter S: Risk Reduction Standards, Industrial Solid Waste and Municipal Hazardous Waste. http://www.tceq.texas.gov/assets/public/legal/rules/rules/pdflib/335s.pdf (accessed 21.01.14).
		Texas Commission on Environmental Quality, 2006a. Appendix II e Medium-specific Concentrations, Risk Reduction Rule, Cleanup Standards, Updated Examples of Standard No. 2. http://www.tceq.texas.gov/remediation/rrr.html/#topic2 (accessed 21.01.14).
		Texas Commission on Environmental Quality, 2012. Table 4 Combined Tier 1 Soil PCLs and Table 9 Individual RBELs, TRRP Protective Concentration Levels. http://www.tceq.texas.gov/assets/public/remediation/trrp/trrppcls1_5.pdf (accessed 21.01.14).
State of Vermont	Vermont Department of Environmental Conservation (2012)	Vermont Department of Environmental Conservation, 2012. Soil Screening Values (SSV) Table, Investigation and Remediation of Contaminated Properties Procedure. http://www.anr.state.vt.us/dec/wastediv/sms/pubs/IROCP.pdf (accessed 21.01.14).
	U.S. Environmental Protection Agency (2013)	U.S. Environmental Protection Agency, 2013. Risk-Based Screening Table. http://www.epa.gov/reg3hwmd/risk/human/rb-concentration_table/Generic_Tables/index.htm (accessed 10.01.14).
Commonwealth of Virginia	Virginia Department of Environmental Control (undated)	Commonwealth of Virginia, Virginia Department of Environmental Control. Table 2.5 Selection of Contaminants of Concern Soil: Unrestricted (Residential). http://www.deq.virginia.gov/Programs/LandProtectionRevitalization/RemediationProgram/VoluntaryRemediationProgram/VRPRiskAssessmentGuidance/Tables.aspx (accessed 21.01.14).
State of Washington	Washington Department of Ecology (2007, 2014)	Washington Department of Ecology, 2007. WAC 173-340-900, Table 740-1, Method A. https://fortress.wa.gov/ecy/clarc/FocusSheets/Method%20A%20Notes%20Soil%20Unrestricted.pdf (accessed 21.01.14).
		Washington Department of Ecology, 2014. Cleanup Levels and Risk Calculation Database Search. https://fortress.wa.gov/ecy/clarc/CLARCsitemap.aspx (accessed 21.01.14).
State of West Virginia	West Virginia Department of Environmental Protection (2009a,b)	West Virginia Department of Environmental Protection, 2009a. Table 60-3B Series 3 Voluntary Remediation and Redevelopment Rule. http://www.dep.wv.gov/dlr/oer/voluntarymain/Documents/60CSR3%20VRRA%20filed%204-11-12.pdf (accessed 21.01.14).
		West Virginia Department of Environmental Protection, 2009b. West Virginia Voluntary Remediation and Redevelopment Act. 2009b. http://www.dep.wv.gov/dlr/oer/voluntarymain/Documents/VRRA%20GuidanceVersion2-1.pdf (accessed 21.01.14).
State of Wisconsin	Wisconsin Department of Natural Resources (2013)	Wisconsin Department of Natural Resources, 2013. Soil Residual Contaminant level determination using the U.S. EPA Regional Screening level Web Calculator. (accessed 22.01.14).
State of Wyoming	Wyoming Department of Environmental Quality (2013)	Wyoming Department of Environmental Quality, 2013. Cleanup Level Look-up Table. http://deq.state.wy.us/volremedi/downloads/Current%20Fact%20Sheets/cleanup_table.pdf (accessed 23.01.14).
		U.S. territories
Commonwealth of the Northern Mariana Islands	Commonwealth of the Northern Mariana Islands Division of Environmental Quality (2012)	Commonwealth of the Northern Mariana Islands Division of Environmental Quality, 2012. Appendix 1: Detailed Lookup Tables, Volume 2: Background Documentation for the Development of Tier 1 Environmental Action Levels, Evaluation of Environmental Hazards at Sites with Contaminated Soil and Groundwater. http://www.deq.gov.mp/ (accessed 24.01.14).
Unincorporated Territory of Guam	Guam Environmental Protection Agency (2012)	Guam Environmental Protection Agency, 2012. Appendix 1: Detailed Lookup Tables, Volume 2: Background Documentation for the Development of Tier 1 Environmental Action Levels, Evaluation of Environmental Hazards at Sites with Contaminated Soil and Groundwater. http://www.guamepa.govguam/net/ (accessed 24.01.14).

Table S4. U.S. pesticide drinking water regulatory jurisdictions references and dates accessed.

Jurisdictions	Sources	Website References
U.S. national jurisdictions U.S. national jurisdictions		
U.S. Environmental Protection Agency	U.S. Environmental Protection Agency (2009)	U.S. Environmental Protection Agency, 2009. National Primary Drinking Water Regulations List of Contaminants and their (MCLs). http://water.epa.gov/drink/contaminants/index.cfm#Organic (Accessed March 22. 2014).
Food and Drug Administration	Food and Drug Administration (2013)	Food and Drug Administration, 2013. Bottled Water & Carbonated Soft Drinks Guidance Documents & Regulatory Information 2013 21 CFR Part 165.110 - Bottled Water. http://www.ecfr.gov/cgi-bin/text-idx?c=ecfr&sid=e8b7a7992ba5413d50490c47e2f2e69b&rgn=div5&view=text&node=21:2.0.1.1.38&idno=21#21:2.0.1.1.38.2.1.1 (Accessed April 2. 2014).
U.S. Army Public Health Command	U.S. Army Public Health Command (2013)	U.S. Army Public Health Command, 2013. Technical Guide 230 Environmental Health Risk Assessment and Chemical Exposure Guidelines for Deployed Military Personnel 2013. http://phc.amedd.army.mil/PHC%20Resource%20Library/TG230.pdf (Accessed April 4. 2014).
U.S. state and regional jurisdictions		
State of Alabama	Alabama Department of environmental management (undated)	Alabama department of environmental management. Drinking Water. http://www.adem.state.al.us/programs/water/drinkingwater.cnt (Accessed March 22. 2014).
State of Alaska	Alaska Department of Environmental Conservation (2008)	Alaska Department of Environmental Conservation, 2008. Water Quality Criteria Manual for Toxics. http://dec.alaska.gov/water/wqsar/wqs/index.htm (Accessed March 22. 2014).
State of Arizona	Arizona Department of Environmental Quality (2008)	Arizona Department of Environmental Quality, 2008. Title 18, Chapter 4, Ariz. Admin. Code ADEQ's Safe Drinking Water Regulations. http://www.azdeq.gov/environ/water/dw/download/dw_rules.pdf (Accessed March 22. 2014).
State of Arkansas	Arkansas Department of Environmental Quality (2013)	Arkansas Department of Environmental Quality, 2013. Water Quality Standards Review Regulation No. 2 Water Division. http://www.adeq.state.ar.us/ftproot/Pub/WebDatabases/PermitsOnline/NPDES/PermitInformation/AR0038857_EPA%20Primary%20Drinking%20Water%20Standards_20030601.pdf (Accessed March 22. 2014).
State of California	California Office of Environment Health Hazard Assessment (2010)	California Office of Environment Health Hazard Assessment, 2010. HISTORIC CDPH “ACTION LEVELS” (now called “Notification Levels”) FOR CHEMICALS WITH MCLS. http://www.cdph.ca.gov/certlic/drinkingwater/Documents/Notificationlevels/priorNLnowMCL.pdf (Accessed 23. 2014).
	California Department of Public Health (2010)	California Department of Public Health, 2010. Drinking Water Notification Levels and Response Levels: An Overview. http://www.cdph.ca.gov/certlic/drinkingwater/Documents/Notificationlevels/notificationlevels.pdf (Accessed March 23. 2014).
	California Department of Health Services (2010)	California Department of Health Services, 2010. CDPH’S Archived Advisory Levels for Drinking Water. http://www.cdph.ca.gov/certlic/drinkingwater/Documents/Notificationlevels/archivedadvisorylevels.pdf (Accessed March 23. 2014).
	California Department of Health Services (2014)	California Department of Health Services, 2014. Maximum Contaminant Levels and Regulatory Dates for Drinking Water U.S. EPA vs California February. http://www.cdph.ca.gov/certlic/drinkingwater/Pages/Chemicalcontaminants.aspx (Accessed March 23. 2014).
State of Colorado	Colorado Department of Public Health and Environment (2014)	Colorado Department of Public Health and Environment, 2014. Regulation No. 11 Colorado Primary Drinking Water Regulations. http://www.colorado.gov/cs/Satellite?blobcol=urldata&blobheadername1=Content-Disposition&blobheadername2=Content-Type&blobheadervalue1=inline%3B+filename%3D%22Regulation+11.pdf%22&blobheadervalue2=application%2Fpdf&blobkey=id&blobtable=MungoBlobs&blobwhere=1251924799151&ssbinary=true (Accessed March 24. 2014).
State of Connecticut	Connecticut Department of energy and environmental protection (2013)	Connecticut Department of energy and environmental protection, 2013. Action Level List for Private Wells. http://www.ct.gov/dph/lib/dph/environmental_health/eoha/pdf/pw_action_levels.pdf (Accessed March 24. 2014).

	Connecticut Department of energy and environmental protection (2014)	Connecticut Department of energy and environmental protection, 2014. Potable Water Program. http://www.ct.gov/deep/cwp/view.asp?a=2715&q=324998&deepNav_GID=1626 (Accessed March 24. 2014).
State of Delaware	Delaware Department of Natural Resources and Environmental Control (undated)	Delaware Department of Natural Resources and Environmental Control. Drinking Water Quality. http://www.delaware.gov/waterquality/drinkingwaterquality.shtml (Accessed March 24. 2014).
State of Florida	Florida Department of Health (2014)	Florida Department of Health, 2014. Chemical Contaminants - HALs and Chemical Fact Sheets. http://www.floridahealth.gov/healthy-environments/drinking-water/Chemicals-HALs.html (Accessed March 24. 2014).
State of Hawaii	Hawaii Department of Health (2009)	Hawaii Department of Health, 2009. Contaminants Regulated by the Safe Drinking Water Branch (effective 3/4/09). http://health.hawaii.gov/sdwb/files/2013/08/MCL_03-04-09.pdf (Accessed March 25. 2014).
State of Idaho	Idaho Department of Environmental Quality (2014)	Idaho Department of Environmental Quality, 2014. Contaminants in Drinking Water. http://www.deq.idaho.gov/water-quality/drinking-water/pws-monitoring-reporting/contaminants.aspx (Accessed March 25. 2014).
State of Illinois	Illinois Environmental Protection Agency (undated)	Illinois Environmental Protection Agency. WATER QUALITY STANDARDS SUBPART C: PUBLIC AND FOOD PROCESSING WATER SUPPLY STANDARDS. http://www.ipcb.state.il.us/documents/dsweb/Get/Document-33354 (Accessed March 25. 2014).
State of Indiana	Indiana Department of Environmental Management (undated)	Indiana Department of Environmental Management. Source Water Protection - Drinking Water. http://www.in.gov/idem/4142.htm (Accessed March 25. 2014).
	Indiana Department of Environmental Management (1996)	Indiana Department of Environmental Management, 1996. Article 8. Public Water Supply. http://www.in.gov/idem/4868.htm (Accessed March 25. 2014).
State of Iowa	Iowa Department of Nature Resources (2012)	Iowa Department of Nature Resources, 2012. Public Drinking Water Program. http://www.iowadnr.gov/Portals/idnr/uploads/water/wse/2012SAR.pdf (Accessed March 26. 2014).
State of Kansas	Kansas Department of Health and Environment (2004)	Kansas Department of Health and Environment, 2004. Article 15.—Application for Permits; Domestic Water Supply. http://www.kdheks.gov/pws/regs/drinking_water_regs.pdf (Accessed March 26. 2014).
Commonwealth of Kentucky	Kentucky Department of environmental protection (2010)	Kentucky Department of environmental protection, 2010. Safe Drinking Water Act-a summary. http://water.ky.gov/Fact%20Sheets/Safe%20Drinking%20Water%20Act.pdf (Accessed March 26. 2014).
State of Louisiana	Louisiana Department of Health and Hospital (undated)	Louisiana Department of Health and Hospital. Safe Drinking Water Program. http://new.dhh.louisiana.gov/index.cfm/page/963 (Accessed March 26. 2014).
State of Maine	Government of Maine (undated)	Government of Maine. http://www.maine.gov/dhhs/mecdc/environmental-health/water/rules-policies/rules-policieshome.htm (Accessed March 26. 2014).
State of Maryland	Maryland Department of Environment (undated)	Maryland Department of Environment. http://www.mde.state.md.us/programs/Water/Water_Supply/Pages/Regulations.aspx (Accessed March 26. 2014).
Commonwealth of Massachusetts	Massachusetts Office of Energy and Environmental Affairs Energy and Environmental Affairs (2012)	Massachusetts Office of Energy and Environmental Affairs Energy and Environmental Affairs, 2012. Standards & Guidelines for Contaminants in Massachusetts Drinking Water. http://www.mass.gov/eea/agencies/massdep/water/drinking/standards/standards-and-guidelines-for-drinking-water-contaminants.html (Accessed March 26. 2014).
State of Michigan	Michigan Department of Environmental Quality (2014)	Michigan Department of Environmental Quality, 2014. Community Water Supply. http://www.michigan.gov/deq/0,4561,7-135-3313_3675_3691---,00.html (Accessed March 26. 2014).
State of Minnesota	Minnesota Department of Health (2011)	Minnesota Department of Health, 2011. Drinking Water Standards for Contaminants: Volatile and Synthetic Organic Chemicals. http://www.health.state.mn.us/divs/eh/water/factsheet/com/voc_soc.pdf (Accessed March 27. 2014).
State of Mississippi	Mississippi Department of Health (2013)	Mississippi Department of Health, 2013. The Public Water Supply Program ensures safe drinking water to the 2.8 million citizens of Mississippi who utilize the state's public water supplies by strictly enforcing the requirements of the Federal and State Safe Drinking Water Acts (SDWAs). http://msdh.ms.gov/msdhsite/index.cfm/44,0,76,html (Accessed March 27. 2014).

State of Missouri	Missouri Department of Natural Resources (1996)	Missouri Department of Natural Resources, 1996. Rules of Department of Natural Resources Division 60—Safe Drinking Water Commission Chapter 4—Contaminant Levels and Monitoring. http://www.sos.mo.gov/adrules/csr/current/10csr/10c60-4.pdf (Accessed March 27. 2014).
State of Montana	Montana Department of Environmental Quality (2004)	Montana Department of Environmental Quality, 2004. Drinking water wells in Montana. http://deq.mt.gov/wqinfo/swp/Guidance.mcpx (Accessed March 27. 2014).
State of Nebraska	Nebraska Department of Health and Human Services (2012)	NEBRASKA DEPARTMENT OF HEALTH AND HUMAN SERVICES, 2012. DRINKING WATER STANDARDS AND TREATMENT TECHNIQUES. http://www.sos.ne.gov/rules-and-regs/regsearch/Rules/Health_and_Human_Services_System/Title-179/Chapter-12.pdf (Accessed March 27. 2014).
State of Nevada	Nevada Division of Environmental Protection (2013)	Nevada Division of Environmental Protection, 2013. Maximum Contaminant Levels (MCL's). http://ndep.nv.gov/bsdw/docs/approved-analytica_methods.pdf (Accessed March 27. 2014).
State of New Hampshire	New Hampshire Department of Environmental Services (2013)	New Hampshire Department of Environmental Services, 2013. CHAPTER ENV-DW 700 Water Quality: Standards, Monitoring, Treatment, Compliance, and Reporting. http://des.nh.gov/organization/commissioner/legal/rules/documents/env-dw702-706.pdf (Accessed March 27. 2014).
State of New Jersey	New Jersey Department of Environmental Protection (2011)	New Jersey Department of Environmental Protection, 2011. N.J.A.C. 7:10 Safe Drinking Water Act Rules. http://www.nj.gov/dep/rules/rules/njac7_10.pdf (Accessed March 28. 2014).
	New Jersey Department of Environmental Protection (2009)	New Jersey Department of Environmental Protection, 2009. Drinking Water Standards by Constituent. http://www.nj.gov/dep/standards/drinking%20water.pdf (Accessed March 28. 2014).
State of New Mexico	New Mexico Environment Department (2003)	New Mexico Environment Department, 2003. http://www.nmenv.state.nm.us/dwb/contaminants/documents/MCLs.pdf (Accessed March 28. 2014).
State of New York	New York Department of Health (2011)	New York Department of Health, 2011. Part 5, Subpart 5-1 Public Water Systems – Tables. http://www.health.ny.gov/regulations/nycrr/title_10/part_5/subpart_5-1_tables.htm (Accessed March 28. 2014).
State of North Carolina	North Carolina Division of Water Resources (2011)	North Carolina Division of Water Resources, 2011. North Carolina's Annual State Public Water Systems Compliance Report For the Calendar Year 2010. http://www.ncwater.org/files/pws/annual/2010AnnualComplianceReport.pdf (Accessed March 31. 2014).
State of North Dakota	North Dakota Department of Health (2005)	North Dakota Department of Health, 2005. Drinking Water Program. http://www.ndhealth.gov/mf/dw.html (Accessed March 31. 2014).
State of Ohio	Ohio Environmental Protection Agency (undated)	Ohio Environmental Protection Agency. Rules, Laws, Policies and Guidance. http://epa.ohio.gov/ddagw/rules.aspx (Accessed March 31. 2014).
State of Oklahoma	Oklahoma Department of Environmental Quality (2012)	Oklahoma Department of Environmental Quality, 2012. ITLE 252. Department of Environmental Quality Chapter 626. Public Water Supply Construction Standards. http://www.deq.state.ok.us/rules/626.pdf (Accessed March 31. 2014).
State of Oregon	Oregon Department of Environmental Quality (2000)	Oregon Department of Environmental Quality, 2000. Oregon Drinking Water Quality Standards. http://public.health.oregon.gov/healthyenvironments/drinkingwater/operations/documents/pipeline/pipesp00.pdf (Accessed March 31. 2014).
	Oregon Public Health (2012)	Oregon Public Health, 2012. Study of Pesticides in School Drinking Water. https://public.health.oregon.gov/HealthyEnvironments/DrinkingWater/SourceWater/Pages/pdp-schools.aspx (Accessed March 31. 2014).
Commonwealth of Pennsylvania	Pennsylvania Department of Environmental Protection (2006)	Pennsylvania Department of Environmental Protection, 2006. Pennsylvania Department of Environmental Protection Division of Drinking Water Management Maximum Contaminant Levels (MCLs). http://www.portal.state.pa.us/portal/server.pt/community/Drinking_Water_Regulations,_Standards_&_Resources/21161#Standards (Accessed March 31. 2014).
Rhode Island	Rhode Island Department of Health (2011)	Rhode Island Department of Health, 2011. Capacity Development Annual Implementation Report 2011. http://www.health.ri.gov/publications/programreports/2011DrinkingWaterCapacityDevelopment.pdf (Accessed April 1. 2014).

State of South Carolina	South Carolina Department of Health and Environment (2009)	South Carolina Department of Health and Environment, 2009. State Primary Drinking Water Regulation: R.61-58. http://www.scdhec.gov/environment/water/regs/r61-58.pdf (Accessed April 1. 2014).
State of South Dakota	South Dakota Department of Environment and Natural Resources (undated)	South Dakota Department of Environment and Natural Resources. 40 C.F.R. PART 141 – NATIONAL PRIMARY DRINKING WATER REGULATIONS. https://denr.sd.gov/des/dw/dwregs.aspx (Accessed April 1. 2014).
State of Tennessee	Tennessee Department of Environment and Conservation (undated)	Tennessee Department of Environment and Conservation. Drinking Water Program. http://www.tn.gov/environment/water/water-supply_drinking-water-program.shtml (Accessed April 2. 2014).
State of Texas	Texas Commission on Environmental Quality (2013)	Texas Commission on Environmental Quality, 2013. Consumer Confidence Reports: What's In Your Drinking Water. http://www.tceq.texas.gov/publications/pd/020/2013-NaturalOutlook/consumer-confidence-reports-whats-in-your-drinking-water (Accessed April 2. 2014).
State of Utah	Utah Department of Environmental Quality (2014)	Utah Department of Environmental Quality, 2014. R309. Environmental Quality, Drinking Water. Rule R309-200. Monitoring and Water Quality: Drinking Water Standards. http://www.rules.utah.gov/publicat/code/r309/r309-200.htm#T5 (Accessed April 2. 2014).
State of Vermont	Vermont Agency of Natural Resources (2010)	Vermont Agency of Natural Resources, 2010. Environmental Protection Rules Chapter 21 Water Supply Rule. http://drinkingwater.vt.gov/dwrules/pdf/vtwsr2010.pdf#zoom=100 (Accessed April 2. 2014).
	Vermont Department of Health (2002)	Vermont Department of Health, 2002. Vermont Department of Health Drinking Water Guidance. http://healthvermont.gov/enviro/water/documents/drinkwaterguidance02.pdf (Accessed April 2. 2014).
Commonwealth of Virginia	Virginia Department of Health (2014)	Virginia Department of Health, 2014. Safe Drinking Water Act (SDWA): 42 U.S.C. §300f et seq. http://www.vdh.state.va.us/odw/laws_regs.htm (Accessed April 2. 2014).
State of West Virginia	Business and Legal Resources (2014)	Business and Legal Resources, 2014. West Virginia Drinking Water: What you need to know. http://www.blr.com/Environmental/Water/Drinking-Water-in-West-Virginia (Accessed April 2. 2014).
State of Wisconsin	Wisconsin Department of Natural Resources (2013)	Wisconsin Department of Natural Resources, 2013. Chapter NR 809 Safe Drinking Water. Subchapter I — Maximum Contaminant Levels, Monitoring and Analytical Requirements for Primary Drinking Water Contaminants. https://docs.legis.wisconsin.gov/code/admin_code/nr/800/809/ (Accessed April 2. 2014).
State of Wyoming	Wyoming Department of Environmental Quality (2013)	Wyoming Department of Environmental Quality, 2013. Water Quality Rules and Regulations Chapter 1. http://deq.state.wy.us/wqd/wqdrules/Chapter_01.pdf (Accessed April 2. 2014).
	U.S. territories	
Commonwealth of the Northern Mariana Islands	CNMI Division of Environmental Quality (2005)	CNMI Division of Environmental Quality, 2005. Commonwealth of the Northern Mariana Islands Water Quality Standards. http://water.epa.gov/scitech/swguidance/standards/upload/2005_01_13_standards_wqslibrary_territories_northern_mariana_9_wqs.pdf (Accessed September 26. 2014).
Unincorporated Territory of Guam	Guam Environmental Protection Agency (1997)	Guam Environmental Protection Agency, 1997. Guam Primary Safe Drinking Water Regulations. http://www.guamcourts.org/CompilerofLaws/GAR/22GAR/22GAR002-6.pdf (Accessed September 26. 2014).
